# Afterglow quenching in plasma-based dry reforming of methane: a detailed analysis of the post-plasma chemistry *via* kinetic modelling†

**DOI:** 10.1039/d4su00676c

**Published:** 2025-01-28

**Authors:** Joachim Slaets, Eduardo Morais, Annemie Bogaerts

**Affiliations:** a Research Group PLASMANT, Department of Chemistry, University of Antwerp Universiteitsplein 1 BE-2610 Wilrijk-Antwerp Belgium annemie.bogaerts@uantwerpen.be

## Abstract

We have developed a kinetic model to investigate the post-plasma (afterglow) chemistry of dry reforming of methane (DRM) in warm plasmas with varying CO_2_/CH_4_ ratios. We used two methods to study the effects of plasma temperature and afterglow quenching on the CO_2_ and CH_4_ conversion and product selectivity. First, quenching *via* conductive cooling is shown to be unimportant for mixtures with 30/70 and 50/50 CO_2_/CH_4_ ratios, while it affects mixtures containing excess CO_2_ (70/30) by influencing radical recombination towards CO_2_, H_2_ and H_2_O, as well as the water gas shift reaction, decreasing the CO_2_ conversion throughout the afterglow. This is accompanied by shifts in product distribution, from CO and H_2_O to CO_2_ and H_2_, and the magnitude of this effect depends on a combination of plasma temperature and quenching rate. Second and more importantly, quenching *via* post-plasma mixing of the hot plasma effluent with fresh cold gas yields a significant improvement in conversion according to our model, with 258% and 301% extra conversion for CO_2_ and CH_4_, respectively. This is accompanied by small changes in product selectivity, which are the result of interrupted reaction pathways at lower gas temperatures in the afterglow. Effectively, the post-plasma mixing can function as a heat recovery system, significantly lowering the energy cost through the additional conversion ensued. With this approach, our model predicts that energy consumption can be lowered by nearly 80% in comparison to DRM under the same plasma conditions without mixing.

## Abstract 2

Chemical kinetics modelling of the afterglow of a CO_2_–CH_4_ plasma reveals that cooling in excess CO_2_ alters the product distribution through the water gas shift reaction, while mixing of cold gas can improve the conversion by using the residual heat.

## Graphical abstract

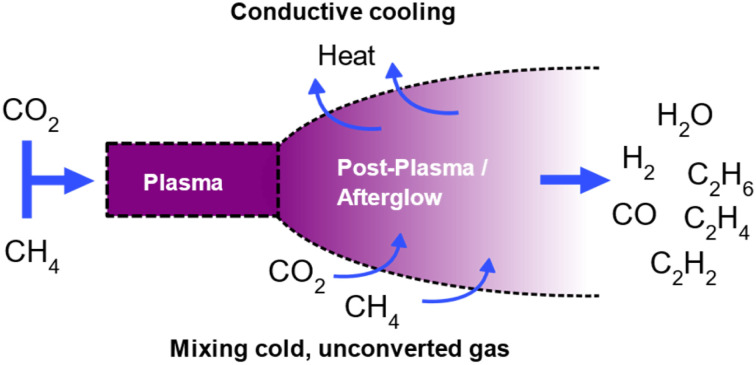

**Sustainability spotlight**

This paper describes plasma-based dry reforming of methane (DRM), converting CO_2_ and CH_4_ into CO and H_2_, which can be used to produce more complex chemicals. This supports a circular economy, by using CO_2_ and biogas as a feedstock, while reducing emissions and dependence on fossil fuels. Plasma reactors are ideal in this scenario as they can operate on renewable electricity and easily couple to intermittent energy supplies. In this work, we evaluate different post-plasma quenching methods *via* chemical kinetics modelling to develop a better understanding of chemical pathways in plasma-based DRM, crucial to advance this technology and bring it closer to industry. Our work contributes to UN sustainable development goals: industry, innovation, and infrastructure (SDG-9) and climate action (SDG-13).

## 1. Introduction

CO_2_ and CH_4_ are both major greenhouse gases that play an important role in climate change. Therefore, reducing emissions of these gases is an important challenge. Beyond lower emissions, utilising these gases in new chemical processes can provide a sustainable source of raw materials, creating a circular economy.^1^ As such, targeting the dry reforming of methane (DRM) (R1) can help to convert CO_2_ and CH_4_ into more useful molecules, in this case, a mixture of CO and H_2_ (syngas). In turn, syngas can be used as a building block for more complex, value-added chemicals, for example in the Fischer–Tropsch process to synthesise a variety of hydrocarbons.^2–4^

R1 CO_2_ + CH_4_ → 2CO + 2H_2_, Δ*H*^0^ = 247 kJ mol^−1^

The use of plasma reactors for DRM offers many benefits. It is a fully electrified process, allowing the reactors to operate using renewable energy. Also, it can be quickly switched on/off, and scaled according to energy availability and demand, making it suitable for peak shaving.^5–7^ As a young and promising technology for gas conversion, plasma has been extensively researched in the last decade.

Considerable research effort goes into the optimisation of specific plasma parameters. Various distinct plasma types and reactor configurations have been studied under varying operating conditions – such as flow rate, applied power, reactor geometry, gas mixtures, *etc.*^6^ Warm plasma reactors, including gliding arc (GA), microwave (MW), atmospheric pressure glow discharges (APGDs) or nanosecond pulsed discharges (NPDs) can generate high gas temperatures, reaching several 1000 K.^5,6^ While the gas conversion is typically driven by thermal chemistry, they are more energy efficient than cold plasmas, like dielectric barrier discharges.^5,6,8^ In these warm plasmas, the downstream gas temperature (*i.e.*, the afterglow or post-plasma region, outside of the plasma zone) may still be sufficiently high to enable reaction pathways that can influence reactor performance in different manners. For instance, this effect could trigger reverse reactions, reducing the overall conversion and altering the product distribution.^9^ On the other hand, it can also be used to enhance the conversion and product yield.^9^ Thus, recent studies have increasingly focussed on the post-plasma region of these reactors, to build a better understanding of the effects at play and improve the overall performance.

In CO_2_ plasmas, it has been demonstrated that CO_2_ conversion locally within the plasma can approach 100%.^10,11^ However, reverse reactions in the post-plasma region (namely recombination of CO with O radicals) have been found to reduce this significantly, with measured conversions as low as 25%.^10,11^ Rapid quenching of the high gas temperature has been shown to be a successful strategy to curtail these reverse reactions.^12–15^ Three different modes can be identified for post-plasma quenching.^9^ First, in absolute quenching, the plasma-generated product molecules are preserved, while the radical species recombine to reform the reactants, *i.e.*, leading to a net reduction in the conversion. Second, in ideal quenching, the conversion achieved in the plasma zone is retained by inducing the radicals to react towards products. The third quenching mode is called super-ideal quenching, where not only the high conversions are maintained, but they can be further boosted. Super-ideal quenching can be achieved when the gas is cooled faster than the time required for vibrational–translational (VT) relaxations to occur, creating a VT non-equilibrium.^9^ The vibrational energy trapped in the gas molecules can stimulate endothermic reactions, allowing for conversion gains during the quenching. This super-ideal quenching mode will not be considered in this work, as it can only be attained under very specific conditions and in systems with a strong VT non-equilibrium character.^11^ This is not the case for the atmospheric plasmas studied in this work, as it has been shown that VT non-equilibrium is limited to pressures below or equal to 25 mbar.^16^

There is a diverse range of quenching methods and several studies have investigated the use of a constricting nozzle, for example.^13–15^ This device can cool down the gas by rapid expansion, creating eddies in the fluid flow which improve gas mixing and heat transfer.^14,15,17^ For CO_2_, the conversion gain from this strategy can vary significantly (between 2 and 30%), depending strongly on the operating conditions and nozzle design. The largest additional conversion was seen by Hecimovic *et al.*^15^ who measured an increase in CO_2_ conversion from 5 to 35%, following nozzle implementation. Subsequently, this setup was modelled by Van Alphen *et al.*^18^ confirming these findings, indicating that a cooling rate of ∼10^7^ K s^−1^ was achieved, significantly enhancing CO_2_ conversion. A similar nozzle approach was modelled by Yang *et al.*,^17^ reaching the same conclusions.

Further research attempted quenching using liquid-cooled devices to reduce the temperature of the gas stream from the plasma region. A two-stage cooling system used by Wang *et al.*^19^ improved the CO_2_ conversion from 6.6 to 19.5%. Another design, which uses a liquid-cooled rod in the reactor outlet to achieve the post-plasma cooling, was used by Kim *et al.*^12^ who reported that the CO_2_ conversion increased from 30.1 to 36.1% upon implementation of this strategy. The same approach was also tested for DRM, leading to interesting findings: both the CO_2_ and CH_4_ conversion were observed to drop compared to the uncooled plasma reactor.^20^ The reported maximum decrease in total conversion occurred at a CO_2_/CH_4_ ratio of 3/1, from 23.4 to 22.6%. On the other hand, upon employing the quenching rod, selectivity towards H_2_ was boosted, while that towards H_2_O dropped. The selectivity effect was attributed to the inhibition of the reverse water gas shift (RWGS) in the colder post-plasma region, preventing CO_2_ and H_2_ from reacting to CO and H_2_O. While the drop in conversion has a negative effect on the energy cost, the selectivity gain towards H_2_ (observed for mixtures with higher CH_4_ fractions) can outpace the negative effect on conversion, resulting in a lower energy cost for syngas production.

Another approach to consider is the introduction of new gas in the afterglow region, such as through a secondary inlet. Hyun Cho *et al.*^21^ injected cold CH_4_ in the afterglow of a CO_2_ plasma to achieve conversion, effectively separating the DRM reaction into a two step process. They claim the main advantage of this method is the increase in energy consumption selectivity. The energy injected through the plasma decomposes only CO_2_ rather than CH_4_, yielding higher CO_2_ conversion and higher syngas energy conversion efficiency compared to the direct DRM reaction in the plasma. While their work specifically focusses on this two step DRM process, this secondary inlet can be an interesting strategy to apply, even without changing the overall gas mixture. The remaining heat in the plasma effluent can be recovered and used to convert the newly introduced gas, potentially improving the performance of the system, while it also provides cooling to the afterglow.

While a comparison with current industrial processes involving DRM and quenching would provide valuable context for the broader application of this research, this theoretical study specifically focuses on understanding the fundamental mechanisms of post-plasma effects in warm plasmas, opening avenues for future experiments and benchmarking.

In this work, we aim to explore the effects of these post-plasma quenching methods on the chemical kinetics for DRM, and elucidate the mechanisms involved in the observed conversion and selectivity trends. Our model incorporates two distinct approaches to post-plasma quenching: (i) heat removal from the system (emulating the introduction of a cooled rod, hence conductive cooling), and (ii) the mixing of cold gas in the post-plasma region (emulating the introduction of a nozzle, or simply adding cold gas in the afterglow). In the interest of model versatility and relevance, we do not limit our work to specific reactor designs or operating conditions; instead we focus specifically on warm plasma conditions, which are indeed known to yield the best performance for DRM.^6^ To this end, we study a wide range of conditions, with plasma temperatures between 2000–4000 K and three different CO_2_/CH_4_ ratios, *i.e.*, stoichiometric (50/50), excess CH_4_ (30/70) and excess CO_2_ (70/30). We compare different degrees of gas cooling, achieved with both methods, and evaluate the effects of the ensuing temperature decrease on conversion, selectivity and energy cost. Our primary objective is to obtain a better understanding of the post-plasma kinetics upon gas cooling. These insights can help experimentalists towards potential improvements and new reactor designs for further advancement of plasma-based DRM technologies.

## 2. Model description

### 2.1. Simulation details

In this work, we use a zero-dimensional chemical kinetics model which allows us to conduct an in-depth (yet general) study of multiple conditions and approaches, while also running simulations independent of a specific reactor configuration. The calculations were performed using the ZDPlasKin^22^ code, which is described in Section 2.3.

**Fig. 1:**
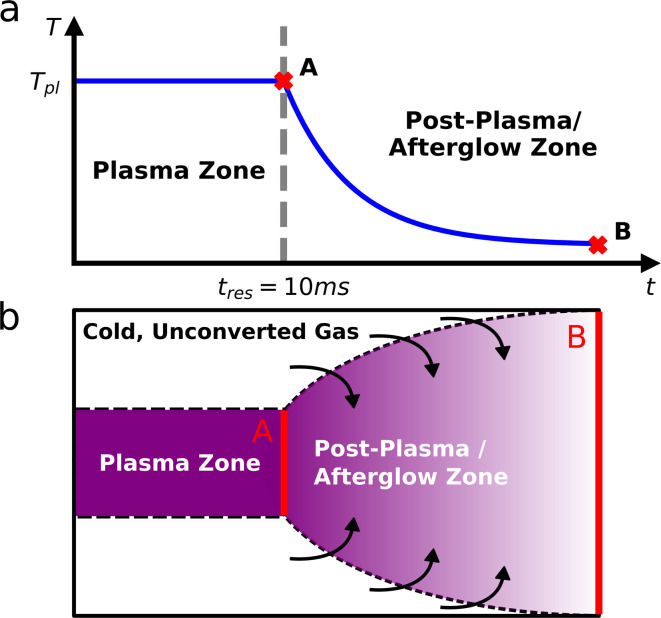
(a) Schematic overview of the simulation domain, showing the plasma and post-plasma/afterglow zones, plasma temperature (*T*_pl_) and residence time (*t*_res_, which is typically around 10 ms).^23,24^ (b) Schematic overview of the enhanced mixing approach, indicating the plasma and post-plasma/afterglow zones. The cold unconverted gas is only added after the plasma, either from a peripheral region around the plasma zone or from a secondary inlet. The important points in the simulations are compared in the Results and discussion section. (A) Corresponds to the end of the plasma zone (at the plasma temperature, immediately before the temperature drop), and (B) to the end of the afterglow after all quenching has taken place.

The simulation domain consists of a plasma region and a subsequent post-plasma (or afterglow) region, with the latter being the main focus of this work. A schematic overview of the simulations is given in Fig. 1a, illustrating the plasma zone with a constant temperature, followed by the afterglow in which the gas temperature decreases, as the hot gas is quenched. We simulate (i) fast conductive cooling and (ii) mixing with cold unconverted gas (see Fig. 1b) as distinct quenching methods.

In the plasma, gas temperatures between 2000–4000 K are assumed, which is the typical temperature range for many warm plasmas.^6^ The residence time in the plasma zone was set to 10 ms, which is a reasonable assumption following the works of Van Alphen *et al.*^23^ and Dahl *et al.*^24^ These conditions are also consistent with our previous work^25^ (focused on DRM plasma kinetics), which demonstrated that within the studied temperature range, the kinetics is dominated by thermal chemistry, meaning that electron impact processes can be neglected. Therefore, the DRM thermal kinetics suffices to describe the plasma region, simplifying the simulations. In terms of applicability, this approach broadens the potential of our results, since the gained insights can be expanded beyond plasma-specific conditions. To demonstrate the effects in the post-plasma region, we present the important parameters (species concentrations, conversion and selectivity) at points A and/or B (indicated in Fig. 1), as a function of the plasma temperature.

As mentioned above, two quenching approaches are tested within the post-plasma region, which divides our work into two main parts: (i) in the first approach, we model an afterglow system which is cooled through conductive heat loss (from point A to point B), aiming to study the effects of temperature decrease on the reaction kinetics. This conductive cooling is enhanced with a factor, *c*, (1, 10 and 100) with more details given in Section 2.3. (ii) In the second approach, the cooling stems from mixing room-temperature gas with the hot afterglow, introducing ‘fresh’ and ‘cold’ gas molecules which will reduce the overall gas temperature. In this study, the cold gas mixture introduced post-plasma is identical to the unconverted gas mixture. The freshly added CO_2_ and CH_4_ molecules will be dissociated by the relatively high temperatures in the afterglow, resulting in altered kinetic pathways and extra overall conversion.

Since this mixing approach does not consider conductive heat loss to the reactor walls, *i.e.*, the post-plasma region is assumed to be perfectly thermally insulated, the addition of cold gas is the only factor that influences the gas temperature and in turn the kinetics. Hence, without other means to decrease the gas temperature, optimal conditions are created to attain the highest possible conversion of the added gas. This is due to the redistribution of the available energy over more gas molecules, since this quenching method does not remove heat from the system. Thus, to guarantee a realistic cooling and a temperature drop sufficient to stop all reactions, a large amount of cold gas must be added. In our case, we found that adding a cold gas stream nine times larger than the initial flow (*i.e.*, diluting the fraction that travelled through the plasma to 10%) meets this criterion. We change the mixing time between 1, 10 and 100 ms to modulate the cooling strength, which is further explained in Section 2.3. This results in temperature gradients in the afterglow similar to the conductive cooling approach.

By studying these two quenching approaches in our model, *i.e.*, the enhanced thermal conductivity (or fast conductive cooling) and enhanced post-plasma mixing, we aim to provide insights into the reaction kinetics in post-plasma DRM processes. We note that these approaches are not directly comparable to experimental conditions, thus the trends and general findings resulting from the model are more relevant than the absolute values.

### 2.2. DRM chemistry

The species and reaction scheme used in this work is the same as in our previous work.^25^ However, as mentioned above, based on our previous study, our present simulations only consider thermal kinetics; hence electrons and ions and their respective reactions are not included. This amounts to a total of 40 species and 728 reactions. These species are listed in Table 1, and a list of the reactions with the corresponding rate coefficients and respective references is provided in the ESI (Table S3, at the end of the document).† The main dissociation pathways under the tested plasma conditions vary depending on the gas composition.^25^ For CO_2_, dissociation primarily occurs through reactions with H radicals, while in mixtures with an excess of CO_2_, reactions with O and OH radicals also contribute significantly. In the case of CH_4_, dissociation predominantly proceeds through reactions with H or neutral species (M). Additionally, in mixtures with excess CO_2_, OH radicals play a role, and in mixtures with excess CH_4_, C_2_H radicals also contribute to the dissociation process.

**Table 1:** Species included in the chemical kinetics set

C
O, O_2_, O_3_
H, H_2_
CO, CO_2_
CH, CH_2_, CH_3_, CH_4_, C_2_H, C_2_H_2_, C_2_H_3_, C_2_H_4_, C_2_H_5_, C_2_H_6_
OH, H_2_O, HO_2_, H_2_O_2_
CH_2_CH_2_OH, CH_2_CO, CH_2_OH, CH_3_CH_2_O, CH_3_CH_2_OH, CH_3_CHO, CH_3_CHOH, CH_3_CO, CH_3_COOH, HCCO, CH_3_O, CH_3_OH, CH_3_OO, CH_3_OOH, COOH, HCHO, HCO, HCOOH

### 2.3. Equations in the model

The calculations are performed using the ZDPlasKin^22^ code, which uses the DVODE solver^26^ to solve the mass conservation equation for each species included in the model (eqn (1)). The first term on the right-hand side calculates the change in number density with respect to time (∂*n*/∂*t*) for species (*s*) due to a chemical reaction (*i*). *a*^R^_*s*,*i*_ and *a*^L^_*s*,*i*_ are the coefficients of species *s* on the right and left side of reaction *i*, respectively, and *R*_*i*_ is the corresponding reaction rate, which is explained in ESI (Section S1 and eqn (S1)).† More details on the reaction rates, alongside a complete list of all reactions in the kinetics scheme and corresponding references, are given in ESI (Table S3 and Section S2).†1
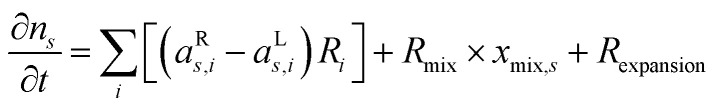


The standard mass conservation expression (based on the first term on the right-hand side) is expanded with two other terms to include an additional source term and a correction for gas expansion (see ESI, Section S1 and eqn (S2)†). The former is used to introduce new species in the system upon mixing with unconverted gas (as explained in Section 2.1) based on a rate (*R*_mix_) and the fraction added of each species in the model (*x*_mix,*s*_). These species are limited to CO_2_ and CH_4_, and their fractions are defined by the CO_2_/CH_4_ gas mixing ratio, *i.e.*, 30/70, 50/50 and 70/30. The mixing rate (*R*_mix_) is defined as a source term that represents species transport from the surrounding cold unconverted gas flow into the modelled plasma effluent volume (as shown in Fig. 1b). This is given in eqn (2), where *n*_mix_ is the total number density of gas that is added during the mixing process, *τ*_mix_ is the characteristic mixing time, *t*_AG_ is the time in the afterglow and *α* is the gas expansion factor. The dilution of 10% (*i.e.*, flow of unconverted mixing gas being nine times higher than the plasma effluent) is used in all conditions, which results in *n*_mix_ = 2.25 × 10^20^ cm^−3^, equal to nine times the initial number density at 293.15 K and 1 atm. Because the mixing is given by an exponential function, it only tends to zero asymptotically (never actually becoming zero). For reference, 99% of the mixing has occurred at 4.61, 46.1 and 461 ms in the afterglow for the characteristic times of 1, 10 and 100 ms.2
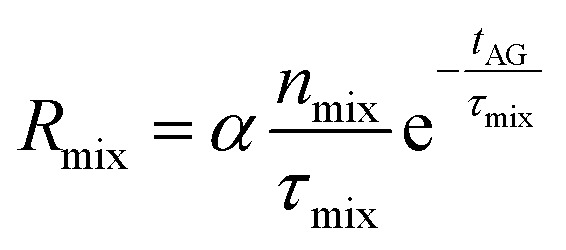
The equation for *R*_mix_ can be associated with diffusion in a parallel-plate geometry, in which the mixing time (*τ*_mix_) is expressed using a diffusion coefficient (*D*) and a length (*L*), 
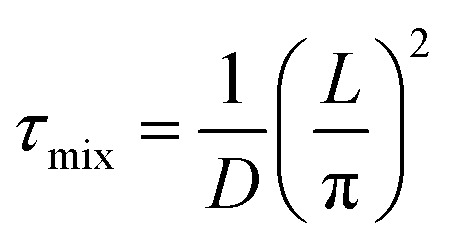
.^27,28^ However, due to the 0D nature of our model, these *D* and *L* parameters do not have a physical meaning. In the interest of simplifying the analysis, we directly specify the *τ*_mix_ values, rather than having them defined by a combination of *D* and *L* values. Therefore, the *τ*_mix_ values used in this study are not intended to be correlated to diffusion, instead they are selected to emulate cooling rates consistent with those observed in the other quenching methods since the focus of our study is on the effect of the cooling rate on the kinetics.

All simulations are conducted at a constant pressure of 1 atmosphere, and this is directly linked to the absolute number densities for each species in the model. There are two factors that influence the total number density in the system: temperature and chemical reactions. As chemical reactions take place, and the gas temperature changes, the gas needs to expand or contract accordingly if the number density changes, to maintain a constant pressure. This is achieved with the *R*_expansion_ term in eqn (1), which is further explained in the ESI (Section S1).†

The gas expansion is monitored throughout the simulation using eqn (3), which represents the ratio of the mass density (*ρ*) at the start of the simulation and the end. The mass density is the summation of the products of the number density (*n*_*j*_) and mass (*m*_*j*_) of each species (*j*) in the model.3
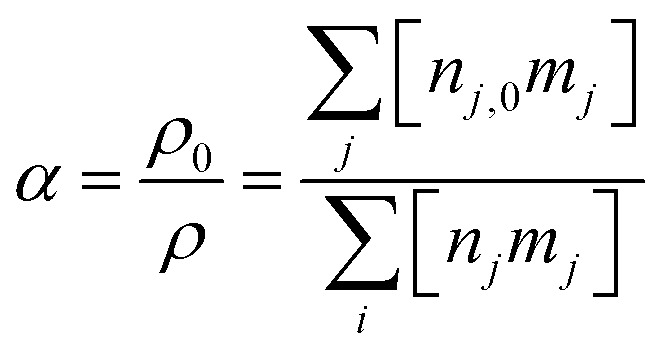


While the temperature is kept constant in the plasma portion of the simulation, in the post-plasma portion the heat balance equation is solved to calculate the temperature self-consistently. ZDPlasKin normally considers a system at constant volume by using the ratio of specific heats to describe the isochoric heat capacity. However, in this model we consider a system at constant pressure, and therefore the isobaric heat capacity is included instead, thus accounting for volumetric expansion. This heat balance equation is given in eqn (4), in which *R*_gas_ is the universal gas constant, *c*_p,mix_ is the heat capacity of the mixture (see ESI, Section S1 and eqn (S3)†), d*T*/d*t* represents the change in temperature with respect to time, *Q*_reaction_ is the heat gained or lost as a result of reactions, while *Q*_conductive_ and *Q*_mixing_ represent the conductive heat losses and the heat losses resulting from post-plasma mixing, respectively. When quenching through conductive cooling is investigated, the *Q*_conductive_ term is used, while the *Q*_mixing_ term is set to zero. Conversely, in the case of quenching through mixing, the *Q*_mixing_ term is used and the *Q*_conductive_ term is zero.4
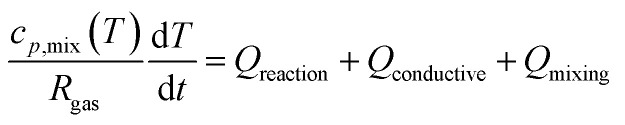


The heat exchange due to reactions (*Q*_reaction_) is calculated using eqn (5), with *N*_g_ the total number density of the heavy species in the simulation, *R*_*i*_ the reaction rate of reaction *i* and *ε*_*i*_ the reaction enthalpy of reaction *i* (see ESI, Section S1 and eqn (S4)†).5
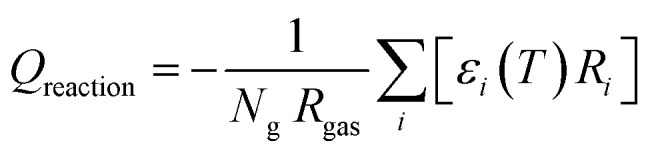


The conductive heat loss is calculated using eqn (6), in which *T* is the gas temperature, *T*_0_ is the reference wall temperature (293.15 K), *k*_B_ the Boltzmann constant, *r* the radius of the plasma (chosen as 1 cm), *λ*_mix_ the thermal conductivity of the gas mixture (see ESI, Section S1 and eqn (S5)†) and *c* an additional factor to artificially increase the external cooling (varied between 1, 10 and 100). This additional *c*-factor is similar to the one used by Vermeiren *et al.*^11^ The equation assumes a parabolic temperature profile with *T* being the radially average temperature and *T*_0_ the temperature at the wall.^29^ This provides a basic approximation of the temperature in the afterglow. The exact and precise value, however, is not the main focus of our study, as the most important effect here is the cooling rate on the kinetics. For the simulations that investigate post-plasma mixing, this conductive cooling term is set to zero. This is done to emulate a perfectly insulated system, which will isolate the effects of mixing by eliminating competition with conductive cooling. This ensures that the gas temperature remains at high values for a longer time, thus creating the optimal conditions for conversion of the freshly mixed gas.6
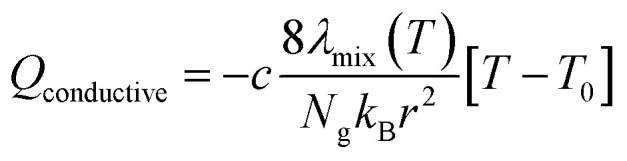


Finally, when applying post-plasma mixing, additional species are added to the afterglow without the removal of other species, which effectively increases the size of the system. Besides, since these new species do not have the same temperature, an amount of energy is required to equalise the temperature of these species to the rest of the system, which affects the heat balance. This is accounted for in the mixing heat term (*Q*_mixing_) defined by eqn (7), in which *R*_mix_ is the mixing rate, *x*_mix,*s*_ is the fraction of species *s* in the mixed gas (both explained above already), *H*^f^_*s*_ is the temperature-dependent enthalpy of species *s* (obtained from McBride *et al.*^30^ and Burcat *et al.*^31^), *T* is the gas temperature and *T*_0_ is the temperature of the mixing gas (293.15 K). In the first set of conditions, where enhanced conductive cooling is used to quench the afterglow, no mixing is used and therefore this *Q*_mixing_ term is zero.7
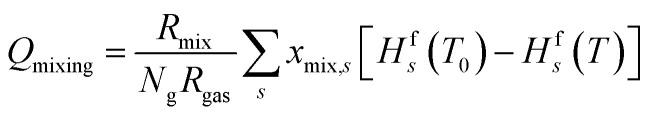


The metrics of conversion, yield and selectivity of various species are presented for different simulated conditions, following the recommendations of Wanten, *et al.*^32^ The conversion of the initial gases CO_2_ and CH_4_ is calculated using eqn (8), where *n*^in^_*s*_ and *n*^out^_*s*_ are the number density of species *s* (CO_2_ or CH_4_) entering and leaving the simulations, and *α* is the gas expansion factor. For the simulations where mixing was considered, the initial number density includes both the original and the additional gas densities.8
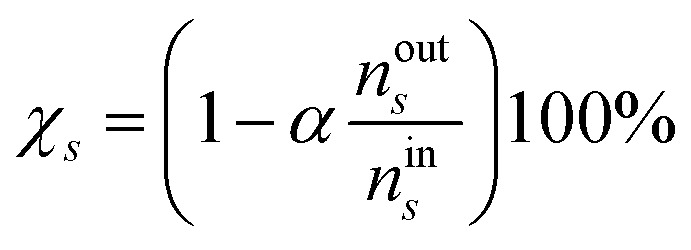


In the Results and discussion section, we plot the selectivity of the product species as a function of the plasma temperature, to show its effects on the product distribution. The selectivity is calculated using eqn (9), where A is the base-atom (C, H or O in this case) and *μ*^A^_*s*_ and *μ*^A^_*i*_ are the numbers of the base-atom in product *s* and reactants *i*, respectively.^32^ The base-atom is required to differentiate between the number of each atom type in the gas mixture, which eventually become the products. For example, the selectivity of CO can be calculated relative to the amount of C (from CO_2_ and CH_4_) or O (from CO_2_) present in the simulations. Therefore, multiple selectivity values can be calculated for one product. By definition, the sum of all selectivity values must be 100%, which is the case for each base-atom.^32^ To simplify the understandability and presentation of the results, we display only one value for each product, prioritising the base-atoms in the following order: first carbon (C), then hydrogen (H), and finally oxygen (O). In some cases, product selectivities for different base-atoms are shown in the same figure, thus it is important to keep in mind that the sum of the selectivity can be above 100%. Once again, the results will focus on the individual products and general trends, but not the total sum. This strategy is commonly used in the case of simulations with mixing, to present changes in product distribution regardless of the achieved conversion.^32^9
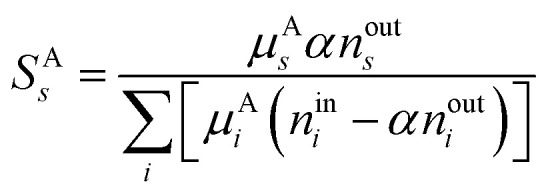


Besides conversion and selectivity, another important evaluated metric is the energy cost of the process. To this end, we calculated energy cost of conversion, as the ratio between the energy input and the obtained conversion. In practice, the lower the energy cost, the more energy efficient the process is. In our case, because we set a constant plasma temperature, no energy input is defined. Therefore, we utilise a minimum energy input (MEI), which is defined as the enthalpy difference between the initial state (CO_2_ and CH_4_ at 293.15 K) and the state in the plasma (at the fixed temperature). This represents the minimum possible energy required to obtain this state. It is calculated using eqn (10), in which *H*^f^_*s*_ is the formation enthalpy of species *s*, *n*_*s*_ is the species density, *α* is the gas expansion factor and *N*_A_ is Avogadro's constant. The formation enthalpy is determined from the thermodynamic data from McBride *et al.*^30^ and Burcat *et al.*^31^ We use this MEI concept to define the minimum energy cost of conversion (MEC) (eqn (11)), which represents the minimum energy cost achievable for these conditions. *χ*_tot_ is the weighted average of the CO_2_ and CH_4_ conversion relative to their initial concentrations. Even though this cannot be compared to experimental data (as the calculations do not consider energy loss processes), this parameter allows us to compare between our different operating conditions and evaluate their potential.10
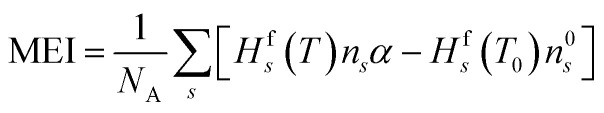
11
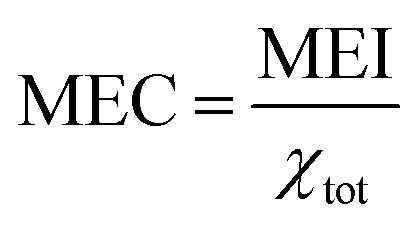


For the simulations with post-plasma mixing, we also calculate the additional conversion (*χ*_additional_), which represents the relative increase in conversion between the end of the plasma and the end of the post-plasma region. This is determined using eqn (12), in which *χ*_end_ and *χ*_plasma_ are the conversion at the end of the simulation and at the end of the plasma region, respectively, and *D* is the dilution degree from mixing (10%, as explained above). Note that when the conversion does not change in the afterglow, *χ*_additional_ will be 0%; and in the case of recombination, *χ*_additional_ will be negative. Finally, this equation does not distinguish between further conversion of gas treated by the plasma or conversion of mixed gas in the hot afterglow.12
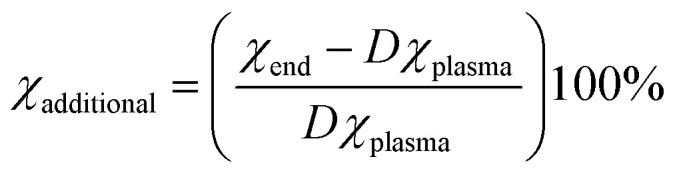


## 3. Results & discussion

### 3.1. Post-plasma cooling

We first analyse the results from simulations where conductive cooling is applied post-plasma. The extent of cooling (and thus quenching) is modulated by the factor *c*, as described in the previous section. For the three investigated CO_2_/CH_4_ ratios (30/70, 50/50, 70/30) and the three different *c*-factors (1, 10, 100), the resulting gas cooling can be observed in the temperature profiles shown in Fig. S1–S3 in ESI (Section S3).† In turn, this gives rise to cooling rates in the order of 10^5^–10^8^ K s^−1^ (see panels b, d and f in the abovementioned figures) at the start of the afterglow. This magnitude of cooling rate has been proven to be beneficial for the conversion of CO_2_,^17,18^ which bodes well for this theoretical study of DRM.

The differences observed in the temperature profiles across the three CO_2_/CH_4_ ratios are ascribed to the lesser and greater proportion of CO_2_ or CH_4_ in the mixture, which affects the thermal conductivity and heat capacity (leading to changes in the temperature profiles). When slower cooling is considered (*c*-factor = 1), exothermic radical recombination occurs at high temperature, rendering undisturbed temperature profiles and resulting in a smooth decrease. In the case of faster conductive cooling (*i.e.*, larger *c*-factors), these exothermic reactions are forced to occur at lower temperatures, deaccelerating the temperature drop at certain points in the afterglow. We do not focus on these absolute temperature profiles in the afterglow for comparing the kinetics, conversion or selectivity results, instead we focus on the overall trends, which provide a more qualitative comparison. Higher-dimensional fluid models would offer a more realistic and detailed simulation of these plasma systems, enabling more quantitative and space-resolved insights into the post-plasma effects. However, these models also introduce significant complexity and tend to be more specific to particular setups or reactor geometries. This lies beyond the scope of this work, as our focus is on gaining in-depth knowledge on the overall kinetics, hence we consider our simpler and more universal modelling approach better suited for this work. Note that for plasma temperatures near 2000 K, a steady state has not yet been fully reached within the simulated plasma residence time. Therefore, unreacted CO_2_ and CH_4_ and intermediate species from incomplete conversion can still be present.

#### 3.1.1. 50/50 ratio CO_2_/CH_4_

**Fig. 2:**
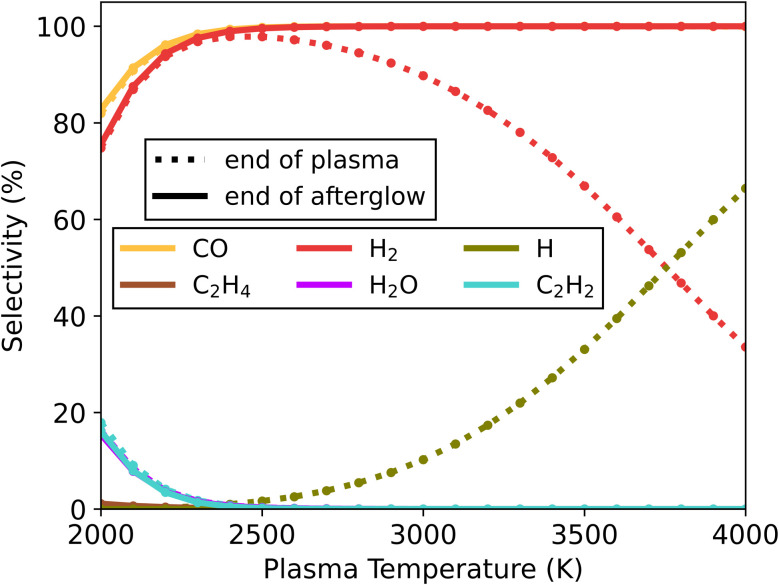
Selectivity of the main species (above 5%) as a function of the plasma temperature, for the 50/50 CO_2_/CH_4_ ratio and *c*-factor of 1 (no enhanced cooling), at the end of the plasma zone (point A in Fig. 1; dotted lines) and end of the afterglow (point B in Fig. 1; solid lines). The CO curves (both at the end of the plasma and afterglow) and H_2_ curves at the end of the afterglow largely overlap. Also, the H_2_O and C_2_H_2_ selectivity curves overlap both at the end of the plasma and afterglow. Near full conversion of CO_2_ and CH_4_ is observed under all conditions, at the end of the plasma, and maintained till the end of the afterglow.

The results from simulations with 50/50 CO_2_/CH_4_ mixtures and *c* = 1 are shown in Fig. 2. For low plasma temperatures, small fractions of C_2_H_2_ and H_2_O are present, with the highest selectivity registered at 2000 K – 15 and 16%, respectively. At this temperature the conversion of CH_4_ and CO_2_ reaches 93 and 96%, respectively. At higher plasma temperatures, the simulations reach a steady state in the plasma and the selectivity towards C_2_H_2_ and H_2_O is lower, with less than 1% above 2400 K. As the plasma temperature is increased, more H radicals are found in the plasma, with a selectivity of 66% at 4000 K. However, in the afterglow these radicals recombine exclusively to H_2_, which occurs through a two-reaction pathway involving CO, according to our kinetics scheme. Our simulations show that the measured conversion for this mixture is preserved in the post-plasma region, with syngas ratio (*i.e.*, H_2_/CO) of 1, which corresponds to the theoretical product distribution from the DRM reaction.

When the cooling degree is increased by a ten- or hundred-fold (*c* = 10, 100), we observe the same behaviour (see Fig. S4†), with only negligible alterations in minor product species (H_2_O, C_2_H_2_ and CH_4_). In summary, our model suggests that for a 50/50 CO_2_/CH_4_ mixture, quenching is not required to maintain the conversion reached in the afterglow, while the main products are consistently CO and H_2_.

#### 3.1.2. 30/70 ratio CO_2_/CH_4_

**Fig. 3:**
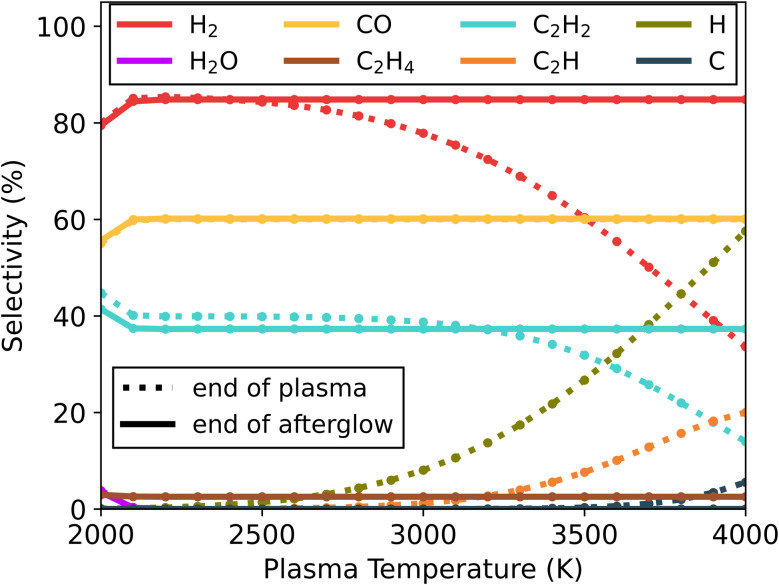
Selectivity of the main species (above 5%) as a function of the plasma temperature, for the 30/70 CO_2_/CH_4_ ratio and *c*-factor of 1 (no enhanced cooling), at the end of the plasma zone (point A in Fig. 1; dotted lines) and end of the afterglow (point B in Fig. 1; solid lines). Full conversion of CO_2_ and CH_4_ is observed under all conditions (except for 2000 K, as explained in the text).

For the gas mixture with an excess of CH_4_ (30/70) (Fig. 3), both CO_2_ and CH_4_ are fully dissociated in the plasma region, with conversion above 99% for both gases. This is the case for all plasma temperatures, except 2000 K where the conversion process is not fully completed, with CH_4_ and CO_2_ conversion reaching 97 and 98%, respectively. Also, at 2000 K a H_2_O selectivity of 3.5% is observed (from the incomplete conversion process), though this falls below 0.4% for all higher temperatures. Similar to the previous mixture (50/50), the presence of H radicals at the end of the plasma is clearly identifiable. However, with an excess of CH_4_, also C_2_H radicals are formed with a maximum concentration of 20% at 4000 K (see Fig. 3), and to a lesser extent, C radicals with a maximum of 5.5% at 4000 K (see Fig. 3). The recombination of these radicals in the afterglow forms a large concentration of H_2_ (85%) and C_2_H_2_ (37%) seen in Fig. 3, and small fractions of C_2_H_4_ (2.5%), which is displayed in Fig. S5.† Overall, the temperature in the plasma has a negligible effect on the final product distribution. It should also be noted that for this gas mixture the resulting syngas ratio is 2, which is ideal for further Fischer–Tropsch processing or methanol synthesis.^33^

When the cooling rate for this mixture is increased, the main products predicted by the model continue to be H_2_, CO and C_2_H_2_, without any new products being formed (see Fig. S5†). However, the C_2_H_4_ selectivity is reduced to 1.3% for both *c*-factors 10 and 100 (compared to 2.5% at *c* = 1). In conclusion, for 30/70 CO_2_/CH_4_ mixtures, these results also reveal that quenching of the afterglow is not beneficial for conversion and has a negligible effect on the species distribution.

#### 3.1.3. 70/30 ratio CO_2_/CH_4_

**Fig. 4:**
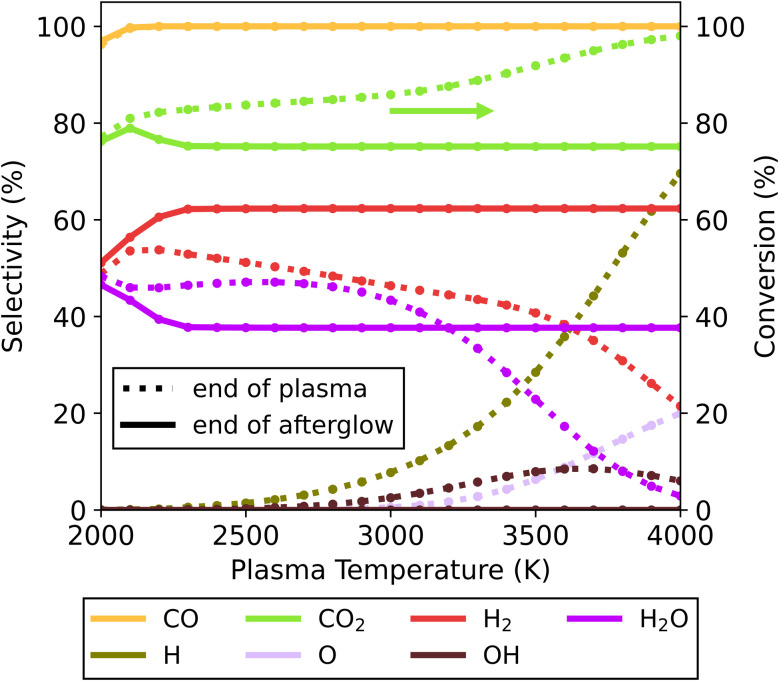
Selectivity of the main species (above 5%) as a function of the plasma temperature, for the 30/70 CO_2_/CH_4_ ratio and *c*-factor of 1 (no enhance cooling), at the end of the plasma zone (point A in Fig. 1; dotted lines) and end of the afterglow (point B in Fig. 1; solid lines). Full conversion of CH_4_ is observed under all conditions, but not for CO_2_, and therefore the CO_2_ conversion is also plotted (right *y*-axis).

Lastly, we consider a CO_2_/CH_4_ mixture at a 70/30 ratio (with excess CO_2_). Akin to the previously discussed mixtures, the afterglow has a negligible effect in the lower end of the plasma temperature range (<2300 K, see Fig. 4). At these temperatures, the concentration of radical species is insignificant, resulting in unobservable effects from recombination reactions in the afterglow. On the other hand, more interesting effects are observed at higher plasma temperatures. Despite reaching a steady state, complete conversion of CO_2_ in the plasma zone is not achieved at a 70/30 CO_2_/CH_4_ ratio, which is an important factor to consider in this case. Also, various radical species (such as H, OH and O) are present at the end of the plasma zone, along with the primary products: H_2_, CO and H_2_O. As a result, we encounter more complex kinetics in this afterglow compared to the two previous mixtures.

In the lower range of plasma temperatures (<3000 K), radical concentrations are rather low (only small fractions of H and OH are present). Despite radical recombination being limited, there is a notable shift in product distribution – with the formation of H_2_ and CO_2_ being favoured over that of CO and H_2_O, through the occurrence of the water gas shift (WGS) reaction (R2). For instance, at 2500 K the conversion of CO_2_ decreases from 84 to 75% due to WGS, while simultaneously the H_2_ selectivity increases from 51 to 62% and the H_2_O selectivity decreases from 47 to 38%.

R2 CO + H_2_O → CO_2_ + H_2_

For higher plasma temperatures, higher CO_2_ conversions can be achieved in the plasma, up to 98% at 4000 K. This is accompanied by considerable formation of H, OH and O radicals, instead of H_2_O and H_2_. At this temperature, the low H_2_O concentrations limit the occurrence of WGS. However, between 2300 and 4000 K, we observe approximately the same product distribution at the end of the afterglow, regardless of the initial plasma temperature, which is further explained in the next paragraph. Also noteworthy is that all extra CO_2_ conversion originating from the higher plasma temperatures is lost again in the afterglow upon gas cooling. This negates the supposed benefits of high plasma temperatures for CO_2_ conversion, as in this case this effect alone is counteracted in the afterglow region. This is aligned with results for pure CO_2_ conversion without quenching of Vermeiren *et al.*^11^ where significant recombination is demonstrated to reduce the conversion to similar levels, regardless of the gas temperature obtained in the plasma.

**Fig. 5:**
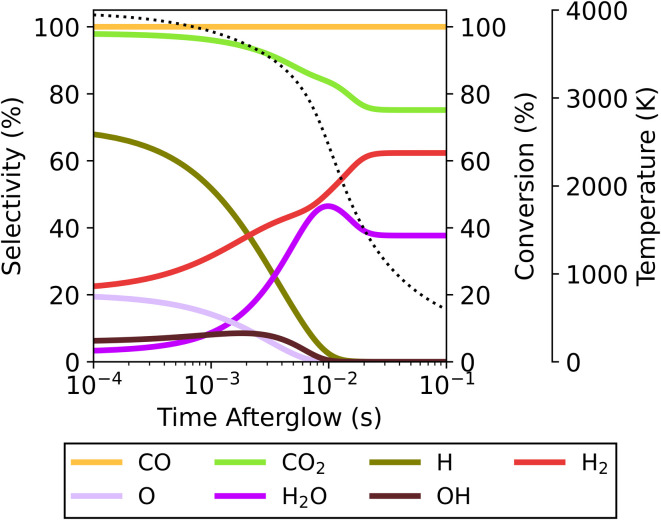
Evolution of the selectivity of the main species in the afterglow, starting from a plasma temperature of 4000 K for the 70/30 CO_2_/CH_4_ ratio and *c*-factor of 1. The evolution of the CO_2_ conversion and of the gas temperature (dotted line) are also plotted, and shown on the right axis.

These recombination reactions and pathways are further explored by analysing the evolution of key species over time in the afterglow for the 4000 K plasma temperature case (Fig. 5 below). Owing to the presence of O, OH and H radicals produced in the plasma for the 70/30 CO_2_/CH_4_ ratio, multiple recombination processes can occur. The two most straightforward processes are the O + CO reaction to CO_2_ and H + H to H_2_. Aside from these, a reaction of minor importance occurs between O and H to form OH, with the OH selectivity peaking at 8.5% around 1.8 ms. Subsequently, these OH radicals are important to further produce H_2_O, which has a maximum selectivity of 46% at 9.9 ms. At this point the system has reached a state similar to the equilibrium composition at around 2500 K (also obtained at the end of the plasma for plasma temperatures of 2000–3000 K, *cf.*Fig. 4). Since the kinetics up to this point significantly outpaces the cooling rate, differences in cooling have minimal impact. The temperature is still sufficient to allow further reactions, and the WGS reaction reduces the H_2_O selectivity again before it plateaus at 38%. This is the result of the kinetics eventually slowing down, effectively halting all reactions and reaching the final steady state. Because this part of the temperature profile is similar, regardless of the starting plasma temperature (Fig. S12 in ESI†) the overall chemical changes are also similar, resulting in overall similar product distributions across the entire temperature range. The CO selectivity is constant at 100%, and as this is the only main C-containing species, the C-based selectivity remains constant. However, the absolute amount of CO does decrease (not shown in the figure) as the CO_2_ conversion drops due to WGS.

**Fig. 6:**
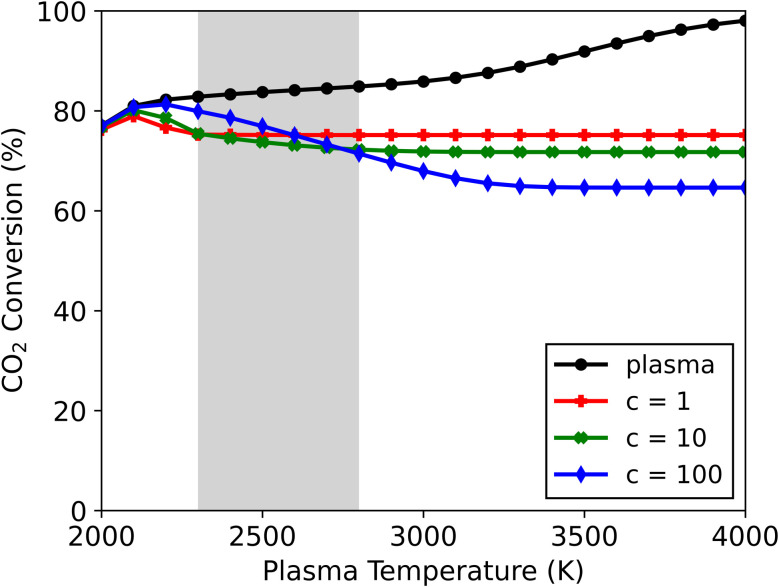
CO_2_ conversion in the plasma and in the afterglow (for three different *c*-factors: 1, 10 and 100) as a function of the plasma temperature, for the CO_2_/CH_4_ ratio of 70/30. The range of plasma temperatures where the transition between the two afterglow effects occurs is indicated with a grey rectangle.

Upon increasing the cooling factor to 10 and 100, radical recombination towards CO_2_ continues to be observed in the afterglow in all cases for this mixture (see Fig. S6†). The lower end of the plasma temperature range (*T* < 2100 K) remains largely unaffected by the magnitude of cooling. This is also where the highest CO_2_ conversions are attained upon cooling implementation (see Fig. 6) – with 79, 80 and 81% conversion for *c*-factors of 1, 10 and 100, and at 2100, 2100 and 2200 K, respectively. These conversions lie slightly below those found in the plasma, signalling only a small amount of CO + O recombination. This can be attributed to the small amount of radicals present, combined with the relatively low plasma temperatures, which upon quenching in the afterglow will drastically limit recombination reactions. Therefore, at these temperatures the effect of the WGS reaction is small, which in turn preserves the higher CO_2_ conversions obtained in the plasma (alongside the CO and H_2_O products).

At plasma temperatures exceeding 2800 K, the opposite effect is observed. A shift in the afterglow reaction mechanisms promotes the formation of CO_2_ and H_2_ over that of H_2_O. As shown in Fig. 6, the CO_2_ conversion drops with increasing cooling, only reaching 72 and 65% (for *c*-factors of 10 and 100, respectively) at a plasma temperature of 4000 K, compared to 75% for *c* = 1. Consequently, this allows for more H_2_ to be produced – with a selectivity of 66 and 74% for *c*-factors of 10 and 100, respectively, compared to 62% for *c* = 1 (see Fig. S6†). This enhanced H_2_ formation, combined with lower CO_2_ conversions, improves the syngas ratio, from 0.45 to 0.59. The product selectivities and chemical pathways are consistent with those observed at a *c*-factor of 1 (Fig. 5). However, the faster decrease in temperature in the afterglow (at *c* = 10 and *c* = 100) forces radical recombination to occur predominantly at lower temperatures (see Fig. S7†), which favours the recombination of O with CO to CO_2_, over the reaction with H to OH, which subsequently forms H_2_O. In this case, the rapid temperature drop slows the recombination reactions of the H radicals which results in an overlap between the radical recombination and WGS reaction steps. A part of the H radicals remains as free radicals even at lower temperatures (>1000 K, where for the slower cooling all reactions have ceased around 1500 K). The presence of these radicals keeps the system reactive at these lower temperatures which changes the pathways and the final steady state the system reaches. The more H remains as radicals rather than reacting to H_2_O in the first stage, the higher the amount of H_2_ (and CO_2_) in the final product distribution. Because of this, the system evolves towards a different steady state compared to the slower cooling in Fig. 5. For plasma temperatures between 2300 and 2800 K, there is effectively a transition zone (see Fig. 6), where these opposing effects (faster cooling limiting the WGS reaction and the shift in radical combination pathways) compete. In this zone, the overall CO_2_ conversion is dependent on the combined influences of cooling and plasma temperature.

In conclusion, the mixture with excess CO_2_ (70/30) clearly exhibits distinct behaviour compared to the other mixtures (50/50 and 30/70), as the CO_2_ conversion is shown to decrease drastically in the afterglow upon cooling. The drop in conversion is worsened by increasing the cooling rate to quench the hot gas in the afterglow. The lower CO_2_ conversions coincide with changes in product distribution, with CO and H_2_O being favoured at the lower end of the plasma temperature range, while CO_2_ and H_2_ are dominant at the higher end.

The latter effect is similar to the observations noted by Kwon *et al.*^20^ from their quenching rod experiment. They also reported higher selectivity towards H_2_ (instead of H_2_O) alongside a lower CO_2_ conversion. Even though they attributed this result to the suppression of RWGS (*i.e.*, further limiting conversion of CO_2_ with H_2_ in the afterglow), our model suggests a different mechanism could be responsible for the observed shift. Their experiments presumably have plasma temperatures above 2800 K, where the CO_2_ conversion decreases as a result of quenching the post-plasma region (as seen in Fig. 6). Under these conditions, our calculations show significant radical recombination towards CO_2_ regardless of quenching, however by accelerating the temperature drop (*i.e.*, stronger cooling) different species are favoured, as illustrated in Fig. 5 and S7.† While we trust these modelled results, we are aware that other experimental factors (which cannot be captured by our 0D model) may influence the reaction kinetics.

Targeting this effect to synthesise higher H_2_ concentrations (over H_2_O) at higher plasma temperatures is certainly beneficial, as H_2_O is an unwanted side product. A H_2_-richer product mixture improves the overall value of the effluent. The ensuing lower CO_2_ conversion is an unfortunate side effect, but not a major issue as the remaining CO_2_ requires post-processing in a separation step in either case (as complete conversion cannot be achieved). However, a detailed process optimisation study and an in-depth economic analysis are necessary to determine the more cost-effective targets.

Another important consideration is the role of the afterglow in further converting CO_2_ and CH_4_ when a steady state is not achieved in the plasma (due to a shorter residence time, for example). This is a plausible scenario for the lower end of the temperature range, in which case quenching of the hot gas can suppress further dissociations in the afterglow, also lowering the conversion.

#### 3.1.4. Effect on energy cost

While the primary focus of this work lies in the chemical aspects, we have also evaluated the energy costs required to achieve the studied conditions and provide additional insights into the effect of the afterglow on the energy cost of DRM, hereby shedding light on the nonlinear relationship between these conditions and the input energy. We calculated the difference in enthalpy between the initial system (at the start of the simulation, *i.e.*, a mixture of CO_2_ and CH_4_ at 293.15 K) and at the end of the plasma zone (mixture of unconverted CO_2_ and CH_4_, as well as products and radical species at the plasma temperature), calculated using eqn (10). This enthalpy difference represents the minimum energy required to drive the system to the final chemical state (at the end of the plasma), which includes the chemical changes, as well as the temperature increase that occurs in the plasma. Note that this calculation does not include thermal losses; therefore, these results cannot be directly compared to experimental data. However, they give an indication of the minimum values of an idealised system.

The energy input (see Fig. S8†) ranges between 7.5 and 37 kJ L^−1^ depending on gas mixture (CO_2_/CH_4_ ratio) and plasma temperatures, *i.e.*, higher temperature values correspond to higher energy inputs, since more energy is required to reach higher temperatures, leading to greater dissociation into radicals. From this minimum energy input (eqn (10)), we calculated the minimum energy cost for CO_2_ and CH_4_ conversion (eqn (11)), based on the total conversion reached at the end of the afterglow for the different cooling strengths (*c*-factors) (see Fig. S9†). This energy cost is approximately equal to the energy input, which is logical as the conversion of both CO_2_ and CH_4_ in the plasma zone is approximately 100% in all cases. The exception is the 70/30 CO_2_/CH_4_ ratio (where the CO_2_ conversion is lower than 100%, as shown in Fig. 6) which has a total conversion between 75 and 87% depending on plasma temperature and cooling strength. In this specific case, the minimum energy cost increases with the cooling strength, however, the overall difference is less than 3.4 kJ L^−1^.

Our results clearly suggest it is best to maintain a plasma temperature as low as possible (while still being sufficiently high to fully convert the reactants) to obtain the lowest minimum energy costs. Also, an interesting analysis is the comparison of our results to the target energy cost of 4.27 eV per molecule (17.1 kJ L^−1^), which was proposed by Snoeckx and Bogaerts^6^ for competitiveness with existing technologies. This would suggest that plasma temperatures above 3000 K should be avoided, as such systems could not meet this energy target, while temperatures below 3000 K could meet this target.

However, it must be noted that these insights should be nuanced when comparing this idealised system to experimental conditions. Firstly, our calculations only consider the minimum energy input, and not the total energy input of the process or reactor setup, which in reality is higher since energy loss channels are present (such as heat loss from the plasma) and the efficiency of the power supply is not 100%. Under experimental conditions, these factors will contribute to a higher energy cost. Secondly, this approach only accounts for the gas that is interacting with the plasma, *i.e.*, it assumes that 100% of the gas flow is treated by the plasma. However, experimentally, the power deposition in the reactor is localised and non-uniform, which results in only a fraction of the gas flow to be treated by the plasma. This also results in a temperature gradient across the plasma, of which parts can operate at more ideal conditions with respect to energy cost. On the other hand, this could also create regions with less ideal conditions, by either operating at a too high temperature (above 3000 K, as discussed above) or too low to achieve considerable conversion at the periphery of the plasma, and both effects would increase the energy cost again. Ultimately, the above experimental intricacies will probably lead to a higher energy input requirement, which according to our results will not be reflected in an enhanced overall conversion; on the contrary, it will probably result in an increased energy cost.

### 3.2. Post-plasma mixing

While for the above study of quenching *via* fast cooling, we assumed all gas passes through the plasma discharge, this is unlikely since experimental setups are not completely and homogeneously filled with plasma.^34–36^ Instead, what is most likely is the existence of a peripheral colder zone surrounding the plasma, where reactant conversion is significantly lower. When these two zones remain separated, the results of the previous section apply specifically to the plasma and its effluent only, albeit the overall conversion will be significantly lower than those predicted by the model. On the other hand, this colder surrounding gas flow can mix with the plasma effluent in the hot afterglow, which will lead to additional thermal conversion, improving the overall output of the reactor. This effect is targeted in some reactors by introducing a nozzle to force these two distinct layers of gas to mix.^14,15,17^

In this section, we apply our model to explore this effect theoretically, by adding unconverted cold gas in the hot afterglow of a DRM plasma. To this end, we consider a perfectly insulated system, which makes gas mixing the only factor influencing the temperature. This represents the ideal and best-case scenario to target maximum additional conversion of the added gas. The plasma effluent is diluted to 10%, which corresponds to adding nine times the amount of initial gas during the afterglow region. As this dilution lowers the temperature to below 1000 K (at the end of the mixing stage), thermal reactions are effectively halted. We investigate three different mixing rates, modulated through the mixing time (*τ*_mix_) set to 1, 10 or 100 ms. Further explanation regarding the implementation of the mixing is given in the model description (Section 2.3). The plasma zone assumed before the mixing has been described in the previous section, with temperatures ranging from 2000 to 4000 K and three distinct CO_2_/CH_4_ ratios.

An overview of the temperatures and cooling rates throughout the afterglow for different gas mixtures, plasma temperatures and characteristic mixing times, is shown in Fig. S10–S12 in the ESI,† demonstrating that the highest cooling rates vary between 10^5^ and 10^8^ K s^−1^, depending on the specific conditions. This is a similar range to that observed in the previous section.

#### 3.2.1. Additional conversion

**Fig. 7:**
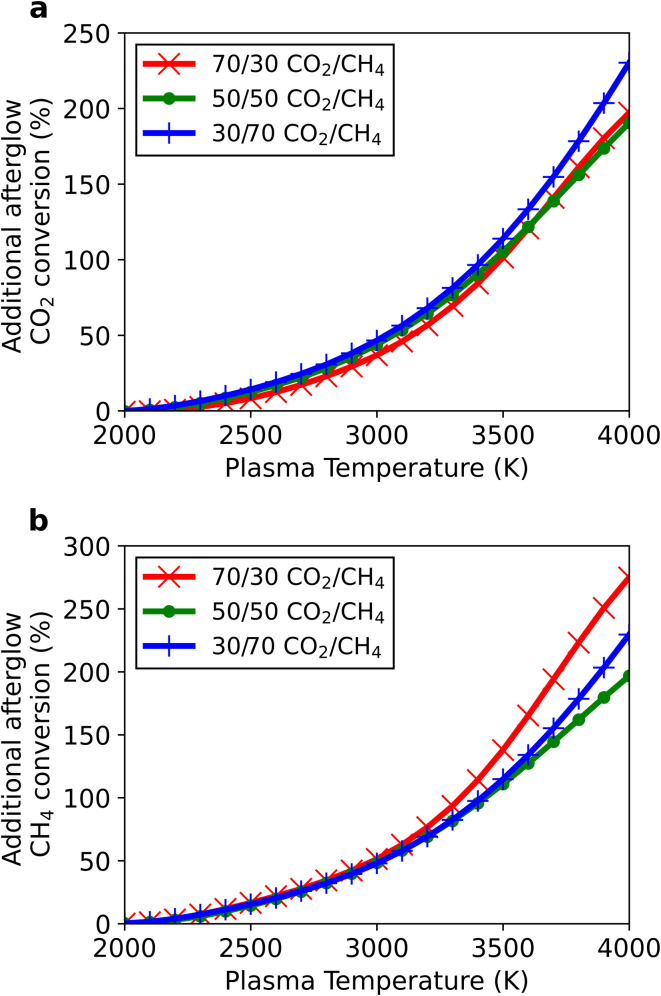
Additional CO_2_ (a) and CH_4_ (b) conversion obtained in the afterglow relative to the conversion obtained in the plasma, as a function of plasma temperature, for three different CO_2_/CH_4_ ratios (70/30, 50/50, 30/70) at *τ*_mix_ = 10 ms.

In this section we compare the conversions obtained in the post-plasma region for a characteristic mixing time of 10 ms (Fig. 7), calculated as the relative increase in conversion between the end of the plasma and the final conversion at the end of the afterglow (accounting for the dilution effect as shown in eqn (12)). The absolute values of CO_2_ and CH_4_ conversion obtained at the end of the plasma, end of the afterglow and the relative additional conversion (as plotted in Fig. 7) are given in Tables S1 and S2 in ESI.†

For the plasma temperature of 2000 K, the overall change in the afterglow is negligible. As the plasma temperature is increased, the extra conversion for both CO_2_ and CH_4_ also rises, with a maximum additional conversion, relative to the conversion in the plasma, of 230% for CO_2_ (at 4000 K and a 30/70 CO_2_/CH_4_ ratio) and 275% for CH_4_ (at 4000 K and a 70/30 ratio). This increasing trend is logical, as the initial higher afterglow temperatures allow the newly added CO_2_ and CH_4_ to experience a longer residence time at elevated temperatures, in turn converting a larger fraction of the mixed gas. In all three gas mixtures, the principal radical in the afterglow is H, which also plays a crucial role in the initial dissociation processes within the plasma. Expectedly, the additional conversion is driven upon reaction of these H radicals with CO_2_ and CH_4_*via* reactions (R3) and (R4), respectively. This can also be correlated to the plasma temperature: as the temperature is raised, higher concentrations of H radicals are available in the afterglow, thereby increasing the conversion.

R3 CO_2_ + H → CO + OH

R4 CH_4_ + H → CH_3_ + H_2_

The most notable effect is seen for the 70/30 CO_2_/CH_4_ ratio, which also undergoes the greatest extent of recombination to CO_2_, decreasing the overall conversion when quenching with fast cooling was considered (see Fig. 6 in Section 3.1.3). This detrimental effect is circumvented with the post-plasma mixing, by shifting the reaction pathways towards dissociation instead of recombination. The increase in CO_2_ density acts to reduce the net recombination reaction of CO and O, and instead CO_2_ conversion is further enhanced by reaction with H radicals (reaction (R3)).

Regarding the other mixing rates (*i.e.*, *τ*_mix_ = 1 and 100 ms), the same trends discussed above are observed, however the additional conversion is closely linked to the mixing rate. At the largest mixing rate (*τ*_mix_ = 1 ms), the system achieves the lowest additional conversion, with maximum values of 202 and 252% for CO_2_ and CH_4_, respectively, while at the lowest mixing rate (*τ*_mix_ = 100 ms), the conversion rises to 258 and 301% for CO_2_ and CH_4_, respectively. These results are again logical and are in line with the explanation given above for the effect of plasma temperature. With stronger mixing, the temperature experiences a faster decrease, thus the reactants have a shorter residence time at sufficiently high temperature to be converted.

These results demonstrate that post-plasma mixing can indeed be beneficial, especially upon coupling of high plasma temperatures with slow mixing. This mixing effect should be nuanced with respect to common experimental conditions, where perfect insulation described in our model is unattainable. As a consequence, heat loss to the reactor walls will increase the overall cooling in the afterglow, thereby diminishing the overall benefit. Nevertheless, the above results provide qualitative insights into how post-plasma mixing can improve the conversion.

#### 3.2.2. Effect on product distribution

While an enhancement in conversion is certainly beneficial, changes in product distribution must also be considered. In this section, we present the selectivity of different products, noting that the selectivity was determined with respect to a base atom (as explained in Section 2.3). Accordingly, carbon has been prioritised over hydrogen and hydrogen over oxygen.

**Fig. 8:**
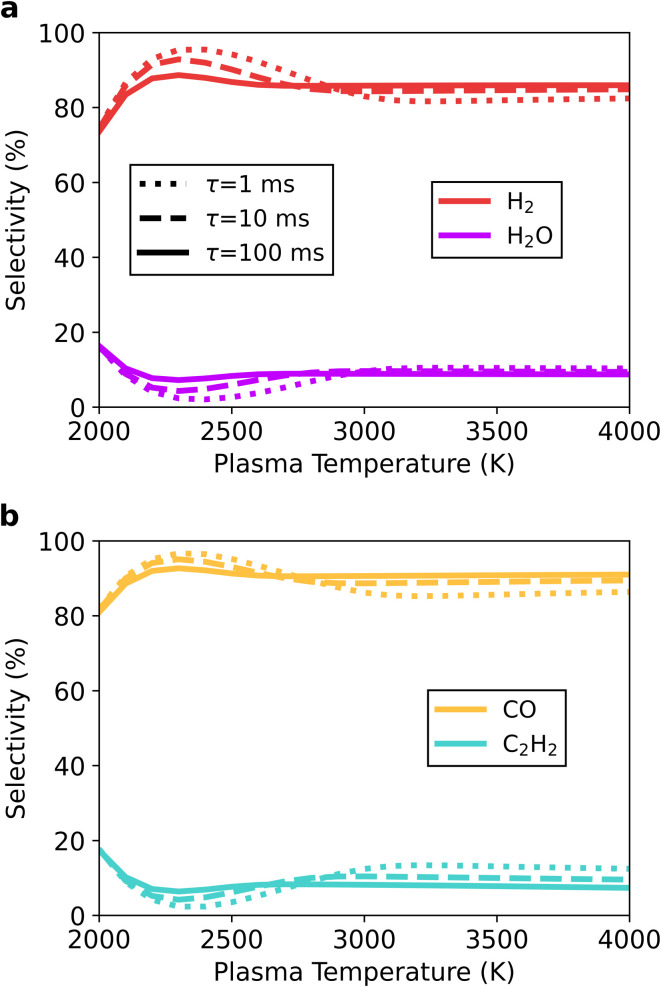
Selectivity towards the main product species as a function of the plasma temperature, for the 50/50 CO_2_/CH_4_ ratio, at the end of the afterglow at *τ*_mix_ = 1 (dotted line), 10 (dashed line) and 100 (solid line) ms. The hydrogen-based H_2_ and H_2_O selectivity is shown in panel a, and the carbon-based CO and C_2_H_2_ selectivity in panel b.

For the stoichiometric CO_2_/CH_4_ ratio of 50/50, syngas is still the main component of the product stream (see Fig. 8). The lowest selectivity is observed at 2000 K and the slowest mixing (*τ*_mix_ = 100 ms), with 74 and 81% for H_2_ and CO, respectively. This can be ascribed to incomplete reactant conversion, forming H_2_O and C_2_H_2_ with 16 and 17% selectivity, respectively. These results are similar to those discussed in the previous section, without post-plasma mixing. The selectivity of the latter species is reduced with increasing plasma temperature, as ‘more complete’ conversion can occur in the plasma. This increases the selectivity towards syngas, reaching a maximum at 2300 K – with H_2_ and CO exhibiting 87 and 93% selectivity, respectively. This corresponds to a C_2_H_2_ and H_2_O selectivity of 6.4 and 7.2%, which again increases for higher plasma temperatures, and this can be explained as follows. Since H_2_O and C_2_H_2_ are intermediate species in the DRM process (occurring at temperatures between 1500 K and 2500 K),^25^ these species can be formed in the post-plasma region of higher plasma temperatures when mixing is implemented. As the plasma temperature drops in the afterglow (due to mixing), it reaches the above-mentioned optimum range for C_2_H_2_ and H_2_O formation, forming these intermediates. However, as the mixing continues, the temperature decreases further, inhibiting the pathways that convert H_2_O and C_2_H_2_ to H_2_ and CO. Hence the former species remain as final products.

The effect of the mixing rate on the product distribution is directly related to the plasma temperature (Fig. 8). For plasma temperatures below 2700 K, increasing the mixing rate favours the formation of syngas. The acceleration of the temperature drop simply results in less influence of the already small additional conversion to H_2_O and C_2_H_2_. Above 3000 K, the opposite effect is seen, with the product selectivity shifting towards H_2_O and C_2_H_2_ (in detriment of syngas). This can be ascribed to the exponential mixing rate, rendering a stronger temperature decrease in the early part of the afterglow (closer to the plasma zone) than that experienced in the later part. As such, the relative contribution of H_2_O and C_2_H_2_ at the tail end of the temperature profile becomes larger, increasing their selectivity.

**Fig. 9:**
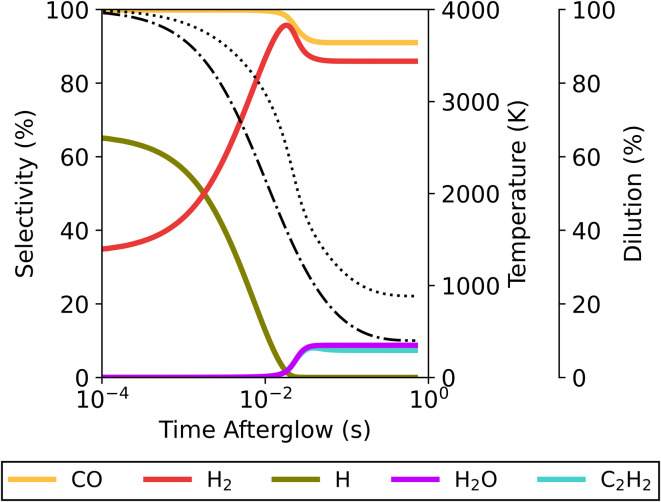
Temporal evolution of the main species' selectivity in the afterglow, starting from a plasma temperature of 4000 K, for the 50/50 CO_2_/CH_4_ ratio at *τ*_mix_ = 100 ms. The temperature (dotted line) and mixing progress (dash-dotted line) are also plotted, and shown on the right axes.

The formation of these products can be further explained by the species selectivity profiles throughout the afterglow in Fig. 9 for the plasma temperature of 4000 K and *τ*_mix_ = 100 ms. In the early afterglow, the remaining H radicals (formed in the plasma zone) are consumed in the direct conversion of the added CO_2_ and CH_4_ to CO and H_2_. As outlined above in reactions (R3) and (R4) (see Section 3.2.1), these H radicals react with both CO_2_ and CH_4_ – being the main driving force for the additional conversion. This also causes a shift in the selectivity from H to H_2_. The secondary product species (C_2_H_2_ and H_2_O) only emerge later in the afterglow, coinciding with a drop in H_2_ and CO selectivity. The selectivity towards H_2_O remains below 1% until 15.9 ms in the afterglow, and at this point the original flow is diluted to 43% and the temperature has dropped from 4000 K to 2675 K. As the temperature decreases further, the H_2_O selectivity increases up to a maximum of 8.7%, while simultaneously C_2_H_2_ is also formed (with 7.4% selectivity). This is when steady state is reached, and the temperature has dropped to approximately 1550 K. Continuing dilution from this point onwards only decreases the gas temperature further, as all reactions are halted; and thus the H_2_O and C_2_H_2_ species will be seen in the final products. For the lower plasma temperatures or faster mixing, the same processes occur, but to a smaller extent due to the reduced residence time in the afterglow, which allows for a lesser extent of chemical reactions.

The work of Sun *et al.*^37^ discusses mixing between the plasma effluent and a surrounding gas stream, using a reactor network model for a microwave plasma setup for DRM with a 1/1 ratio of CO_2_/CH_4_ and compared to their experimental findings. The difference in model description and higher plasma temperature (5000–5900 K) make a direct comparison difficult. However, they reported similar product distributions, mainly syngas production with smaller fractions of H_2_O and C_2_H_2_.

The gas mixtures with different ratios exhibit the same overall effect, with the intermediate species (H_2_O, C_2_H_2_ and C_2_H_4_) emerging as final products because the abrupt temperature drop in the afterglow slows down the kinetics, resulting in incomplete conversion pathways.

**Fig. 10:**
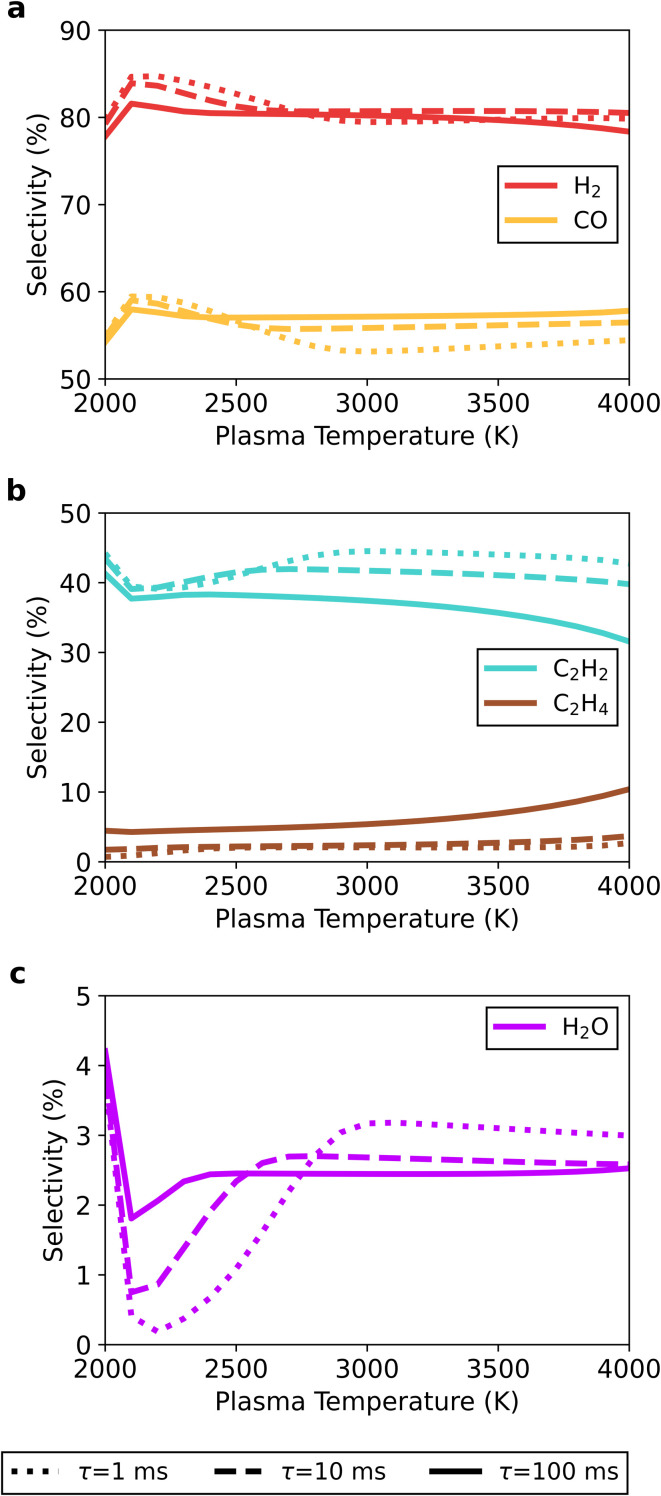
Selectivity at the end of the afterglow towards the main product species (H_2_ and CO in panel a, C_2_H_2_ and C_2_H_4_ in panel b, and H_2_O in panel c) as a function of the plasma temperature, at the 30/70 CO_2_/CH_4_ ratio and *τ*_mix_ = 1 (dotted line), 10 (dashed line) and 100 (solid line) ms.

For the 30/70 CO_2_/CH_4_ ratio (see Fig. 10), the products shift more towards C_2_H_2_ (at the expense of CO), because of the higher CH_4_ fraction compared to the 50/50 ratio. H_2_ is the main product with selectivity between 78 and 85%, while the selectivity of CO is slightly lower (between 54 and 59%), and C_2_H_2_ becomes a significant product – with selectivity between 32 and 44%. The remaining products are H_2_O and C_2_H_4_, with selectivity ranging from 0.19 to 4.2% and from 0.70 to 10%, respectively.

**Fig. 11:**
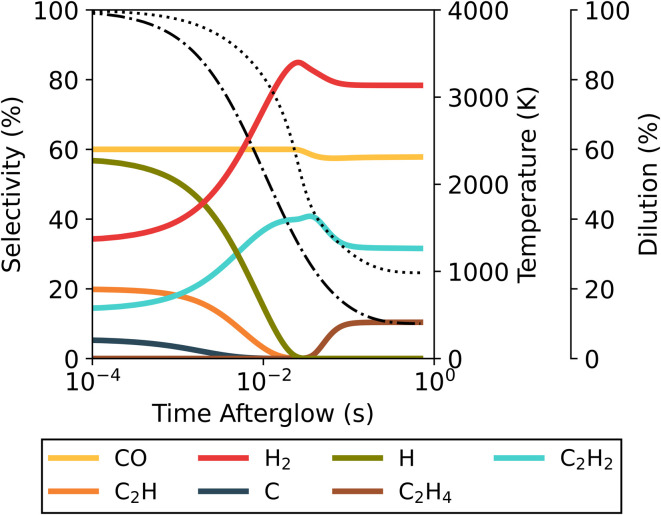
Temporal evolution of the main species' selectivity in the afterglow, starting from a plasma temperature of 4000 K, for the 30/70 CO_2_/CH_4_ ratio at *τ*_mix_ = 100 ms. The temperature (dotted line) and mixing progress (dash-dotted line) are also plotted, and shown on the right axes. The timespan in which the shift from C_2_H_2_ to C_2_H_4_ occurs is indicated with a grey rectangle.

Akin to the 50/50 CO_2_/CH_4_ ratio, the highest selectivity towards H_2_ and CO is observed at 2100 K and faster mixing. However, at the 30/70 CO_2_/CH_4_ ratio, C_2_H_4_ formation does not follow this trend, since it exhibits the highest selectivity at the lowest mixing rate and highest plasma temperature. A transition from C_2_H_2_ to C_2_H_4_ can be noticed in the afterglow when the temperature drops from 1775 to 1230 K for a plasma temperature of 4000 K and *τ*_mix_ = 100 ms (see Fig. 11). This occurs through reactions with H_2_(R5) or with H and CH_4_ with C_2_H_3_ as an intermediate ((R6) and (R7)).

R5 C_2_H_2_ + H_2_(+M) → C_2_H_4_(+M)

R6 C_2_H_2_ + H(+M) → C_2_H_3_(+M)

R7 C_2_H_2_ + CH_4_ → C_2_H_3_ + CH_3_

This transition to C_2_H_4_ proceeds at lower temperatures than the other afterglow processes (*e.g.*, the additional CO_2_ and CH_4_ conversion and the formation of C_2_H_2_ and H_2_O), and therefore much later in the post-plasma region. This can be ascribed to a combination of longer residence times (due to slower mixing) with high C_2_H_2_ concentrations (achieved at high CH_4_ ratios), driving the reactions towards C_2_H_4_. When optimising the process, C_2_H_2_ and especially C_2_H_4_ are worth considering, as they are also valuable products.

Finally, we consider the 70/30 CO_2_/CH_4_ ratio (see Fig. S13†) whose selectivity trends are the least affected by the plasma temperature and mixing speed. The main product is CO, reaching a selectivity between 95 and 100%, while H_2_ and H_2_O range from 49 to 53% and from 45 to 48%, respectively. In this mixture, C_2_H_2_ is formed as a minor product with selectivity between 0.063 and 4.9%. For the lower plasma temperatures (below 2500 K), increasing the mixing speed (accelerating the temperature drop) favours the formation of syngas. However, the product selectivity shifts towards H_2_O and C_2_H_2_ above 2500 K. Again, this can be ascribed to the exponential mixing rate, which causes a stronger temperature decrease in the early afterglow and limits H_2_ and CO formation. In the later afterglow, the relative formation of H_2_O and C_2_H_2_ is increased as the tail end of the temperature profile becomes larger, resulting in higher H_2_O and C_2_H_2_ selectivity.

To summarise, the selectivity results for the three CO_2_/CH_4_ ratios suggest that post-plasma mixing does not yield drastic changes to the product distribution in DRM plasmas. Across the studied range of plasma temperatures and mixing rates, the selectivity of the products varies by less than 10%. The main products across all mixtures are still syngas (H_2_ and CO), with also high fractions of H_2_O or C_2_H_2_ being observed for mixtures with excess CO_2_ or CH_4_, respectively. Additionally, incomplete conversion of the freshly added gas in the afterglow leads to the formation of small quantities of C_2_H_*y*_ and/or H_2_O depending on the gas mixture (C_2_H_2_ and H_2_O for the 50/50 ratio, C_2_H_2_ for excess CO_2_, and H_2_O and C_2_H_4_ for excess CH_4_).

Overall, we would recommend elevated plasma temperatures (4000 K or even higher) combined with slow mixing, to maximize the (additional) CO_2_ and CH_4_ conversion and reach a high syngas yield. Indeed, at elevated plasma temperatures, our results suggest the additional conversion of the mixed gas is directly coupled to a partial selectivity shift from syngas towards secondary products (H_2_O, C_2_H_2_ and C_2_H_4_), with the slowest mixing (*τ*_mix_ = 100 ms) showing the higher syngas selectivity. Despite this, the overall syngas yield is still significantly improved by the additional conversion, which can be industrially more interesting than a slightly higher syngas selectivity. On the other hand, the absolute maximum selectivity towards syngas is obtained for a plasma temperature of ∼2200 K coupled to a fast mixing rate (*τ*_mix_ = 1 ms) of fresh gas in the afterglow. However, the model suggests the effects of both quenching methods are negligible at these conditions.

Considering the non-uniformity of the plasma, there will likely be deviations from an ideal condition. For instance, inevitable temperature gradients will also alter the overall selectivity, as the conversion process occurs across a range of different temperatures. In addition, it is important to recognize the possible formation of solid carbon (and ensuing operational challenges) for gas mixtures with excess CH_4_,^20,38–41^ which can result from C_2_H_2_ and C_2_H_4_ formation, as these are important precursor species.^23,42,43^ This phenomenon has not been accounted for in our study. Nonetheless, the aforementioned results offer qualitative insights into the influence of post-plasma mixing on product selectivities.

#### 3.2.3. Effect on energy cost

In addition to analysing the chemistry, we highlight the impact of this post-plasma mixing approach on energy costs, demonstrating its theoretical viability as a heat recovery system and offering a more comprehensive evaluation of its potential efficiency.

In this case, only 10% of the total gas flow is treated directly with plasma (instead of the complete gas flow), hence the minimum energy input is ten times lower compared to the previous conditions in Section 3.1.4 (Fig. S8†). The minimum energy input ranges between 0.75 and 3.7 kJ L^−1^, increasing with targeted plasma temperature and depending on the CO_2_/CH_4_ ratio.

**Fig. 12:**
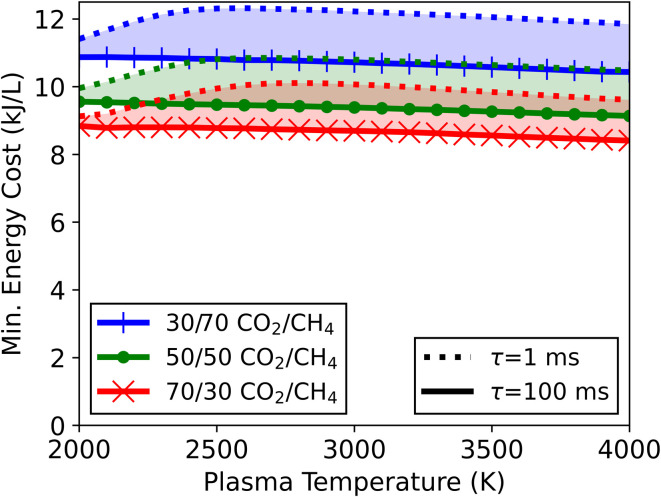
Minimum energy cost of conversion as a function of plasma temperature, for three different CO_2_/CH_4_ ratios (70/30, 50/50, 30/70). The range between the slowest mixing (*τ*_mix_ = 100 ms, solid lines) and the fastest mixing (*τ*_mix_ = 1 ms, dotted lines) is indicated.

The calculated minimum energy cost of conversion for the three different gas mixtures is shown in Fig. 12, with the optimal results achieved for the slowest mixing (*τ*_mix_ = 100 ms). The energy cost slightly decreases with rising plasma temperature for all gas mixtures. The 30/70 CO_2_/CH_4_ ratio has the highest overall minimum energy cost. It decreases from 10.9 to 10.4 kJ L^−1^ when the plasma temperature is raised from 2000 to 4000 K. The stoichiometric (50/50) and 70/30 ratios have slightly lower values which follow the same trend, decreasing from 9.6 to 9.1 kJ L^−1^ and from 8.8 to 8.4 kJ L^−1^, respectively. Increasing the mixing rate tempers the additional conversion, increasing the minimum energy cost (see Fig. 12). At 2000 K, the difference in energy cost between *τ*_mix_ = 100 ms and 1 ms is less than 0.5 kJ L^−1^, for all mixtures, because the impact of mixing is very minor. For higher plasma temperatures the effects of mixing are more significant. Indeed, as the faster mixing limits the additional conversion, the minimum energy cost rises, which increases the energy cost disparity between *τ*_mix_ = 100 ms and 1 ms to 1.4–1.5 kJ L^−1^ at 2900 K, depending on the gas mixture.

These results contrast those discussed in Section 3.1.4 (in the absence of mixing), where the energy cost always increased with plasma temperature. The higher temperatures reached in those results are unfavourable due to overheating of the gas, not having the option to be reused effectively. However, by applying mixing, additional conversion can be achieved, creating a use for this excess heat. For a plasma temperature of 4000 K, the mixing allows a reduction in energy cost of between 19 and 29 kJ L^−1^ (depending on the CO_2_/CH_4_ ratio) compared to the results discussed in Section 3.1.4, which corresponds to a relative drop in energy cost of 68 to 78%. Note that when a high fraction of gas is treated in the reactor, one can argue that it is equally useful to increase the flow rate through the plasma to decrease the specific energy input, thereby operating at a lower temperature, which can still achieve the same conversion, instead of using post-plasma mixing. However, for reactors in which the treated gas fraction is limited, increasing the mixing with the surrounding unconverted gas does have a benefit on the overall performance (for the same energy input).

The nuances previously discussed should also be applied here. As our results are derived from an idealised setup, the actual energy cost in the experiments will be higher because of various thermal losses and non-uniformity of the plasma. Also, our modelling approach considers a discrete temperature difference between the high temperature plasma zone and the cold surrounding gas, which are eventually mixed in the post-plasma region. In plasma reactors, a temperature gradient will exist on the interface between the two zones. On the one hand, this will increase the overall energy input assuming the same plasma fraction and temperature. However, on the other hand, partial conversion can also occur in this gradient zone as temperatures will approach that of the plasma. Although uncaptured by the model, this effect will also influence the overall effect of mixing in the post-plasma region. This aspect of mixing is subject to further research, possibly using higher dimensional modelling that allows for more detailed studies of heat transport phenomena. Moreover, since a certain degree of post-plasma mixing will already be present in experimental setups, this effect is intrinsically always in place. As a result, further enhancing this mixing will be less beneficial than predicted by our model (since the model assumes no prior mixing). This is supported by Sun *et al.*,^37^ who determined in their reactor network model for a DRM microwave setup that the heat loss to the wall is on a longer timescale than the mixing and subsequent reactions. Consequently, mixing plays an integral role in the reforming process within their system. These observations reinforce the importance of accounting for this effect.

Nevertheless, mixing the hot plasma effluent with cold new gas has the potential to greatly improve the system's energy efficiency. This strategy represents an effective implementation of a heat recovery system, reusing the energy applied in the plasma by harnessing the generated heat post-plasma, which would otherwise just be dissipated and lost. Furthermore, this strategy could also be combined with a complete heat recovery system, reusing the energy for preheating the plasma, so that the applied plasma power can effectively (all) be used for the chemical conversion. This can both be thought as an optimisation method, particularly well-suited for setups with localised, high temperature plasmas. Therefore, post-plasma mixing is an important consideration in the design and optimisation of reactors for DRM processes and further development of plasma technology in general.

## 4. Conclusion

In this work, we studied the post-plasma DRM kinetics for warm plasmas, in a wide range of plasma temperatures, and for different CO_2_/CH_4_ ratios and cooling/mixing methods. Firstly, we evaluated enhanced conductive cooling to decrease the afterglow temperature, thereby gaining insights into the effect of heat quenching on the DRM chemistry.

A negligible effect of quenching was found for CO_2_/CH_4_ mixtures with ratios of 50/50 and 30/70, which maintained the near 100% conversion through the afterglow regardless of the quenching rate. However, for mixtures with excess CO_2_ (70/30), 100% conversion could only be achieved in the plasma region at temperatures of 4000 K. Our model indicates that the conversion diminishes throughout the afterglow, due to the occurrence of radical recombination reactions towards CO_2_, H_2_ and H_2_O, and a subsequent water gas shift reaction. For plasma temperatures below 2300 K, only the water gas shift reaction is relevant and its effect is reduced with faster quenching rates, resulting in more CO and H_2_O formation. On the other hand, for higher plasma temperatures (above 2800 K) the effect of water gas shift is minor compared to the radical recombination reactions. Increasing the quenching rate in the afterglow forces these radical recombination pathways to occur at lower temperatures, favouring the formation of CO_2_ (and H_2_) over H_2_O. While this effect is detrimental in terms of CO_2_ conversion, the syngas ratio (H_2_/CO) is enhanced and the concentration of unwanted H_2_O is simultaneously lowered, thus producing a more valuable effluent. This may be beneficial in terms of energy use towards production of these desired species. In general, we can conclude that heat quenching in the afterglow of DRM plasmas only has a significant impact for mixtures with excess CO_2_. Regarding the minimum energy cost of conversion, our calculations suggest that it is best to keep the plasma temperature as low as possible, around 2000 K, considering the assumption of a homogeneous plasma at a constant temperature. This is explained by the minor difference in total overall conversion, which shows only a slight variation with plasma temperature, whereas the minimum energy required to achieve these higher temperatures increases significantly.

Furthermore, we showed that implementing post-plasma mixing of cold fresh CO_2_ and CH_4_ can significantly boost the overall output of syngas (and other side products). This method essentially allows to recover energy from the plasma and use it to convert more reactant gas, improving the overall performance. The additional conversion rises with plasma temperature and slower mixing, reaching a maximum additional conversion, relative to the conversion in the plasma, of 258 and 301% for CO_2_ and CH_4_, respectively, within the tested parameter ranges in this study. The model also shows that stronger mixing limits the additional conversion, which is logical since the faster the mixing, the shorter the residence times at sufficiently elevated temperature (which is the main conversion driver). The post-plasma mixing also leads to minor changes in product selectivity. Upon the temperature drop, brought forth by the mixing, the conversion pathways are interrupted. Consequently, this leads to low selectivity towards intermediate species (H_2_O and C_2_H_4_ for the 30/70 ratio, H_2_O and C_2_H_2_ for the 50/50 ratio and C_2_H_2_ for the 70/30 ratio).

Our model reveals that significant reductions in energy cost are theoretically possible (up to 78%) by implementing this ‘heat recovery system’ through post-plasma mixing. However, under experimental conditions, heat transfer from the gas to the reactor wall can reduce the overall benefit by limiting the additional conversion compared to our idealised conditions. Nonetheless, our study demonstrates that post-plasma mixing can create opportunities to optimise DRM in warm plasmas.

## Data availability

The data supporting this article have been included as part of the ESI.†

## Conflicts of interest

There are no conflicts to declare.

## Supplementary Material

SU-003-D4SU00676C-s001
