# Supplementary material for: Afterglow quenching in plasma-based dry reforming of methane: a detailed analysis of the post-plasma chemistry via kinetic modelling
Source: RSC Sustain. 2025 Jan 28;3(3):1477–93. doi: 10.1039/d4su00676c (PMC11783141; doi:10.1039/d4su00676c)
Supplement: SU-003-D4SU00676C-s001 [file SU-003-D4SU00676C-s001.pdf]

Supplementary Information (SI) for RSC Sustainability.
This journal is © The Royal Society of Chemistry 2025

Supporting Information: Afterglow quenching in plasma-based dry reforming of methane: a detailed analysis of the post-plasma chemistry via kinetic modelling.

Joachim Slaets, Eduardo Morais and Annemie Bogaerts

Research group PLASMANT, Department of Chemistry, University of Antwerp, Universiteitsplein 1, BE-2610 Wilrijk-Antwerp, Belgium

S1 Model equations

The mass balance equation (Eq. 1 in the main paper) requires the rate (R_i) for each reaction i (as given by Eq. S1), which is the product of the rate coefficient k_i and number densities of the reactants n_s. These rate coefficients are given by analytical equations, e.g., modified Arrhenius equations or fall-off functions. A complete list of all reactions in the kinetics scheme, with the corresponding rate coefficients or cross sections and references, is given in Table S3 at the end of the document.

$$R_i = k_i \prod_s n_s^{a_s^L} \qquad \text{(Eq. S1)}$$

The correction for the gas expansion is added to the mass balance equation of each species (see last term in Eq. 1 in the main paper) and consists of two terms, i.e., reactive expansion and thermal expansion. The former term accounts for the total number density increase or decrease caused by chemical reactions and the latter for changes due to temperature changes. This correction parameter is given in Eq. S2, in which n_s is the number density of the species s for which the mass balance is solved, n_j the number density of all species in the model j, $a^R_{j,i}$ and $a^L_{j,i}$ the coefficients of j in reaction i, R_i the rate of reaction i, R_{mix} the rate of gas mixing (when applicable), $\partial T/\partial t$ the temperature change with respect to time, k_B the Boltzmann constant, P_0 the pressure (1 atm) and T the gas temperature.

$$R_{expansion} = -\frac{n_s}{\sum_j n_j}\left(\sum_j \sum_{i=1}^{j} \left[\left(a^R_{j,i} - a^L_{j,i}\right) \cdot R_i\right] + R_{mix}\right) - \frac{n_s}{\sum_j n_j}\frac{\partial T}{\partial t}\frac{P_0}{k_B T^2} \qquad \text{(Eq. S2)}$$

Furthermore, as explained in the main paper, the heat balance equation is solved in the post-plasma region (see Eq. 4 in the main paper). The isobaric heat capacity of the gas mixture $C_{p,mix}$, used in the self-consistent temperature calculation, is given in Eq. S3. This is calculated as the sum of the heat capacity $C_{p,i}$ of the individual species i (obtained from McBride et al.[1] and Burcat et al.[2]) weighted to the number density n_i of the species i over the total number density n_{tot}.

$$C_{p,mix}(T) = \sum_i C_{p,i}(T)\frac{n_i}{n_{tot}} \qquad \text{(Eq. S3)}$$

The temperature-dependent reaction enthalpy ε is calculated for reaction i using Eq. S4, in which $a^R_{s,i}$ and $a^L_{s,i}$ are the coefficients of species s in reaction i at the right and left side of the reaction, respectively, and ΔH^f_s the temperature-dependent enthalpy of formation of species s (obtained from McBride et al.[1] and Burcat et al.[2]).

$$\epsilon_i(T) = \sum_s [a^R_s - a^L_s]\ H^f_s(T) \qquad \text{(Eq. S4)}$$

To calculate the thermal loss, a thermal conductivity is required and this is calculated as the mixture-averaged conductivity using the Mason Saxena equation (Eq. S5),[3] in which i and k are the species in the model, λ_i is their temperature-dependent thermal conductivity (obtained from the polynomials provided by McBride et al.[4]), x_i and x_k are their molar fractions and G_{ik} is a factor calculated using Eq. S6.

$$\lambda_{mix}(T) = \sum_i \lambda_i(T) \left[1 + \sum_k G_{ik} \frac{x_k}{x_i}\right]^{-1} \qquad \text{(Eq. S5)}$$

The factor G_{ik} for species i respective to species k is given in Eq. S6, in which M is the molar mass and μ the viscosity (obtained from the polynomials provided by McBride et al.[4]).

$$G_{ik} = \frac{1.065}{2\sqrt{2}} \left(1 + \frac{M_i}{M_k}\right)^{-\frac{1}{2}} \left[1 + \left(\frac{\mu_i M_k}{\mu_{kMi}}\right)^{\frac{1}{2}} \left(\frac{M_i}{M_k}\right)^{\frac{1}{4}}\right] \qquad \text{(Eq. S6)}$$

S2 Chemistry

In this work we use a subset of the reaction scheme used in our previous work, [5] more specifically we only use the thermal reactions (i.e., electrons and ions and their respective reactions are not included), as we demonstrated before that the thermal chemistry is dominant at the conditions under study here. Most rate coefficients are obtained directly from literature sources, with some exceptions. Indeed, for reverse reactions between neutral species for which no reliable source could be found in literature, detailed balancing is used to obtain the rate coefficients. The equilibrium constant K_{eq} is calculated using Eq. S7, with p the reference pressure (1 bar), Δv the change in number of species in the reaction and ΔG_r the Gibbs free energy of the reaction, calculated using thermodynamic data from McBride et al. [1] and Burcat et al.[2].

$$K_{eq} = \left(\frac{p}{RT}\right)^{\Delta v} e^{\left(\frac{-\Delta G_r}{RT}\right)} \qquad \text{(Eq. S7)}$$

For the full list of the included reactions with the corresponding rate coefficients and respective references, see Table S3, at the end of this document.

S3 Additional results

S3.1 Post-plasma conductive cooling

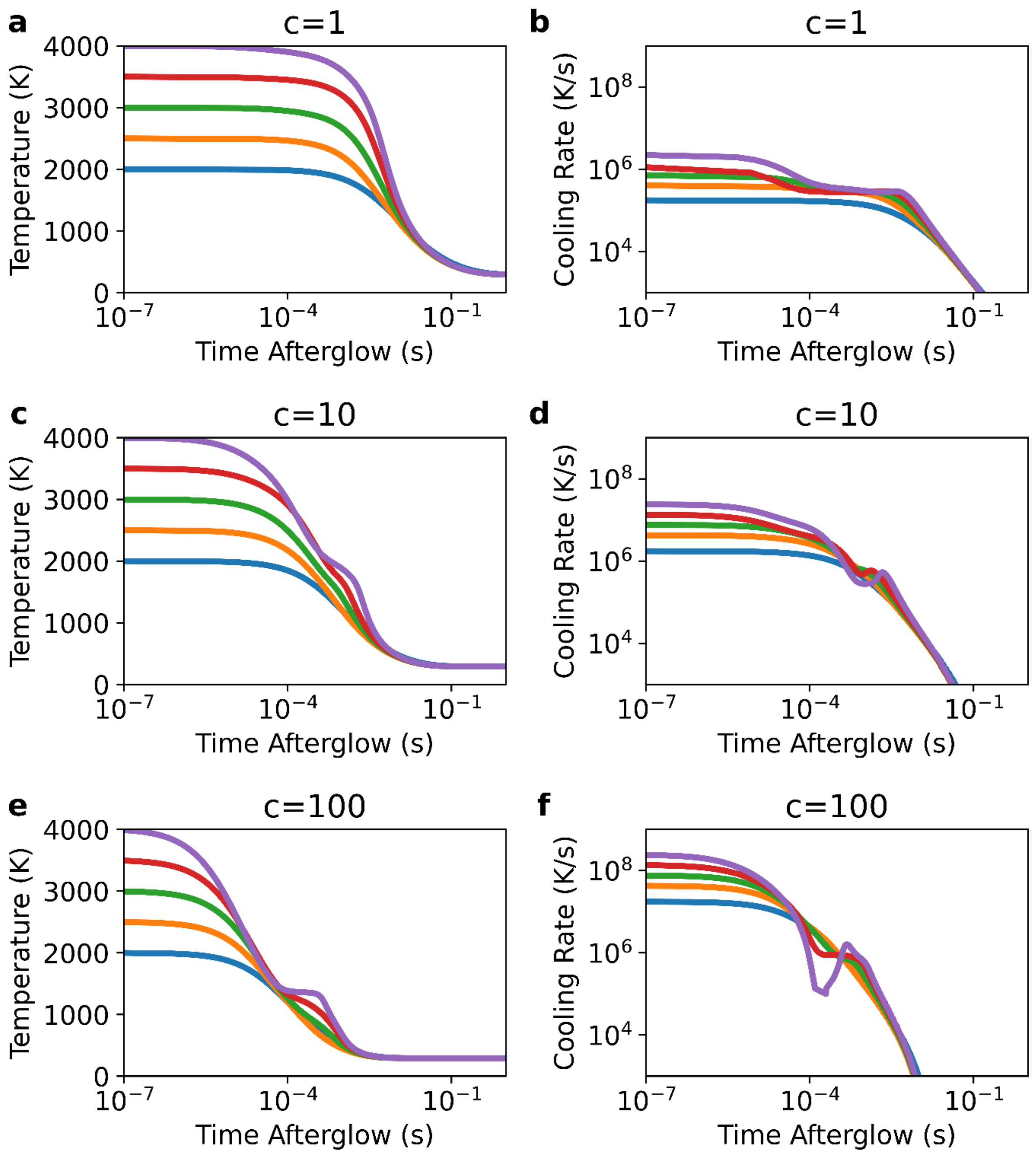

Figure S1. Gas temperature profiles (left panels a, c, e) and cooling rates (right panels b, d, f) as a function of time in the afterglow for the 50/50 CO_2/CH_4 gas mixture, starting from plasma temperatures of 2000, 2500, 3000, 3500 and 4000 K, for quenching with c-factors of 1 (a, b), 10 (c, d) and 100 (e, f).

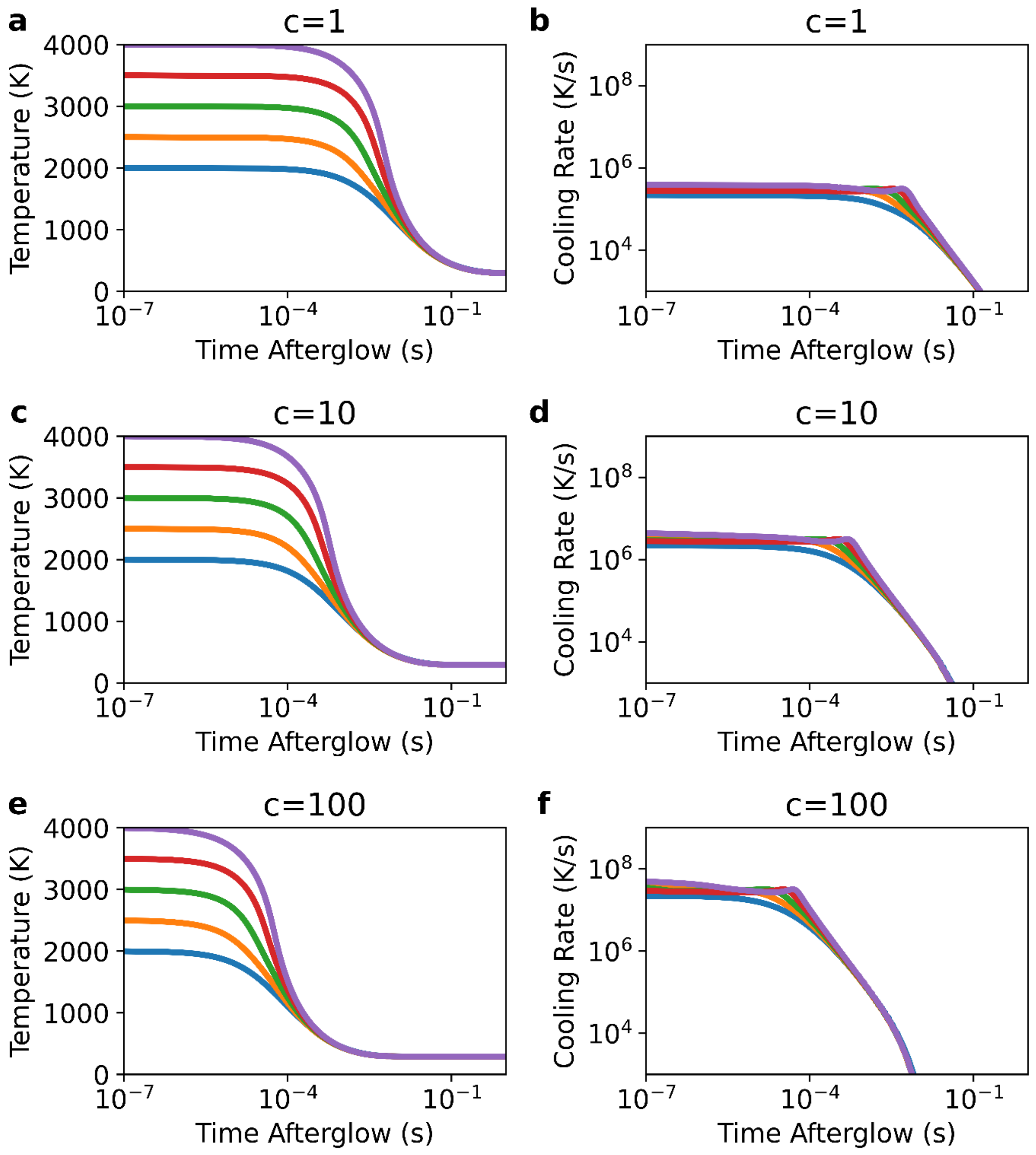

Figure S2. Gas temperature profiles (left panels a, c, e) and cooling rates (right panels b, d, f) as a function of time in the afterglow for the 30/70 CO_2/CH_4 gas mixture, starting from plasma temperatures of 2000, 2500, 3000, 3500 and 4000 K, for quenching with c-factors of 1 (a, b), 10 (c, d) and 100 (e, f).

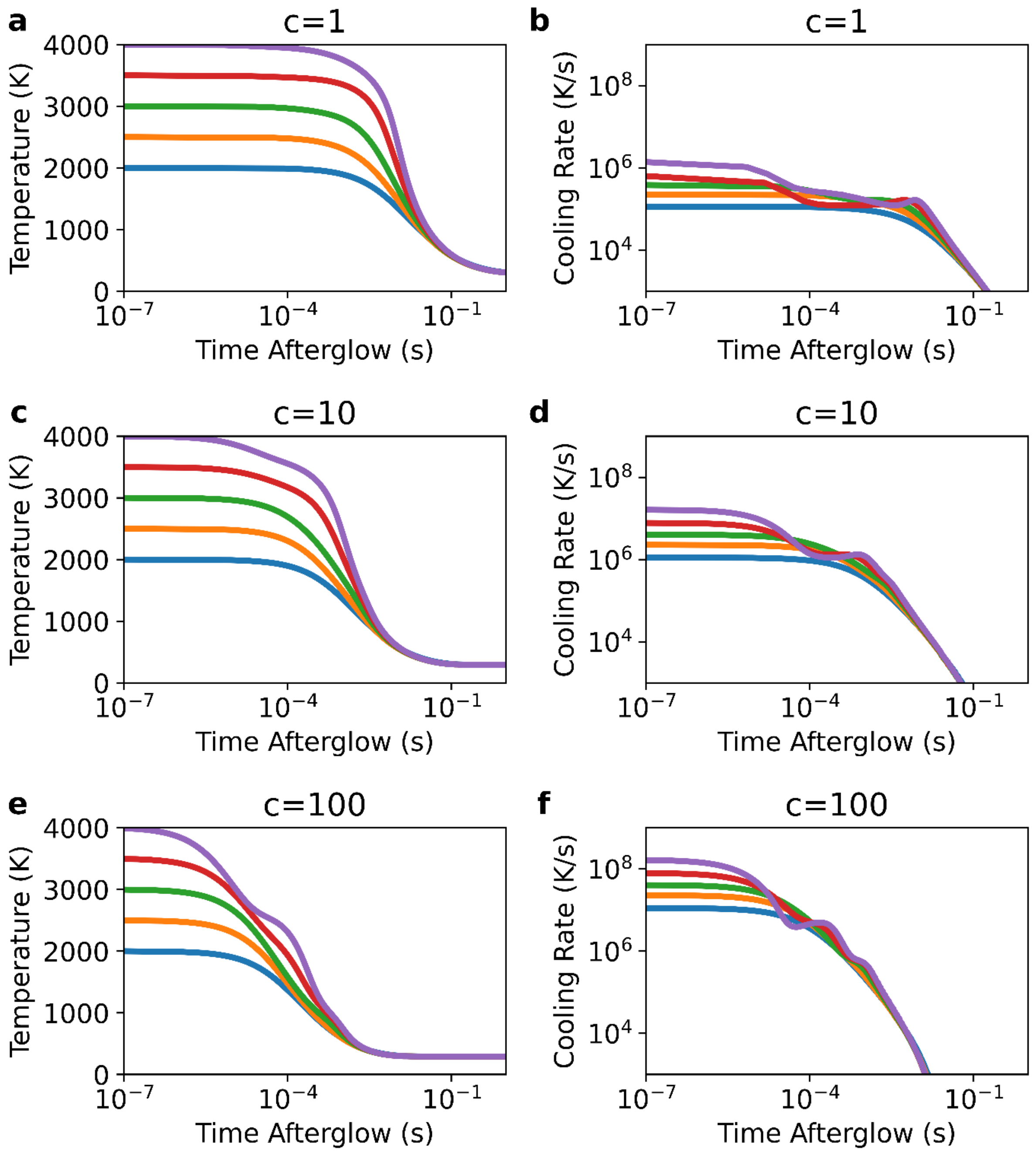

Figure S3. Gas temperature profiles (left panels a, c, e) and cooling rates (right panels b, d, f) as a function of time in the afterglow for the 70/30 CO_2/CH_4 gas mixture, starting from plasma temperatures of 2000, 2500, 3000, 3500 and 4000 K, for quenching with c-factors of 1 (a, b), 10 (c, d) and 100 (e, f).

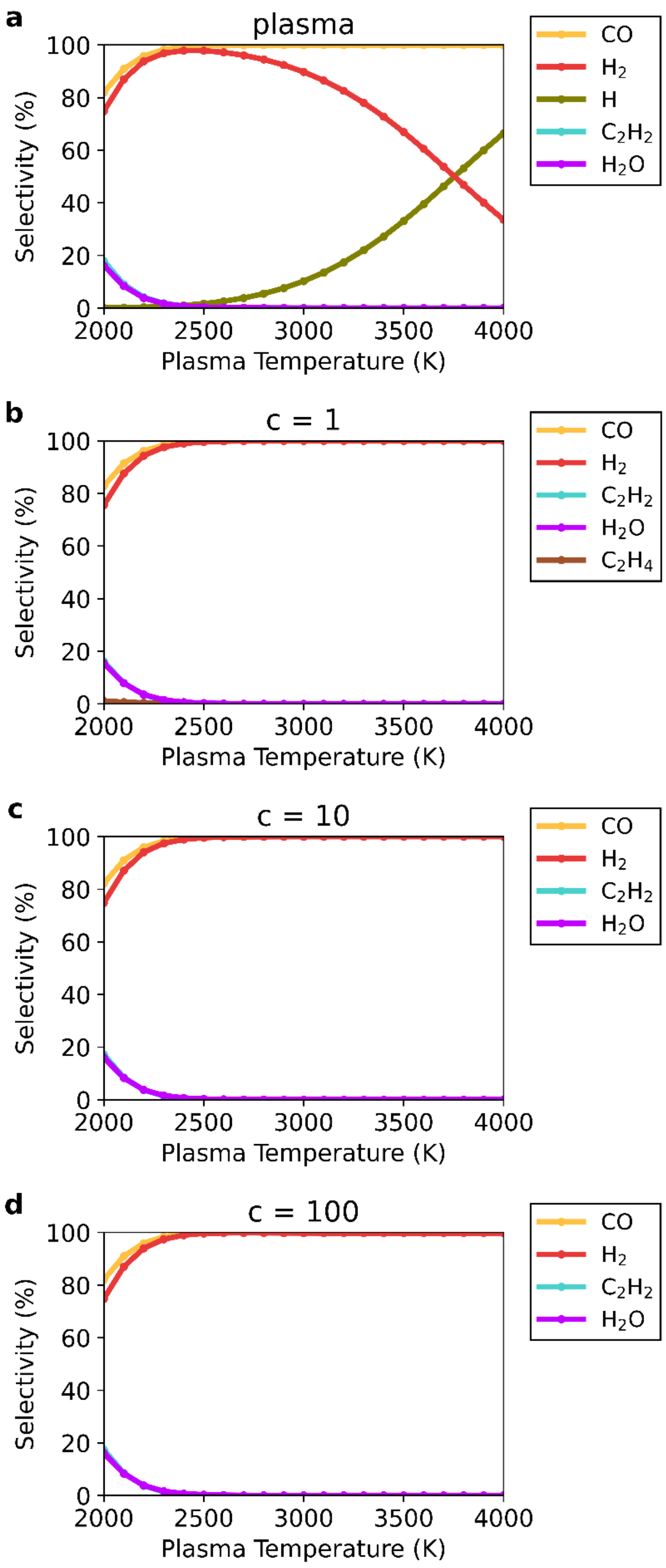

Figure S4. Selectivity of the main species (above 1%), as a function of the plasma temperature for the 50/50 CO_2/CH_4 ratio, at the end of the plasma (a), and at the end of the afterglow (b, c, d), for c-factors of 1 (b), 10 (c) and 100 (d). The H_2 and CO selectivity curves and the H_2O and C_2H_2 selectivity curves overlap.

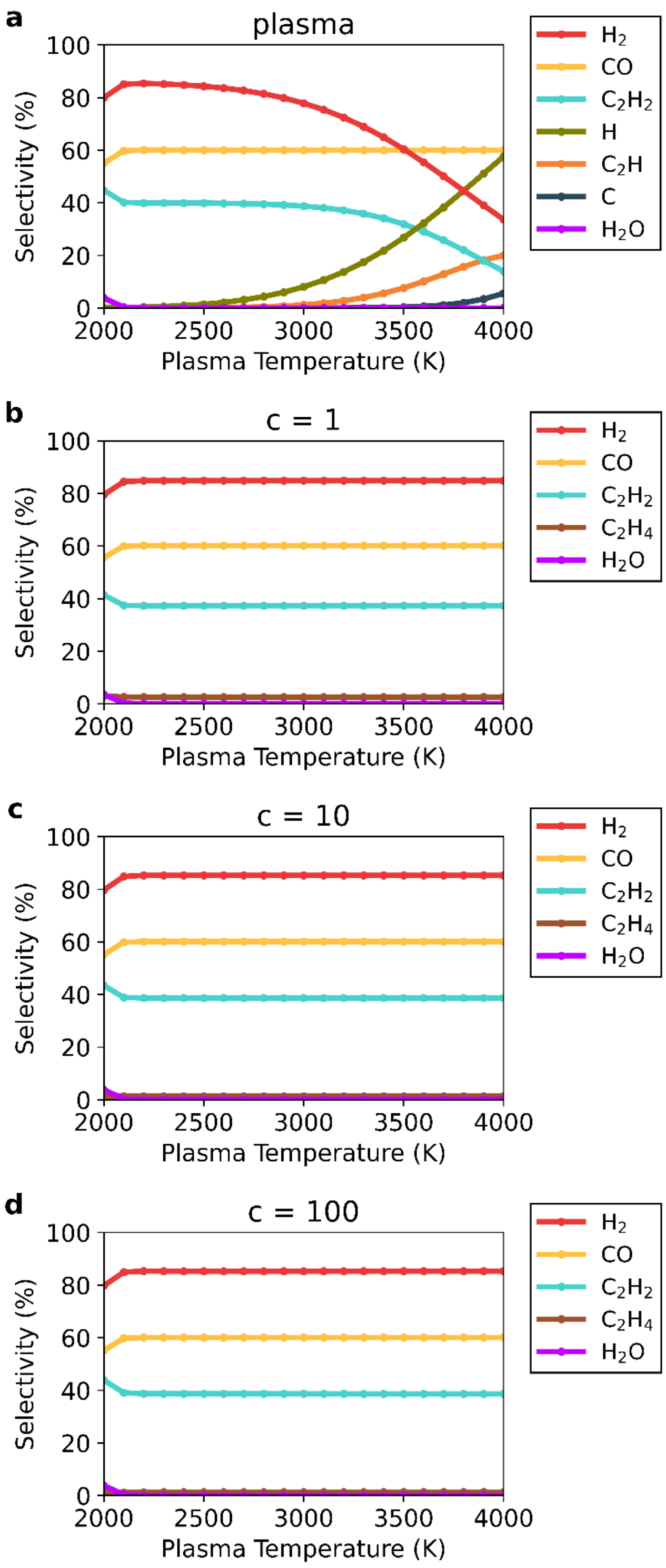

Figure S5. Selectivity of the main species (above 1%), as a function of the plasma temperature for the 30/70 CO_2/CH_4 ratio, at the end of the plasma (a), and at the end of the afterglow (b, c, d), for c-factors of 1 (b), 10 (c) and 100 (d). The H_2O and C_2H_4 selectivity curves overlap.

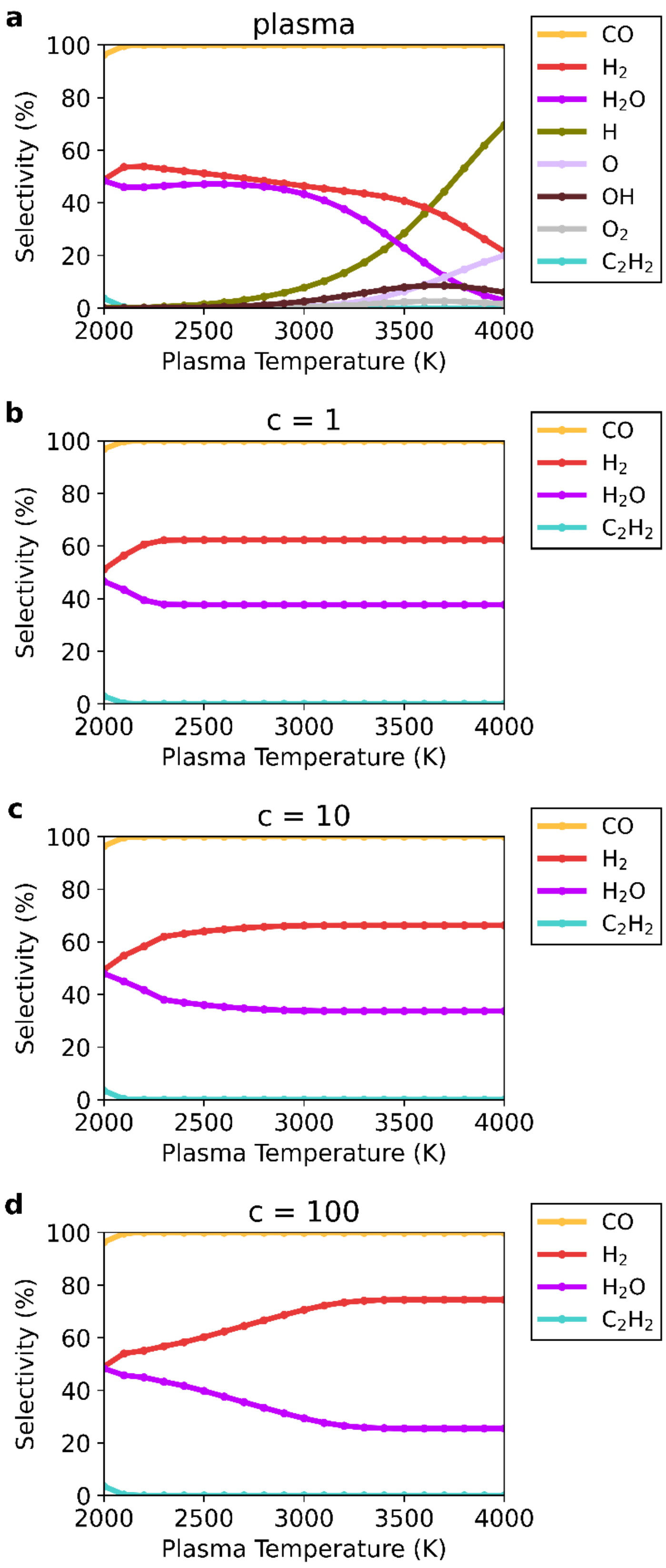

Figure S6. Selectivity of the main species (above 1%), as a function of the plasma temperature for the 70/30 CO_2/CH_4 ratio, at the end of the plasma (a), and at the end of the afterglow (b, c, d), for c-factors of 1 (b), 10 (c) and 100 (d).

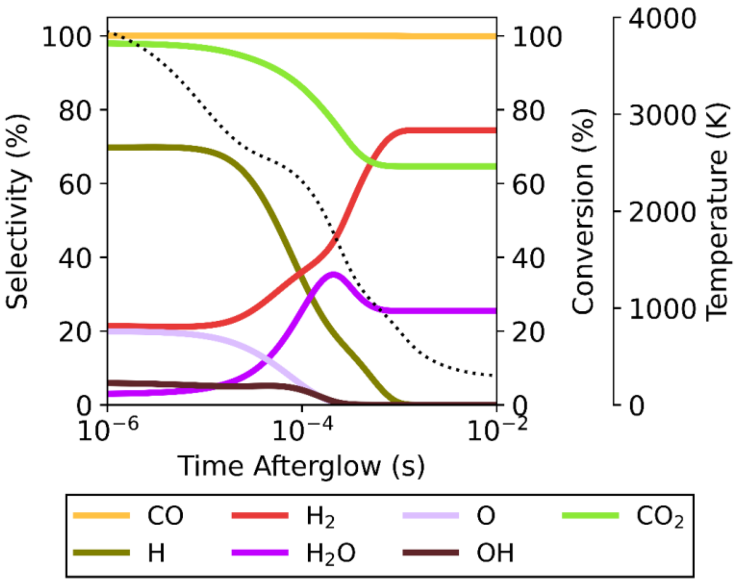

Figure S7. Time-evolution of the selectivity of the main species in the afterglow, starting from a plasma temperature of 4000 K for the 70/30 CO_2/CH_4 ratio and c-factor = 100. The evolution of the CO_2 conversion (lime green curve) and the gas temperature (dotted line) are also plotted, and shown on the right axis.

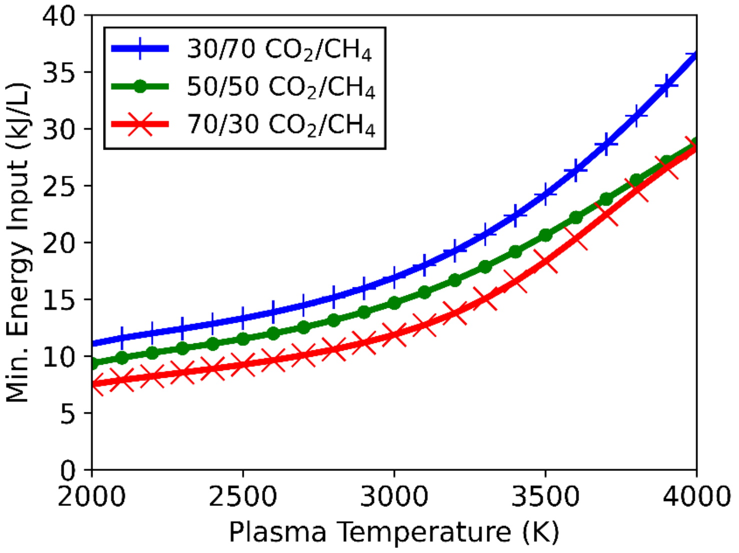

Figure S8. Minimum energy input required to achieve the final species distribution at the end of the plasma as a function of plasma temperature for the three different CO_2/CH_4 ratios (70/30, 50/50, 30/70).

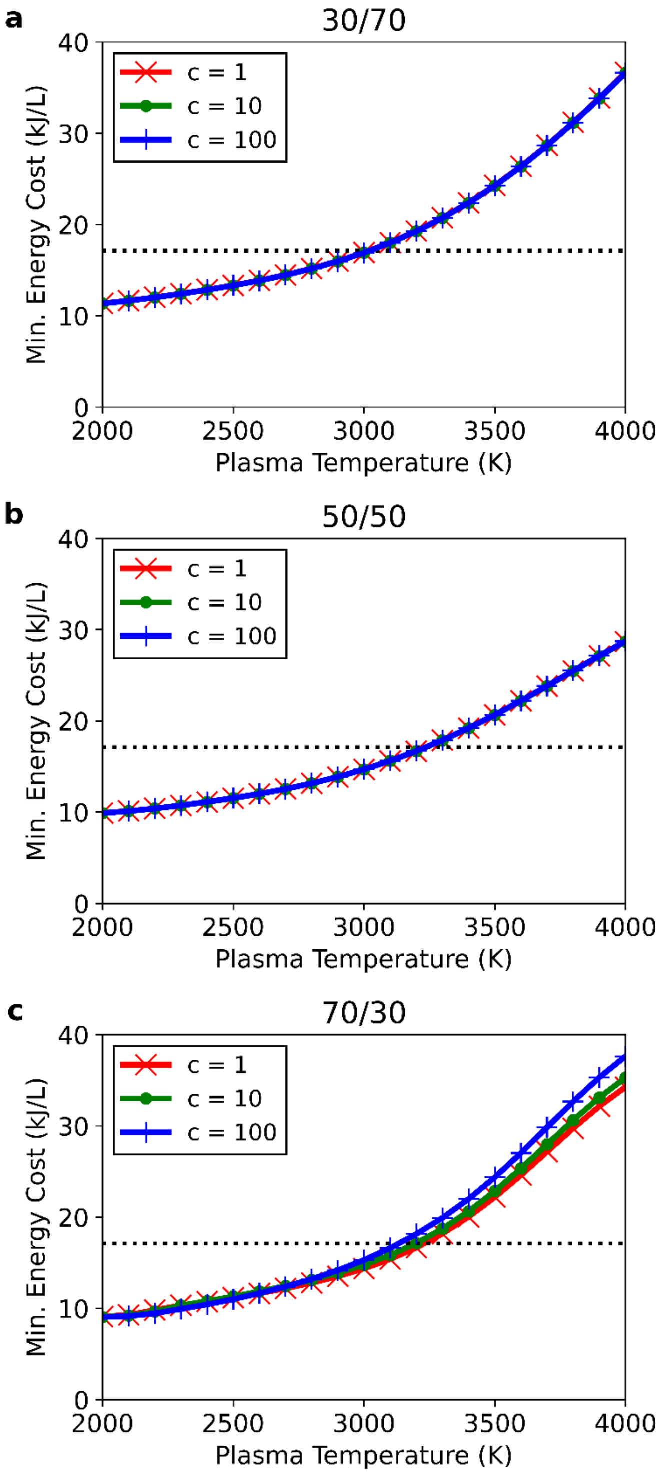

Figure S9. Minimum energy cost of conversion for c-factors of 1, 10 and 100 as a function of plasma temperature for CO_2/CH_4 ratios of 30/70 (a), 50/50 (b) and 70/30 (c). The horizontal dotted black line indicates the target energy cost value of 17.1 kJ/L (4.27 eV/molecule) proposed by Snoeckx and Bogaerts for plasma-based DRM to be competitive with existing technologies.[6]

S3.2 Post-plasma mixing

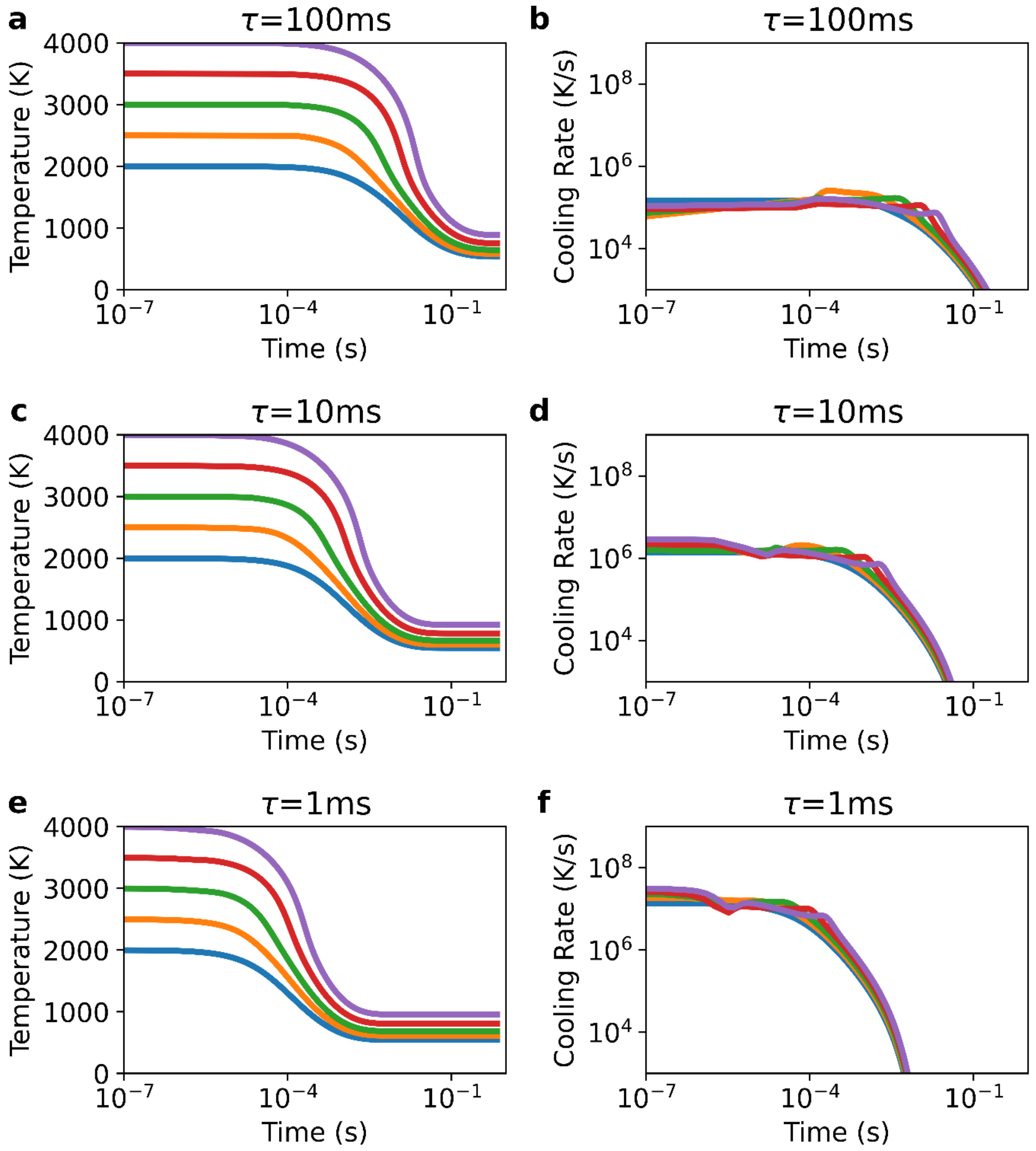

Figure S10. Gas temperature profiles (left panels a, c, e) and cooling rates (right panels b, d, f) as a function of time in the afterglow for the 50/50 CO_2/CH_4 gas mixture, starting from plasma temperatures of 2000, 2500, 3000, 3500 and 4000 K, for characteristic mixing times of τ_{mix} = 100 ms (a, b), 10 ms (c, d) and 1 ms (e, f).

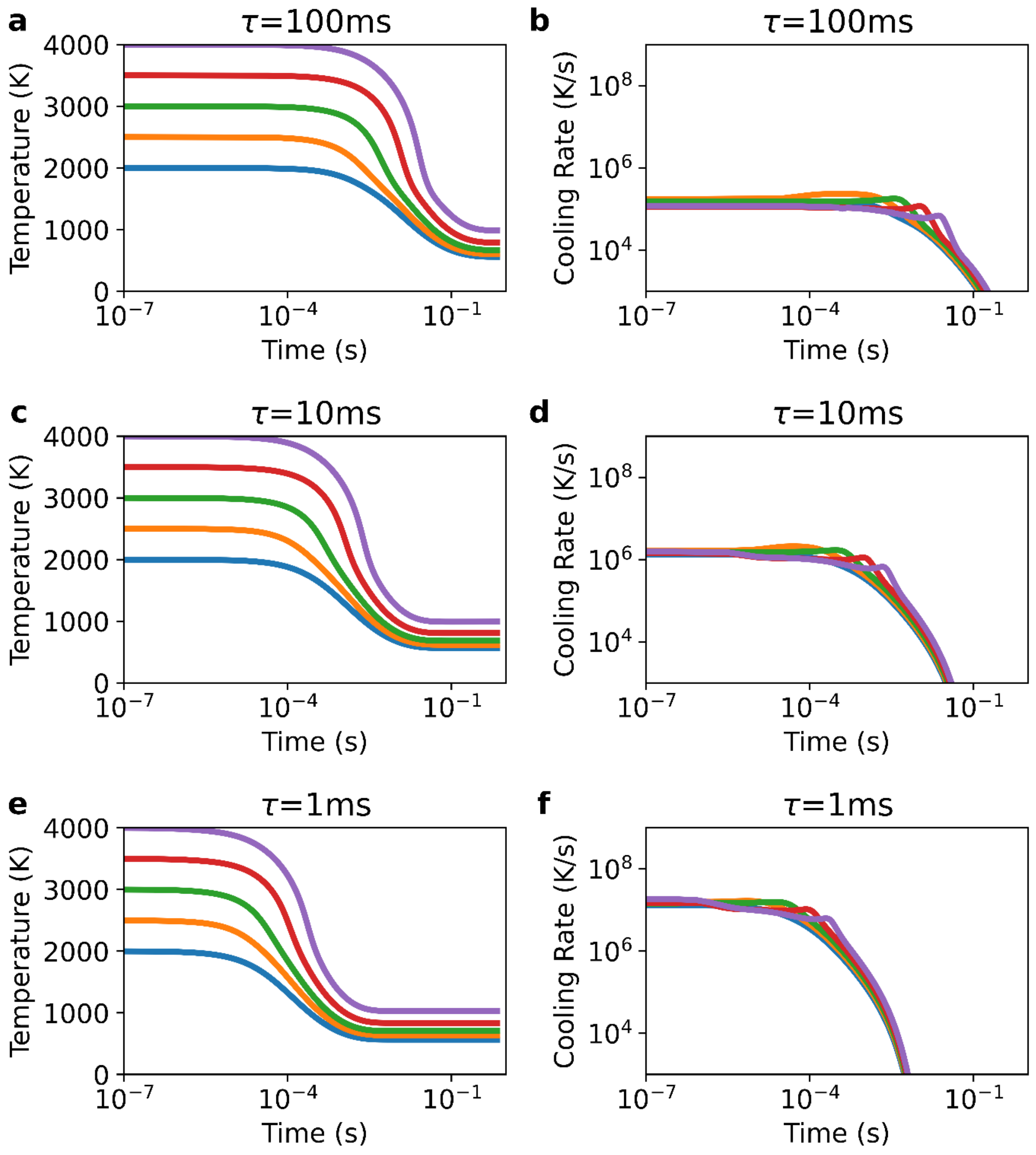

Figure S11. Gas temperature profiles (left panels a, c, e) and cooling rates (right panels b, d, f) as a function of time in the afterglow for the 30/70 CO_2/CH_4 gas mixture, starting from plasma temperatures of 2000, 2500, 3000, 3500 and 4000 K, for characteristic mixing times of τ_{mix} = 100 ms (a, b), 10 ms (c, d) and 1 ms (e, f).

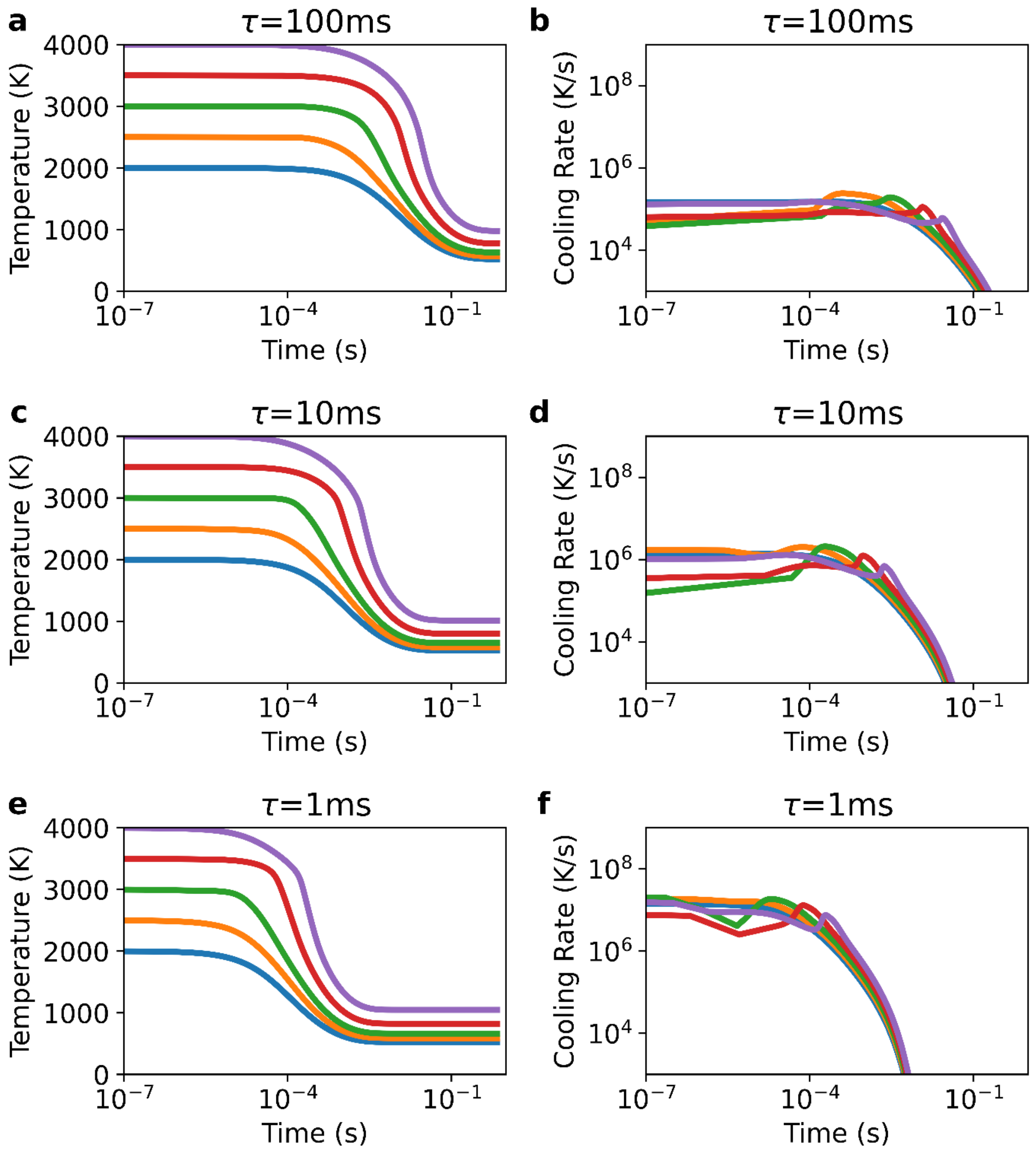

Figure S12. Gas temperature profiles (left panels a, c, e) and cooling rates (right panels b, d, f) as a function of time in the afterglow for the 70/30 CO_2/CH_4 gas mixture, starting from plasma temperatures of 2000, 2500, 3000, 3500 and 4000 K, for characteristic mixing times of τ_{mix} = 100 ms (a, b), 10 ms (c, d) and 1 ms (e, f).

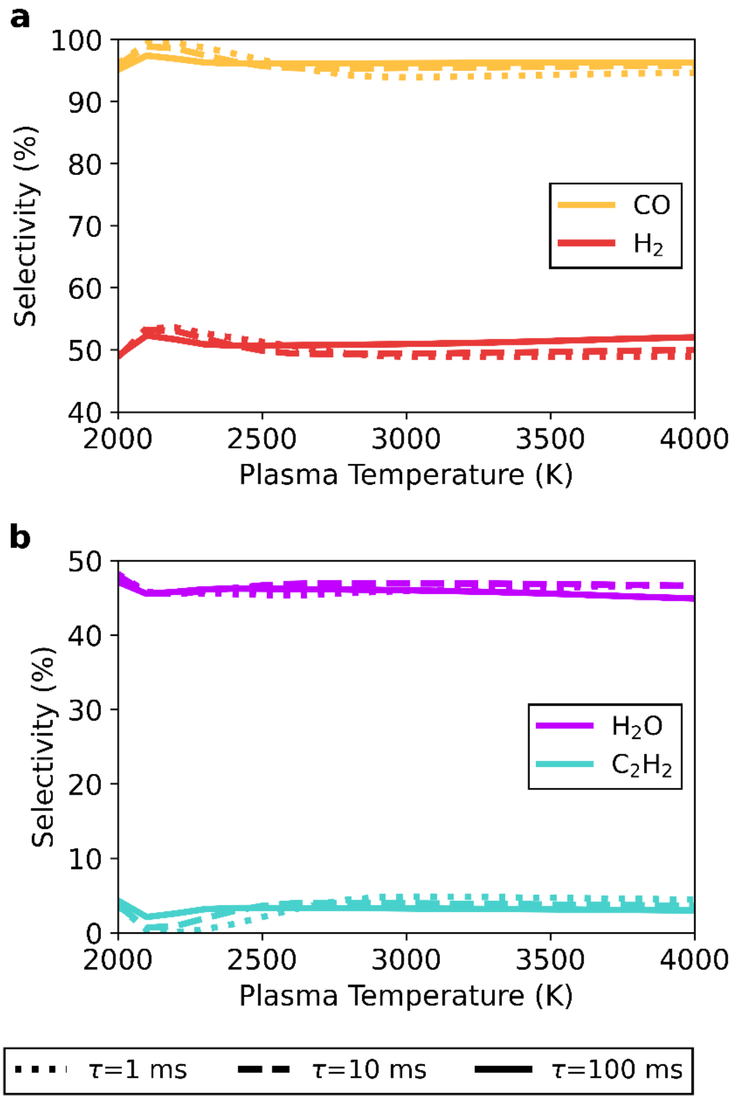

Figure S13. Selectivity at the end of the afterglow towards the main product species (H_2 and CO in panel a, and C_2H_2 and H_2O in panel b) as a function of the plasma temperature, at the 70/30 CO_2/CH_4 ratio and $\tau_{mix} = 1$ (dotted line), 10 (dashed line) and 100 (solid line) ms.

Table S1. CO_2 conversion obtained at the end of the plasma (Plasma), end of the afterglow (Afterglow) and relative additional conversion, calculated using equation 12 (Additional (Relative)) for plasma temperatures between 2000 and 4000 K and three different CO_2/CH_4 ratios (70/30, 50/50, 30/70) at τ_{mix} = 10 ms.

T_{plasma}	CO_2 conversion (%)								
	70/30			50/50			30/70		
	Plasma	Afterglow	Additional (Relative, %)	Plasma	Afterglow	Additional (Relative, %)	Plasma	Afterglow	Additional (Relative, %)
2000	77	7.7	-0.65	93	9.3	-0.32	98	9.8	-0.070
2100	81	8.1	-0.30	97	9.7	0.59	100	10	1.2
2200	82	8.3	0.70	99	10	2.4	100	10	3.4
2300	83	8.5	2.6	99	10	5.0	100	11	6.5
2400	83	8.8	5.0	100	11	8.5	100	11	10
2500	84	9.1	8.3	100	11	12	100	11	14
2600	84	9.5	12	100	12	17	100	12	19
2700	84	9.9	17	100	12	22	100	12	24
2800	85	10	23	100	13	28	100	13	31
2900	85	11	29	100	14	35	100	14	38
3000	86	12	37	100	14	44	100	15	47
3100	87	13	46	100	15	53	100	16	56
3200	88	14	57	100	16	64	100	17	68
3300	89	15	69	100	18	77	100	18	81
3400	90	17	84	100	19	90	100	20	96
3500	92	19	101	100	21	105	100	21	114
3600	93	21	121	100	22	122	100	23	133
3700	95	23	141	100	24	139	100	25	155
3800	96	25	161	100	26	156	100	28	178
3900	97	27	180	100	27	174	100	30	204
4000	98	29	197	100	29	190	100	33	230

Table S2. CH_4 conversion obtained at the end of the plasma (Plasma), end of the afterglow (Afterglow) and relative additional conversion, calculated using equation 12 (Additional (Relative)) for plasma temperatures between 2000 and 4000 K and three different CO_2/CH_4 ratios (70/30, 50/50, 30/70) at τ_{mix} = 10 ms.

T_{plasma}	CH_4 conversion (%)								
	70/30			50/50			30/70		
	Plasma	Afterglow	Additional (Relative, %)	Plasma	Afterglow	Additional (Relative, %)	Plasma	Afterglow	Additional (Relative, %)
2000	97	9.7	0.18	96	9.6	0.21	97	9.8	0.33
2100	99	10	1.1	98	9.9	1.2	99	10	1.6
2200	100	10	3.4	99	10	3.1	100	10	4.0
2300	100	11	7.3	100	11	5.9	100	11	7.3
2400	100	11	12	100	11	9.9	100	11	11
2500	100	12	17	100	11	14	100	12	16
2600	100	12	22	100	12	20	100	12	20
2700	100	13	28	100	13	26	100	13	26
2800	100	13	34	100	13	32	100	13	32
2900	100	14	42	100	14	40	100	14	39
3000	100	15	51	100	15	48	100	15	48
3100	100	16	63	100	16	58	100	16	58
3200	100	18	77	100	17	69	100	17	69
3300	100	19	94	100	18	82	100	18	82
3400	100	21	114	100	20	96	100	20	98
3500	100	24	138	100	21	111	100	21	115
3600	100	27	165	100	23	127	100	23	134
3700	100	29	194	100	24	144	100	26	155
3800	100	32	223	100	26	162	100	28	179
3900	100	35	251	100	28	180	100	30	203
4000	100	37	275	100	30	197	100	33	230

Table S3: List of reactions with the rate coefficients (third column) expressed in $cm^3\ s^{-1}$ for two-body reactions, and in $cm^6\ s^{-1}$ for three-body reactions and references (fourth column). In the rate equations, N_A is Avogadro's constant, k_B is the Boltzmann constant, R is the ideal gas constant, T_g is the gas temperature in K and n_M is the total number density of neutral species in cm^{-3}.

#	Reaction	Rate equation	Ref.
1	$CH_4 + H \rightarrow CH_3 + H_2$	$6.4 \times 10^{-18} \cdot T_g^{2.11} \cdot \exp\left(\frac{-3.9\times10^3}{T_g}\right)$	[7]
2	$CH_3 + H_2 \rightarrow CH_4 + H$	$6.62 \times 10^{-20} \cdot T_g^{2.24} \cdot \exp\left(\frac{-3.22\times10^3}{T_g}\right)$	[7]
3	$CH_3 + H \rightarrow CH_2 + H_2$	$2.1 \times 10^{-8} \cdot T_g^{-0.56} \cdot \exp\left(\frac{-8.0\times10^3}{T_g}\right)$	[8]
4	$CH_3 + H \rightarrow CH_4$	$k_0 = 1.7 \times 10^{-24} \cdot T_g^{-1.8}$<br>$k_\infty = 3.5 \times 10^{-10}$<br>$F_c = 0.63 \cdot \exp\left(\frac{-T_g}{3.3150\times10^3}\right)$<br>$+0.37 \cdot \exp\left(\frac{-T_g}{6.10\times10^1}\right)$	[8][a]
5	$CH_2 + H_2 \rightarrow CH_3 + H$	$7.32 \times 10^{-19} \cdot T_g^{2.3} \cdot \exp\left(\frac{-3.6990\times10^3}{T_g}\right)$	[9]
6	$CH_2 + H \rightarrow CH + H_2$	2×10^{-10}	[8]
7	$CH + H_2 \rightarrow CH_2 + H$	$2.9 \times 10^{-10} \cdot \exp\left(\frac{-1.670\times10^3}{T_g}\right)$	[8]
8	$CH + H_2 \rightarrow CH_3$	$k_0 = 4.7 \times 10^{-26} \cdot T_g^{-1.6}$<br>$k_\infty = 8.5 \times 10^{-11} \cdot T_g^{0.15}$<br>$F_c = 0.48$<br>$+0.25 \cdot \exp\left(\frac{-T_g}{3.0\times10^2}\right)$	[8][a]
9	$CH + H \rightarrow C + H_2$	2×10^{-10}	[8]
10	$C + CH_4 \rightarrow C_2H_4$	5×10^{-15}	[10]
11	$C + CH_3 \rightarrow C_2H_2 + H$	8.3×10^{-11}	[11]
12	$C + CH_2 \rightarrow C_2H + H$	8.3×10^{-11}	[11]
13	$CH_3 + CH_4 \rightarrow C_2H_6 + H$	$\frac{8\times10^{13}}{N_A} \cdot \exp\left(\frac{-1.6736\times10^5}{R\cdot T_g}\right)$	[12]
14	$CH_3 + CH_4 \rightarrow C_2H_5 + H_2$	$\frac{1\times10^{13}}{N_A} \cdot \exp\left(\frac{-9.6232\times10^4}{R\cdot T_g}\right)$	[12]
15	$CH_2 + CH_4 \rightarrow CH_3 + CH_3$	$7.14 \times 10^{-12} \cdot \exp\left(\frac{-4.199\times10^4}{R\cdot T_g}\right)$	[13]
16	$CH + CH_4 \rightarrow C_2H_4 + H$	$2.2 \times 10^{-8} \cdot T_g^{-0.94} \cdot \exp\left(\frac{-2.9\times10^1}{T_g}\right)$	[8]
17	$CH_3 + CH_3 \rightarrow C_2H_6$	$k_0 = 3.5 \times 10^{-7} \cdot T_g^{-7} \cdot \exp\left(\frac{-1.39\times10^3}{T_g}\right)$<br>$k_\infty = 6 \times 10^{-11}$<br>$F_c = 0.38 \cdot \exp\left(\frac{-T_g}{7.3\times10^1}\right)$<br>$+0.62 \cdot \exp\left(\frac{-T_g}{1.18\times10^3}\right)$	[8][a]
18	$CH_3 + CH_3 \rightarrow C_2H_5 + H$	$9 \times 10^{-11} \cdot \exp\left(\frac{-8.08\times10^3}{T_g}\right)$	[8]
19	$CH_3 + CH_3 \rightarrow CH_2 + CH_4$	$5.6 \times 10^{-17} \cdot T_g^{1.34} \cdot \exp\left(\frac{-6.791\times10^4}{R\cdot T_g}\right)$	[14]
20	$CH_2 + CH_3 \rightarrow C_2H_4 + H$	1.2×10^{-10}	[8]
21	$CH_2 + CH_2 \rightarrow C_2H_2 + H_2$	$\frac{10^{1.52\times10^1}}{N_A} \cdot \exp\left(\frac{-5\times10^4}{R\cdot T_g}\right)$	[15]
22	$CH + CH \rightarrow C_2H_2$	$\frac{1.2\times10^{14}}{N_A}$	[16]
23	$CH_4 \rightarrow CH_3 + H$	$k_0 = 7.5 \times 10^{-7} \cdot \exp\left(\frac{-4.570\times10^4}{T_g}\right)$<br>$k_\infty = 2.4 \times 10^{16} \cdot \exp\left(\frac{-5.280\times10^4}{T_g}\right)$<br>$F_c = \exp\left(\frac{-T_g}{1.350\times10^3}\right)$<br>$+\exp\left(\frac{-7.8340\times10^3}{T_g}\right)$	[8][a]
24	$CH_3 \rightarrow CH + H_2$	$1.1 \times 10^{-8} \cdot \exp\left(\frac{-4.280\times10^4}{T_g}\right) \cdot n_M$	[8]
25	$CH_3 \rightarrow CH_2 + H$	$1.7 \times 10^{-8} \cdot \exp\left(\frac{-4.560\times10^4}{T_g}\right) \cdot n_M$	[8]
26	$CH_2 \rightarrow CH + H$	$1.56 \times 10^{-8} \cdot \exp\left(\frac{-4.488\times10^4}{T_g}\right) \cdot n_M$	[8]

#	Reaction	Rate equation	Ref.
27	$CH_2 \rightarrow C + H_2$	$5 \times 10^{-10} \cdot \exp\left(\frac{-3.26\times10^4}{T_g}\right) \cdot n_M$	[8]
28	$CH \rightarrow C + H$	$\frac{1.9\times10^{14}}{N_A} \cdot \exp\left(\frac{-3.37\times10^4}{T_g}\right) \cdot n_M$	[11]
29	$C_2H_6 + H \rightarrow C_2H_5 + H_2$	$1.63 \times 10^{-10} \cdot \exp\left(\frac{-4.640\times10^3}{T_g}\right)$	[8]
30	$C_2H_5 + H_2 \rightarrow C_2H_6 + H$	$5.1 \times 10^{-24} \cdot T_g^{3.6} \cdot \exp\left(\frac{-4.253\times10^3}{T_g}\right)$	[8]
31	$C_2H_5 + H \rightarrow CH_3 + CH_3$	7×10^{-11}	[8]
32	$C_2H_5 + H \rightarrow C_2H_6$	$\frac{6\times10^{-11}}{1+10^{-1.915+2.69\times10^{-3}\cdot T_g-2.35\times10^{-7}\cdot T_g^2}}$	[17]
33	$C_2H_5 + H \rightarrow C_2H_4 + H_2$	3×10^{-12}	[17]
34	$C_2H_4 + H_2 \rightarrow C_2H_5 + H$	$1.7 \times 10^{-11} \cdot \exp\left(\frac{-3.43\times10^4}{T_g}\right)$	[17]
35	$C_2H_4 + H \rightarrow C_2H_3 + H_2$	$3.9 \times 10^{-22} \cdot T_g^{3.62} \cdot \exp\left(\frac{-5.67\times10^3}{T_g}\right)$	[8]
36	$C_2H_4 + H \rightarrow C_2H_5$	$k_0 = 1.3 \times 10^{-29} \cdot \exp\left(\frac{-3.8\times10^2}{T_g}\right)$<br>$k_\infty = 6.6 \times 10^{-15} \cdot T_g^{1.28} \cdot \exp\left(\frac{-6.5\times10^2}{T_g}\right)$<br>$F_c = 0.24 \cdot \exp\left(\frac{-T_g}{4\times10^1}\right)$<br>$+0.76 \cdot \exp\left(\frac{-T_g}{1.025\times10^3}\right)$	[8][a]
37	$C_2H_3 + H_2 \rightarrow C_2H_4 + H$	$1.57 \times 10^{-20} \cdot T_g^{2.56} \cdot \exp\left(\frac{-2.529\times10^3}{T_g}\right)$	[18]
38	$C_2H_3 + H \rightarrow C_2H_2 + H_2$	7×10^{-11}	[8]
39	$C_2H_3 + H \rightarrow C_2H_4$	$k_0 = 3.5 \times 10^{-27}$<br>$k_\infty = 1.6 \times 10^{-10}$<br>$F_c = 0.5$	[8][a]
40	$C_2H_2 + H_2 \rightarrow C_2H_3 + H$	$4 \times 10^{-12} \cdot \exp\left(\frac{-3.27\times10^4}{T_g}\right)$	[17]
41	$C_2H_2 + H_2 \rightarrow C_2H_4$	$5 \times 10^{-13} \cdot \exp\left(\frac{-1.96\times10^4}{T_g}\right)$	[17]
42	$C_2H_2 + H \rightarrow C_2H_3$	$k_0 = 1 \times 10^{-20} \cdot T_g^{-3.38} \cdot \exp\left(\frac{-4.26\times10^2}{T_g}\right)$<br>$k_\infty = 9.2 \times 10^{-16} \cdot T_g^{1.64} \cdot \exp\left(\frac{-1.055\times10^3}{T_g}\right)$<br>$F_c = 7.37 \times 10^{-4} \cdot T_g^{0.8}$	[8][a]
43	$C_2H_2 + H \rightarrow C_2H + H_2$	$1.67 \times 10^{-14} \cdot T_g^{1.64} \cdot \exp\left(\frac{-1.525\times10^4}{T_g}\right)$	[8]
44	$C_2H + H_2 \rightarrow C_2H_2 + H$	$3.5 \times 10^{-18} \cdot T_g^{2.32} \cdot \exp\left(\frac{-4.44\times10^2}{T_g}\right)$	[8]
45	$C_2H + H \rightarrow C_2H_2$	3×10^{-10}	[17]
46	$C + C_2H_4 \rightarrow C_2H_2 + CH_2$	1.239×10^{-11}	[19, 20]
47	$C_2H_6 + CH_3 \rightarrow C_2H_5 + CH_4$	$9.3 \times 10^{-14} \cdot \exp\left(\frac{-4.740\times10^3}{T_g}\right)$<br>$+1.4 \times 10^{-9} \cdot \exp\left(\frac{-1.120\times10^4}{T_g}\right)$	[8]
48	$C_2H_6 + CH_2 \rightarrow C_2H_5 + CH_3$	$\frac{6.5\times10^{12}}{N_A} \cdot \exp\left(\frac{-3.31\times10^4}{R\cdot T_g}\right)$	[13]
49	$C_2H_6 + CH \rightarrow C_2H_4 + CH_3$	1.3×10^{-10}	[21]
50	$C_2H_5 + CH_4 \rightarrow C_2H_6 + CH_3$	$1.43 \times 10^{-25} \cdot T_g^{4.14} \cdot \exp\left(\frac{-6.322\times10^3}{T_g}\right)$	[17]
51	$C_2H_5 + CH_3 \rightarrow C_2H_4 + CH_4$	1.5×10^{-12}	[8]
52	$C_2H_5 + CH_3 \rightarrow C_2H_6 + CH_2$	$3 \times 10^{-44} \cdot T_g^{9.0956}$	[22]
53	$C_2H_5 + CH_2 \rightarrow C_2H_4 + CH_3$	3×10^{-11}	[17]
54	$C_2H_4 + CH_3 \rightarrow C_2H_3 + CH_4$	$1 \times 10^{-16} \cdot T_g^{1.56} \cdot \exp\left(\frac{-8.37\times10^3}{T_g}\right)$	[8]
55	$C_2H_3 + CH_4 \rightarrow C_2H_4 + CH_3$	$2.4 \times 10^{-24} \cdot T_g^{4.02} \cdot \exp\left(\frac{-2.754\times10^3}{T_g}\right)$	[17]
56	$C_2H_3 + CH_3 \rightarrow C_2H_2 + CH_4$	$1.5 \times 10^{-11} \cdot \exp\left(\frac{3.850\times10^2}{T_g}\right)$	[23]
57	$C_2H_3 + CH_2 \rightarrow C_2H_2 + CH_3$	3×10^{-11}	[17]
58	$C_2H_2 + CH_3 \rightarrow C_2H + CH_4$	$3 \times 10^{-13} \cdot \exp\left(\frac{-8.7\times10^3}{T_g}\right)$	[17]

#	Reaction	Rate equation	Ref.
59	$C_2H + CH_4 \rightarrow C_2H_2 + CH_3$	$3.6 \times 10^{-14} \cdot T_g^{0.94} \cdot \exp\left(\frac{-3.28\times10^2}{T_g}\right)$	[8]
60	$C_2H + CH_2 \rightarrow C_2H_2 + CH$	3×10^{-11}	[17]
61	$C_2H_3 + C_2H_6 \rightarrow C_2H_4 + C_2H_5$	$1 \times 10^{-21} \cdot T_g^{3.3} \cdot \exp\left(\frac{-5.285\times10^3}{T_g}\right)$	[17]
62	$C_2H + C_2H_6 \rightarrow C_2H_2 + C_2H_5$	$6.75 \times 10^{-12} \cdot T_g^{0.28} \cdot \exp\left(\frac{6.2\times10^1}{T_g}\right)$	[8]
63	$C_2H_5 + C_2H_5 \rightarrow C_2H_4 + C_2H_6$	2.3×10^{-12}	[8]
64	$C_2H_4 + C_2H_5 \rightarrow C_2H_3 + C_2H_6$	$8.1 \times 10^{-31} \cdot T_g^{5.82} \cdot \exp\left(\frac{-6\times10^3}{T_g}\right)$	[8]
65	$C_2H_3 + C_2H_5 \rightarrow C_2H_2 + C_2H_6$	2.3985×10^{-11}	[24, 25]
66	$C_2H_3 + C_2H_5 \rightarrow C_2H_4 + C_2H_4$	4.42×10^{-11}	[24, 25]
67	$C_2H_2 + C_2H_5 \rightarrow C_2H + C_2H_6$	$4.5 \times 10^{-13} \cdot \exp\left(\frac{-1.18\times10^4}{T_g}\right)$	[17]
68	$C_2H + C_2H_5 \rightarrow C_2H_2 + C_2H_4$	3×10^{-12}	[17]
69	$C_2H_4 + C_2H_4 \rightarrow C_2H_3 + C_2H_5$	$8 \times 10^{-10} \cdot \exp\left(\frac{-3.6\times10^4}{T_g}\right)$	[17]
70	$C_2H_2 + C_2H_4 \rightarrow C_2H_3 + C_2H_3$	$4 \times 10^{-11} \cdot \exp\left(\frac{-3.44\times10^4}{T_g}\right)$	[17]
71	$C_2H + C_2H_4 \rightarrow C_2H_2 + C_2H_3$	$3.35 \times 10^{-18} \cdot T_g^{2.24}$	[26]
72	$C_2H_3 + C_2H_3 \rightarrow C_2H_2 + C_2H_4$	1.6×10^{-12}	[17]
73	$C_2H + C_2H_3 \rightarrow C_2H_2 + C_2H_2$	1.6×10^{-12}	[17]
74	$C_2H_2 + C_2H_2 \rightarrow C_2H + C_2H_3$	$1.6 \times 10^{-11} \cdot \exp\left(\frac{-4.25\times10^4}{T_g}\right)$	[17]
75	$C_2H_6 \rightarrow CH_3 + CH_3$	$k_0 = 2.6 \times 10^{25} \cdot T_g^{-8.37} \cdot \exp\left(\frac{-4.729\times10^4}{T_g}\right)$<br>$k_\infty = 4.5 \times 10^{21} \cdot T_g^{-1.37} \cdot \exp\left(\frac{-4.59\times10^4}{T_g}\right)$<br>$F_c = 0.38 \cdot \exp\left(\frac{-T_g}{7.3\times10^1}\right)$<br>$+0.62 \cdot \exp\left(\frac{-T_g}{1.18\times10^3}\right)$	[8][a]
76	$C_2H_6 \rightarrow C_2H_5 + H$	$k_0 = \frac{10^{4.2839\times10^1}}{n_M} \cdot T_g^{-6.431} \cdot \exp\left(\frac{-5.3938\times10^4}{T_g}\right)$<br>$k_\infty = 10^{2.0947\times10^1} \cdot T_g^{-1.228} \cdot \exp\left(\frac{-5.1439\times10^4}{T_g}\right)$<br>$F_c = 4.761 \times 10^1 \cdot \exp\left(\frac{-1.6182\times10^4}{T_g}\right)$<br>$+\exp\left(\frac{-T_g}{3.371\times10^3}\right)$	[27][a]
77	$C_2H_5 \rightarrow C_2H_4 + H$	$k_0 = 1.7 \times 10^{-6} \cdot \exp\left(\frac{-1.68\times10^4}{T_g}\right)$<br>$k_\infty = 8.2 \times 10^{13} \cdot \exp\left(\frac{-2.007\times10^4}{T_g}\right)$<br>$F_c = 0.25 \cdot \exp\left(\frac{-T_g}{9.7\times10^1}\right)$<br>$+0.75 \cdot \exp\left(\frac{-T_g}{1.379\times10^3}\right)$	[8][a]
78	$C_2H_4 \rightarrow C_2H_3 + H$	$10^{1.63\times10^1} \cdot \exp\left(\frac{-4.6\times10^5}{R\cdot T_g}\right)$	[28]
79	$C_2H_4 \rightarrow C_2H_2 + H_2$	$10^{1.29\times10^1} \cdot T_g^{0.44} \cdot \exp\left(\frac{-4.467\times10^4}{T_g}\right)$	[17]
80	$C_2H_3 \rightarrow C_2H_2 + H$	$k_0 = 4.3 \times 10^3 \cdot T_g^{-3.4} \cdot \exp\left(\frac{-1.802\times10^4}{T_g}\right)$<br>$k_\infty = 3.9 \times 10^8 \cdot T_g^{1.62} \cdot \exp\left(\frac{-1.865\times10^4}{T_g}\right)$<br>$F_c = 7.37 \times 10^{-4} \cdot T_g^{0.8}$	[8][a]
81	$C_2H_2 \rightarrow C_2H + H$	$10^{1.542\times10^1} \cdot \exp\left(\frac{-6.2445\times10^4}{T_g}\right)$	[17]
82	$O + OH \rightarrow H + O_2$	$4.33 \times 10^{-11} \cdot \left(\frac{T_g}{3.0\times10^2}\right)^{-0.5} \cdot \exp\left(\frac{-3.0\times10^1}{T_g}\right)$	[17]
83	$H + OH \rightarrow H_2 + O$	$4.1 \times 10^{-12} \cdot \frac{T_g}{3.0\times10^2} \cdot \exp\left(\frac{-3.50\times10^3}{T_g}\right)$	[29]
84	$OH + OH \rightarrow H_2O + O$	$1.02 \times 10^{-12} \cdot \left(\frac{T_g}{3.0\times10^2}\right)^{1.4} \cdot \exp\left(\frac{2.0\times10^2}{T_g}\right)$	[17]
85	$OH + OH \rightarrow H + HO_2$	$2 \times 10^{-11} \cdot \exp\left(\frac{-2.020\times10^4}{T_g}\right)$	[29]
86	$OH + OH \rightarrow H_2 + O_2$	$1.82 \times 10^{-13} \cdot T_g^{0.51} \cdot \exp\left(\frac{-2.54\times10^4}{T_g}\right)$	[30]

#	Reaction	Rate equation	Ref.
87	$M + OH \rightarrow M + H + O$	$4.7 \times 10^{-8} \cdot \left(\frac{T_g}{3.0\times10^2}\right)^{-1.0} \cdot \exp\left(\frac{-5.0830\times10^4}{T_g}\right)$	[29]
88	$H_2 + OH \rightarrow H + H_2O$	$3.6 \times 10^{-16} \cdot T_g^{1.52} \cdot \exp\left(\frac{-1.74\times10^3}{T_g}\right)$	[8]
89	$O_2 + OH \rightarrow H + O_3$	$2.7 \times 10^{-13} \cdot \left(\frac{T_g}{3.0\times10^2}\right)^{1.44} \cdot \exp\left(\frac{-3.860\times10^4}{T_g}\right)$	[29]
90	$O_2 + OH \rightarrow HO_2 + O$	$2.2 \times 10^{-11} \cdot \exp\left(\frac{-2.820\times10^4}{T_g}\right)$	[29]
91	$O_3 + OH \rightarrow HO_2 + O_2$	$1.69 \times 10^{-12} \cdot \exp\left(\frac{-9.410\times10^2}{T_g}\right)$	[31]
92	$H_2O + OH \rightarrow H + H_2O_2$	$4 \times 10^{-10} \cdot \exp\left(\frac{-4.050\times10^4}{T_g}\right)$	[29]
93	$HO_2 + OH \rightarrow H_2O + O_2$	$8.05 \times 10^{-11} \cdot \left(\frac{T_g}{3.0\times10^2}\right)^{-1.0}$	[17]
94	$HO_2 + OH \rightarrow H_2O_2 + O$	$1.5 \times 10^{-12} \cdot \left(\frac{T_g}{3.0\times10^2}\right)^{0.5} \cdot \exp\left(\frac{-1.060\times10^4}{T_g}\right)$	[29]
95	$H_2O_2 + OH \rightarrow H_2O + HO_2$	$2.9 \times 10^{-12} \cdot \exp\left(\frac{-1.60\times10^2}{T_g}\right)$	[17]
96	$H + HO_2 \rightarrow H_2 + O_2$	$1.1 \times 10^{-10} \cdot \exp\left(\frac{-1.070\times10^3}{T_g}\right)$	[17]
97	$H + HO_2 \rightarrow OH + OH$	$2.8 \times 10^{-10} \cdot \exp\left(\frac{-4.40\times10^2}{T_g}\right)$	[17]
98	$H + HO_2 \rightarrow H_2O + O$	$5 \times 10^{-11} \cdot \exp\left(\frac{-8.660\times10^2}{T_g}\right)$	[32]
99	$H_2O + HO_2 \rightarrow H_2O_2 + OH$	$3 \times 10^{-11} \cdot \exp\left(\frac{-1.510\times10^4}{T_g}\right)$	[29]
100	$H_2 + HO_2 \rightarrow H_2O + OH$	$1.1 \times 10^{-12} \cdot \exp\left(\frac{-9.40\times10^3}{T_g}\right)$	[29]
101	$H_2 + HO_2 \rightarrow H + H_2O_2$	$1 \times 10^{-12} \cdot \exp\left(\frac{-9.30\times10^3}{T_g}\right)$	[29]
102	$HO_2 + HO_2 \rightarrow H_2O_2 + O_2$	$2.2 \times 10^{-13} \cdot \exp\left(\frac{6.0\times10^2}{T_g}\right)$	[33]
103	$HO_2 + O \rightarrow O_2 + OH$	$2.9 \times 10^{-11} \cdot \exp\left(\frac{2.0\times10^2}{T_g}\right)$	[17]
104	$HO_2 + O_2 \rightarrow O_3 + OH$	1.5×10^{-15}	[29]
105	$H + H_2O_2 \rightarrow H_2 + HO_2$	$8 \times 10^{-11} \cdot \exp\left(\frac{-4.0\times10^3}{T_g}\right)$	[17]
106	$H + H_2O_2 \rightarrow H_2O + OH$	$4 \times 10^{-11} \cdot \exp\left(\frac{-2.0\times10^3}{T_g}\right)$	[17]
107	$H_2O_2 + O \rightarrow HO_2 + OH$	$1.44 \times 10^{-12} \cdot \left(\frac{T_g}{3.0\times10^2}\right)^{2.0} \cdot \exp\left(\frac{-2.0\times10^3}{T_g}\right)$	[17]
108	$H_2O_2 \rightarrow OH + OH$	$k_0 = 3.8 \times 10^{-8} \cdot \exp\left(\frac{-2.196\times10^4}{T_g}\right)$<br>$k_\infty = 3 \times 10^{14} \cdot \exp\left(\frac{-2.44\times10^4}{T_g}\right)$<br>$F_c = 0.5$	[8][a]
109	$H_2O_2 + O_2 \rightarrow HO_2 + HO_2$	$5 \times 10^{-11} \cdot \exp\left(\frac{-2.160\times10^4}{T_g}\right)$	[29]
110	$H_2O + O \rightarrow OH + OH$	$7.6 \times 10^{-15} \cdot T_g^{1.3} \cdot \exp\left(\frac{-8.6\times10^3}{T_g}\right)$	[17]
111	$M + H_2O \rightarrow M + H + OH$	$5.9 \times 10^{-7} \cdot \left(\frac{T_g}{3.0\times10^2}\right)^{-2.2} \cdot \exp\left(\frac{-5.90\times10^4}{T_g}\right)$	[29]
112	$H + H_2O \rightarrow H_2 + OH$	$7.5 \times 10^{-16} \cdot T_g^{1.6} \cdot \exp\left(\frac{-9.03\times10^3}{T_g}\right)$	[8]
113	$H_2O + OH \rightarrow H_2 + HO_2$	$1.4 \times 10^{-13} \cdot \exp\left(\frac{-3.610\times10^4}{T_g}\right)$	[29]
114	$H_2O + O \rightarrow H + HO_2$	$2.8 \times 10^{-12} \cdot \left(\frac{T_g}{3.0\times10^2}\right)^{0.37} \cdot \exp\left(\frac{-2.87430\times10^4}{T_g}\right)$	[29]
115	$H_2O + O_2 \rightarrow H_2O_2 + O$	$9.8 \times 10^{-8} \cdot \left(\frac{T_g}{3.0\times10^2}\right)^{0.5} \cdot \exp\left(\frac{-4.480\times10^4}{T_g}\right)$	[29]
116	$H_2O + O_2 \rightarrow HO_2 + OH$	$4.3 \times 10^{-12} \cdot \left(\frac{T_g}{3.0\times10^2}\right)^{0.5} \cdot \exp\left(\frac{-3.660\times10^4}{T_g}\right)$	[29]
117	$M + H + O \rightarrow M + OH$	$4.33 \times 10^{-32} \cdot \left(\frac{T_g}{3\times10^2}\right)^{-1}$	[17]
118	$H + O_2 \rightarrow O + OH$	$1.62 \times 10^{-10} \cdot \exp\left(\frac{-7.4740\times10^3}{T_g}\right)$	[32]
119	$M + H + O_2 \rightarrow M + HO_2$	$3.33 \times 10^{-31} \cdot \left(\frac{T_g}{3.0\times10^2}\right)^{-1}$	[8]
120	$H + O_3 \rightarrow HO_2 + O$	7.76×10^{-13}	[34]
121	$H + O_3 \rightarrow O_2 + OH$	2.36×10^{-11}	[34]
122	$H_2 + O_3 \rightarrow HO_2 + OH$	$1 \times 10^{-13} \cdot \exp\left(\frac{-1.0\times10^4}{T_g}\right)$	[29]

#	Reaction	Rate equation	Ref.
123	$H_2 + O_2 \rightarrow H + HO_2$	$3.2 \times 10^{-11} \cdot \exp\left(\frac{-2.410\times10^4}{T_g}\right)$	[29]
124	$H_2 + O \rightarrow H + OH$	$9 \times 10^{-12} \cdot \frac{T_g}{3.0\times10^2} \cdot \exp\left(\frac{-4.480\times10^3}{T_g}\right)$	[29]
125	$C + OH \rightarrow CO + H$	$\frac{5\times10^{13}}{N_A}$	[35]
126	$CO_2 + H \rightarrow CO + OH$	$4.7 \times 10^{-10} \cdot \exp\left(\frac{-1.3915\times10^4}{T_g}\right)$	[8]
127	$CO + H \rightarrow HCO$	$2 \times 10^{-35} \cdot T_g^{0.2} \cdot n_M$	[8]
128	$CO + OH \rightarrow CO_2 + H$	$\frac{3.3\times10^6}{N_A} \cdot T_g^{1.55} \cdot \exp\left(\frac{4.02\times10^2}{T_g}\right)$	[36]
129	$CO + HO_2 \rightarrow CO_2 + OH$	$\frac{5.8\times10^{13}}{N_A} \cdot \exp\left(\frac{-2.293\times10^4 \cdot 4.184}{R \cdot T_g}\right)$	[37]
130	$CO + H_2O_2 \rightarrow COOH + OH$	$\frac{3.6\times10^4}{N_A} \cdot T_g^{2.5} \cdot \exp\left(\frac{-1.4425\times10^4}{T_g}\right)$	[38]
131	$CH_4 + O \rightarrow CH_3 + OH$	$7.3 \times 10^{-19} \cdot T_g^{2.5} \cdot \exp\left(\frac{-3.31\times10^3}{T_g}\right)$	[8]
132	$CH_4 + O_2 \rightarrow CH_3 + HO_2$	$8.1 \times 10^{-19} \cdot T_g^{2.5} \cdot \exp\left(\frac{-2.637\times10^4}{T_g}\right)$	[8]
133	$CH_4 + O_2 \rightarrow CH_3OO + H$	$\frac{4.3\times10^{13}}{N_A} \cdot \left(\frac{T_g}{1\times10^3}\right)^{1.96} \cdot \exp\left(\frac{-8.73\times10^1 \cdot 4.184\times10^3}{R \cdot T_g}\right)$	[39]
134	$CH_3 + O \rightarrow H + HCHO$	1.12×10^{-10}	[8]
135	$CH_3 + O_2 \rightarrow HCHO + OH$	$3.7 \times 10^{-12} \cdot \exp\left(\frac{-1.114\times10^4}{T_g}\right)$	[8]
136	$CH_3 + O_2 \rightarrow CH_3O + O$	$3.5 \times 10^{-11} \cdot \exp\left(\frac{-1.634\times10^4}{T_g}\right)$	[8]
137	$CH_3 + O_2 \rightarrow CH_3OO$	$1.3 \times 10^{-15} \cdot T_g^{1.2}$	[8]
138	$CH_2 + O \rightarrow CO + H_2$	$0.4 \cdot 3.4 \times 10^{-10} \cdot \exp\left(\frac{-2.7\times10^2}{T_g}\right)$	[8]
139	$CH_2 + O_2 \rightarrow HCHO + O$	$\frac{4\times10^{10}}{N_A}$	[40]
140	$CH_2 + O_2 \rightarrow CO + H_2O$	4.2×10^{-13}	[17]
141	$CH + O \rightarrow CO + H$	6.6×10^{-11}	[8]
142	$CH + O_2 \rightarrow CO_2 + H$	4.2×10^{-11}	[8]
143	$CH + O_2 \rightarrow CO + OH$	2.8×10^{-11}	[8]
144	$CH + O_2 \rightarrow HCO + O$	2.8×10^{-11}	[8]
145	$CH_3 + CO \rightarrow CH_3CO$	$k_0 = 1.6 \times 10^{-37} \cdot T_g^{1.05} \cdot \exp\left(\frac{-1.3\times10^3}{T_g}\right)$<br>$k_\infty = 3.1 \times 10^{-16} \cdot T_g^{1.05} \cdot \exp\left(\frac{-2.85\times10^3}{T_g}\right)$<br>$F_c = 0.5$	[8][a]
146	$CH_2 + CO_2 \rightarrow CO + HCHO$	3.9×10^{-14}	[17]
147	$CH_2 + CO \rightarrow CH_2CO$	1×10^{-15}	[17]
148	$CH + CO_2 \rightarrow CO + HCO$	$0.5 \cdot 1.06 \times 10^{-16} \cdot T_g^{1.51} \cdot \exp\left(\frac{3.6\times10^2}{T_g}\right)$	[8]
149	$CH + CO \rightarrow HCCO$	$k_0 = 6.3 \times 10^{-24} \cdot T_g^{-2.5}$<br>$k_\infty = 1.7 \times 10^{-9} \cdot T_g^{-0.4}$<br>$F_c = 0.6$	[8][a]
150	$CH_4 + OH \rightarrow CH_3 + H_2O$	$1.66 \times 10^{-18} \cdot T_g^{2.182} \cdot \exp\left(\frac{-1.231\times10^3}{T_g}\right)$	[41]
151	$CH_4 + HO_2 \rightarrow CH_3 + H_2O_2$	$7.8 \times 10^{-20} \cdot T_g^{2.5} \cdot \exp\left(\frac{-1.057\times10^4}{T_g}\right)$	[8]
152	$CH_3 + OH \rightarrow CH_3OH$	$k_0 = 1.06 \times 10^{-10} \cdot T_g^{-6.21} \cdot \exp\left(\frac{-6.71\times10^2}{T_g}\right)$<br>$k_\infty = 7.2 \times 10^{-9} \cdot T_g^{-0.79}$<br>$F_c = 0.75 \cdot \exp\left(\frac{-T_g}{2.1\times10^2}\right)$<br>$+0.25 \cdot \exp\left(\frac{-T_g}{1.434\times10^3}\right)$	[8][a]

#	Reaction	Rate equation	Ref.
153	$CH_3 + OH \rightarrow CH_2 + H_2O$	$\frac{k}{n_M}$ $k_0 = 1.8 \times 10^{-8} \cdot T_g^{-0.91} \cdot \exp\left(\frac{-2.75\times10^2}{T_g}\right)$ $k_\infty = 6.4 \times 10^{-8} \cdot T_g^{5.8} \cdot \exp\left(\frac{4.85\times10^2}{T_g}\right)$ $F_c = 0.664 \cdot \exp\left(\frac{-T_g}{3.569\times10^3}\right)$ $+0.336 \cdot \exp\left(\frac{-T_g}{1.08\times10^2}\right)$ $+\exp\left(\frac{-3.24\times10^3}{T_g}\right)$	[8][a]
154	$CH_3 + OH \rightarrow CH_2OH + H$	$1.2 \times 10^{-12} \cdot \exp\left(\frac{-2.76\times10^3}{T_g}\right)$	[8]
155	$CH_3 + OH \rightarrow CH_3O + H$	$2 \times 10^{-14} \cdot \exp\left(\frac{-6.99\times10^3}{T_g}\right)$	[8]
156	$CH_3 + OH \rightarrow H_2 + HCHO$	$5.3 \times 10^{-15} \cdot \exp\left(\frac{-2.53\times10^3}{T_g}\right)$	[8]
157	$CH_3 + OH \rightarrow CH_4 + O$	$1.16 \times 10^{-19} \cdot T_g^{2.2} \cdot \exp\left(\frac{-2.24\times10^3}{T_g}\right)$	[42]
158	$CH_3 + H_2O \rightarrow CH_4 + OH$	$8 \times 10^{-22} \cdot T_g^{2.9} \cdot \exp\left(\frac{-7.48\times10^3}{T_g}\right)$	[43]
159	$CH_3 + HO_2 \rightarrow CH_3O + OH$	3×10^{-11}	[8]
160	$CH_3 + HO_2 \rightarrow CH_4 + O_2$	6×10^{-12}	[17]
161	$CH_3 + H_2O_2 \rightarrow CH_4 + HO_2$	$2 \times 10^{-14} \cdot \exp\left(\frac{3.0\times10^2}{T_g}\right)$	[17]
162	$CH_2 + OH \rightarrow H + HCHO$	5×10^{-11}	[17]
163	$CH_2 + H_2O \rightarrow CH_3 + OH$	1×10^{-16}	[17]
164	$CH_2 + HO_2 \rightarrow HCHO + OH$	3×10^{-11}	[17]
165	$CH_2 + H_2O_2 \rightarrow CH_3 + HO_2$	1×10^{-14}	[17]
166	$CH + OH \rightarrow C + H_2O$	$\frac{4\times10^7}{N_A} \cdot T_g^2 \cdot \exp\left(\frac{-3\times10^3\cdot4.184}{R\cdot T_g}\right)$	[37]
167	$CH + OH \rightarrow H + HCO$	$\frac{3\times10^{13}}{N_A}$	[37]
168	$CH + H_2O \rightarrow H + HCHO$	$\frac{8.5\times10^8}{N_A} \cdot T_g^{1.144} \cdot \exp\left(\frac{2.051\times10^3\cdot4.184}{R\cdot T_g}\right)$	[35]
169	$CO + COOH \rightarrow CO_2 + HCO$	1×10^{-14}	[44]
170	$CH_3O + CO \rightarrow CH_3 + CO_2$	$2.6 \times 10^{-11} \cdot \exp\left(\frac{-5.94\times10^3}{T_g}\right)$	[17]
171	$CH_3O + CO \rightarrow HCHO + HCO$	5.23×10^{-15}	[45]
172	$CH_3OO + CO \rightarrow CH_3O + CO_2$	7×10^{-18}	[46]
173	$H + HCO \rightarrow CO + H_2$	1.5×10^{-10}	[8]
174	$H + HCO \rightarrow CH_2 + O$	$\frac{3.98107171\times10^{13}}{N_A} \cdot \exp\left(\frac{-4.29\times10^5}{R\cdot T_g}\right)$	[47]
175	$H + HCHO \rightarrow H_2 + HCO$	$3.34 \times 10^{-23} \cdot T_g^{-3.81} \cdot \exp\left(\frac{-2.02\times10^2}{T_g}\right)$	[8]
176	$H + HCHO \rightarrow CH_3O$	$\frac{2.4\times10^{13}}{N_A} \cdot \exp\left(\frac{-4.11\times10^3\cdot4.184}{T_g}\right)$	[48]
177	$CH_3O + H \rightarrow H_2 + HCHO$	3.3×10^{-11}	[17]
178	$CH_3O + H \rightarrow CH_3OH$	$3.4 \times 10^{-10} \cdot \left(\frac{T_g}{3\times10^2}\right)^{0.33}$	[49]
179	$CH_3O + H_2 \rightarrow CH_3OH + H$	$1.7 \times 10^{-15} \cdot \left(\frac{T_g}{3\times10^2}\right)^4 \cdot \exp\left(\frac{-2.47\times10^3}{T_g}\right)$	[50]
180	$CH_2OH + H \rightarrow H_2 + HCHO$	1×10^{-11}	[51]
181	$CH_2OH + H \rightarrow CH_3 + OH$	1.6×10^{-10}	[51]
182	$CH_2OH + H_2 \rightarrow CH_3OH + H$	$1.12 \times 10^{-18} \cdot T_g^2 \cdot \exp\left(\frac{-6.722\times10^3}{T_g}\right)$	[51]
183	$CH_3OH + H \rightarrow CH_2OH + H_2$	$5.7 \times 10^{-15} \cdot T_g^{1.24} \cdot \exp\left(\frac{-2.26\times10^3}{T_g}\right)$	[8]
184	$CH_3OH + H \rightarrow CH_3 + H_2O$	$\frac{2\times10^{12}}{N_A} \cdot \exp\left(\frac{-5.3\cdot4.184\times10^3}{R\cdot T_g}\right)$	[52]
185	$CH_3OO + H \rightarrow CH_4 + O_2$	$\frac{4.02\times10^{13}}{N_A} \cdot \left(\frac{T_g}{1\times10^3}\right)^{1.02} \cdot \exp\left(\frac{-1.66\times10^1\cdot4.184\times10^3}{R\cdot T_g}\right)$	[39]
186	$CH_3OO + H \rightarrow CH_3O + OH$	1.6×10^{-10}	[17]

#	Reaction	Rate equation	Ref.
187	$CH_3OO + H_2 \rightarrow CH_3OOH + H$	$5 \times 10^{-11} \cdot \exp\left(\frac{-1.31\times10^4}{T_g}\right)$	[17]
188	$HCO + OH \rightarrow CO + H_2O$	1.8×10^{-10}	[8]
189	$H_2O + HCO \rightarrow HCHO + OH$	$3.9 \times 10^{-16} \cdot T_g^{1.35} \cdot \exp\left(\frac{-1.3146\times10^4}{T_g}\right)$	[17]
190	$H_2O_2 + HCO \rightarrow HCHO + HO_2$	$1.7 \times 10^{-13} \cdot \exp\left(\frac{-3.486\times10^3}{T_g}\right)$	[17]
191	$HCHO + OH \rightarrow H_2O + HCO$	$2.31 \times 10^{-11} \cdot \exp\left(\frac{-3.04\times10^2}{T_g}\right)$	[8]
192	$HCHO + OH \rightarrow H + HCOOH$	2×10^{-13}	[53]
193	$HCHO + HO_2 \rightarrow H_2O_2 + HCO$	$6.8 \times 10^{-20} \cdot T_g^{2.5} \cdot \exp\left(\frac{-5.14\times10^3}{T_g}\right)$	[8]
194	$HCHO + HO_2 \rightarrow CH_2OH + O_2$	$\frac{3.38844156\times10^{12}}{N_A} \cdot \exp\left(\frac{-8\times10^4}{R\cdot T_g}\right)$	[47]
195	$HCOOH + OH \rightarrow COOH + H_2O$	$\frac{5.93\times10^8\cdot1\times10^3}{N_A} \cdot \exp\left(\frac{-1.036\times10^3}{T_g}\right)$	[54]
196	$CH_3O + OH \rightarrow H_2O + HCHO$	3×10^{-11}	[17]
197	$CH_3O + HO_2 \rightarrow H_2O_2 + HCHO$	5×10^{-13}	[17]
198	$CH_3O + HO_2 \rightarrow CH_3OH + O_2$	4.7×10^{-11}	[55]
199	$CH_2OH + OH \rightarrow H_2O + HCHO$	4×10^{-11}	[51]
200	$CH_2OH + H_2O \rightarrow CH_3OH + OH$	$\frac{1.54881662\times10^{14}}{N_A} \cdot \exp\left(\frac{-1.1\times10^5}{R\cdot T_g}\right)$	[47]
201	$CH_2OH + HO_2 \rightarrow H_2O_2 + HCHO$	$\frac{1.3\times10^6\cdot1\times10^3}{N_A} \cdot \left(\frac{T_g}{2.98\times10^2}\right)^{5.31} \cdot \exp\left(\frac{-6.01\times10^4}{R\cdot T_g}\right)$	[56]
202	$CH_2OH + HO_2 \rightarrow CH_3OH + O_2$	$\frac{5.7\times10^4\cdot1\times10^3}{N_A} \cdot \left(\frac{T_g}{2.98\times10^2}\right)^{3.2} \cdot \exp\left(\frac{-6.8\times10^3}{R\cdot T_g}\right)$	[56]
203	$CH_2OH + HO_2 \rightarrow H_2O + HCOOH$	$\frac{3.6\times10^9\cdot1\times10^3}{N_A} \cdot T_g^{0.12} \cdot \exp\left(\frac{-1.9\times10^3}{R\cdot T_g}\right)$	[56]
204	$CH_2OH + H_2O_2 \rightarrow CH_3OH + HO_2$	$5 \times 10^{-15} \cdot \exp\left(\frac{-1.3\times10^3}{T_g}\right)$	[51]
205	$CH_3OH + HO_2 \rightarrow CH_2OH + H_2O_2$	$5.41 \times 10^{-11} \cdot \exp\left(\frac{-9.2\times10^3}{T_g}\right)$	[57]
206	$CH_3OH + HO_2 \rightarrow CH_3O + H_2O_2$	$2.02 \times 10^{-12} \cdot \exp\left(\frac{-1.01\times10^4}{T_g}\right)$	[57]
207	$CH_3OOH + OH \rightarrow CH_3OO + H_2O$	$1.8 \times 10^{-12} \cdot \exp\left(\frac{2.2\times10^2}{T_g}\right)$	[8]
208	$CH_3OO + OH \rightarrow CH_3OH + O_2$	1×10^{-10}	[17]
209	$CH_3OO + HO_2 \rightarrow CH_3OOH + O_2$	$0.9 \cdot 4.2 \times 10^{-13} \cdot \exp\left(\frac{7.5\times10^2}{T_g}\right)$	[8]
210	$CH_3OO + H_2O_2 \rightarrow CH_3OOH + HO_2$	$4 \times 10^{-12} \cdot \exp\left(\frac{-5\times10^3}{T_g}\right)$	[17]
211	$HCO + O \rightarrow CO + OH$	5×10^{-11}	[17]
212	$HCO + O \rightarrow CO_2 + H$	5×10^{-11}	[17]
213	$HCO + O_2 \rightarrow CO + HO_2$	$4.5 \times 10^{-14} \cdot T_g^{0.68} \cdot \exp\left(\frac{2.36\times10^2}{T_g}\right)$	[8]
214	$HCHO + O \rightarrow HCO + OH$	$6.9 \times 10^{-13} \cdot T_g^{0.57} \cdot \exp\left(\frac{-1.39\times10^3}{T_g}\right)$	[8]
215	$HCHO + O_2 \rightarrow HCO + HO_2$	$4.05 \times 10^{-19} \cdot T_g^{2.5} \cdot \exp\left(\frac{-1.835\times10^4}{T_g}\right)$	[8]
216	$CH_3O + O \rightarrow CH_3 + O_2$	1.875×10^{-11}	[8]
217	$CH_3O + O \rightarrow HCHO + OH$	6.25×10^{-12}	[8]
218	$CH_3O + O_2 \rightarrow HCHO + HO_2$	$3.6 \times 10^{-14} \cdot \exp\left(\frac{-8.8\times10^2}{T_g}\right)$	[8]
219	$CH_2OH + O_2 \rightarrow HCHO + HO_2$	$4.8 \times 10^{-8} \cdot T_g^{-1.5}$ $+1.2 \times 10^{-10} \cdot \exp\left(\frac{-1.88\times10^3}{T_g}\right)$	[8]
220	$CH_3OH + O \rightarrow CH_2OH + OH$	$4.1 \times 10^{-11} \cdot \exp\left(\frac{-2.67\times10^3}{T_g}\right)$	[8]
221	$CH_3OH + O_2 \rightarrow CH_2OH + HO_2$	$3.4 \times 10^{-11} \cdot \exp\left(\frac{-2.26\times10^4}{T_g}\right)$	[51]
222	$CH_3OO + O \rightarrow CH_3O + O_2$	6×10^{-11}	[17]
223	$CH_4 + HCO \rightarrow CH_3 + HCHO$	$1.21 \times 10^{-20} \cdot T_g^{2.85} \cdot \exp\left(\frac{-1.133\times10^4}{T_g}\right)$	[17]
224	$CH_3 + HCO \rightarrow CH_4 + CO$	2×10^{-10}	[17]

#	Reaction	Rate equation	Ref.
225	$CH_3 + HCO \to CH_3CHO$	3×10^{-11}	[17]
226	$CH_2 + HCO \to CH_3 + CO$	3×10^{-11}	[17]
227	$CH_3 + COOH \to CH_2CO + H_2O$	$\left(1.52 + 1.95 \times 10^{-4} \cdot T_g\right) \cdot 3.24 \times 10^{-11} \cdot T_g^{0.1024}$	[58]
228	$CH_3 + COOH \to CH_4 + CO_2$	$3.24 \times 10^{-11} \cdot T_g^{0.1024}$	[58]
229	$CH_3 + HCHO \to CH_3CH_2O$	$\frac{3\times10^{11}}{N_A} \cdot \exp\left(\frac{-6.336\times10^3\cdot4.186}{R\cdot T_g}\right)$	[48]
230	$CH_3 + HCHO \to CH_4 + HCO$	$5.3 \times 10^{-23} \cdot T_g^{3.36} \cdot \exp\left(\frac{-2.17\times10^3}{T_g}\right)$	[8]
231	$CH_2 + HCHO \to CH_3 + HCO$	1×10^{-14}	[17]
232	$CH + HCHO \to CH_2CO + H$	$7.62 \times 10^{-10} \cdot T_g^{-0.32} \cdot \exp\left(\frac{3.86\times10^2}{T_g}\right)$	[59]
233	$CH_3O + CH_4 \to CH_3 + CH_3OH$	$2.6 \times 10^{-13} \cdot \exp\left(\frac{-4.45\times10^3}{T_g}\right)$	[17]
234	$CH_3 + CH_3O \to CH_4 + HCHO$	4×10^{-11}	[17]
235	$CH_2 + CH_3O \to CH_3 + HCHO$	3×10^{-11}	[17]
236	$CH_2OH + CH_4 \to CH_3 + CH_3OH$	$3.6 \times 10^{-23} \cdot T_g^{3.1} \cdot \exp\left(\frac{-8.166\times10^3}{T_g}\right)$	[51]
237	$CH_2OH + CH_3 \to CH_3CH_2OH$	2×10^{-11}	[51]
238	$CH_2OH + CH_3 \to CH_4 + HCHO$	4×10^{-12}	[51]
239	$CH_2 + CH_2OH \to C_2H_4 + OH$	4×10^{-11}	[51]
240	$CH_2 + CH_2OH \to CH_3 + HCHO$	2×10^{-12}	[51]
241	$CH_3 + CH_3OH \to CH_2OH + CH_4$	$0.33 \cdot 5 \times 10^{-23} \cdot T_g^{3.45} \cdot \exp\left(\frac{-4.02\times10^3}{T_g}\right)$	[8]
242	$CH_3 + CH_3OH \to CH_3O + CH_4$	$0.67 \cdot 5 \times 10^{-23} \cdot T_g^{3.45} \cdot \exp\left(\frac{-4.02\times10^3}{T_g}\right)$	[8]
243	$CH_2 + CH_3OH \to CH_2OH + CH_3$	$5.3 \times 10^{-23} \cdot T_g^{3.2} \cdot \exp\left(\frac{-3.609\times10^3}{T_g}\right)$	[51]
244	$CH_2 + CH_3OH \to CH_3 + CH_3O$	$2.4 \times 10^{-23} \cdot T_g^{3.1} \cdot \exp\left(\frac{-3.49\times10^3}{T_g}\right)$	[51]
245	$CH_3OO + CH_4 \to CH_3 + CH_3OOH$	$3 \times 10^{-13} \cdot \exp\left(\frac{-9.3\times10^3}{T_g}\right)$	[17]
246	$CH_3 + CH_3OO \to CH_3O + CH_3O$	4×10^{-11}	[17]
247	$CH_2 + CH_3OO \to CH_3O + HCHO$	3×10^{-11}	[17]
248	$CH_2 + CH_3OO \to C_2H_5 + O_2$	3×10^{-11}	[17]
249	$HCO + HCO \to CO + HCHO$	4.265×10^{-11}	[8]
250	$CH_3O + HCO \to CH_3OH + CO$	1.5×10^{-10}	[17]
251	$CH_2OH + HCO \to CH_3OH + CO$	2×10^{-10}	[51]
252	$CH_2OH + HCO \to HCHO + HCHO$	3×10^{-10}	[51]
253	$CH_3OH + HCO \to CH_2OH + HCHO$	$1.6 \times 10^{-20} \cdot T_g^{2.9} \cdot \exp\left(\frac{-6.596\times10^3}{T_g}\right)$	[51]
254	$CH_3OH + HCO \to CH_3O + HCHO$	$1.6 \times 10^{-22} \cdot T_g^{2.9} \cdot \exp\left(\frac{-6.596\times10^3}{T_g}\right)$	[51]
255	$CH_3O + HCHO \to CH_3OH + HCO$	$1.7 \times 10^{-13} \cdot \exp\left(\frac{-1.5\times10^3}{T_g}\right)$	[17]
256	$CH_2OH + HCHO \to CH_3OH + HCO$	$9.1 \times 10^{-21} \cdot T_g^{2.8} \cdot \exp\left(\frac{-2.95\times10^3}{T_g}\right)$	[51]
257	$CH_3O + CH_3O \to CH_3OH + HCHO$	1×10^{-10}	[17]
258	$CH_2OH + CH_3O \to CH_3OH + HCHO$	4×10^{-11}	[51]
259	$CH_3O + CH_3OH \to CH_2OH + CH_3OH$	$5 \times 10^{-13} \cdot \exp\left(\frac{-2.05\times10^3}{T_g}\right)$	[51]
260	$CH_2OH + CH_2OH \to CH_3OH + HCHO$	8×10^{-12}	[51]
261	$CH_2OH + CH_3OH \to CH_3O + CH_3OH$	$1.3 \times 10^{-14} \cdot \exp\left(\frac{-6.07\times10^3}{T_g}\right)$	[51]
262	$CH_3O + CH_3OO \to CH_3OOH + HCHO$	5×10^{-13}	[17]
263	$CH_3OH + CH_3OO \to CH_2OH + CH_3OOH$	$3.421 \times 10^{-33} \cdot T_g^{6.2} \cdot \exp\left(\frac{-2.9826\times10^4}{R\cdot T_g}\right)$	[60]

#	Reaction	Rate equation	Ref.
264	$CH_3OH + CH_3OO \rightarrow CH_3O + CH_3OOH$	$1.318 \times 10^{-27} \cdot T_g^{4.71} \cdot \exp\left(\frac{-5.6739\times10^4}{R\cdot T_g}\right)$	[60]
265	$CH_2OH + CH_3OO \rightarrow CH_3OOH + HCHO$	$1.047 \times 10^{-24} \cdot T_g^{2.69} \cdot \exp\left(\frac{1.4344\times10^4}{R\cdot T_g}\right)$	[60]
266	$CH_2OH + CH_3OO \rightarrow CH_3OH + HCOOH$	$3.89 \times 10^{-24} \cdot T_g^{2.74} \cdot \exp\left(\frac{1.4922\times10^4}{R\cdot T_g}\right)$	[60]
267	$CH_3OO + HCHO \rightarrow CH_3OOH + HCO$	$3.3 \times 10^{-12} \cdot \exp\left(\frac{-5.87\times10^3}{T_g}\right)$	[17]
268	$C_2H_6 + OH \rightarrow C_2H_5 + H_2O$	$1.52 \times 10^{-17} \cdot T_g^{2} \cdot \exp\left(\frac{-5\times10^2}{T_g}\right)$	[8]
269	$C_2H_6 + HO_2 \rightarrow C_2H_5 + H_2O_2$	$1.83 \times 10^{-19} \cdot T_g^{2.5} \cdot \exp\left(\frac{-8.48\times10^3}{T_g}\right)$	[8]
270	$C_2H_5 + OH \rightarrow C_2H_6 + O$	$1.7 \times 10^{-40} \cdot T_g^{8.8} \cdot \exp\left(\frac{-2.5\times10^2}{T_g}\right)$	[42]
271	$C_2H_5 + OH \rightarrow C_2H_4 + H_2O$	4×10^{-11}	[17]
272	$C_2H_5 + H_2O \rightarrow C_2H_6 + OH$	$5.6 \times 10^{-18} \cdot T_g^{1.44} \cdot \exp\left(\frac{-1.015\times10^4}{T_g}\right)$	[17]
273	$C_2H_5 + HO_2 \rightarrow C_2H_6 + O_2$	5×10^{-13}	[17]
274	$C_2H_5 + HO_2 \rightarrow C_2H_4 + H_2O_2$	5×10^{-13}	[17]
275	$C_2H_5 + H_2O_2 \rightarrow C_2H_6 + HO_2$	$1.45 \times 10^{-14} \cdot \exp\left(\frac{-4.9\times10^2}{T_g}\right)$	[17]
276	$C_2H_4 + OH \rightarrow CH_3 + HCHO$	$\frac{1}{3} \cdot 3.4 \times 10^{-11} \cdot \exp\left(\frac{-2.99\times10^3}{T_g}\right)$	[8]
277	$C_2H_4 + OH \rightarrow CH_3CHO + H$	$\frac{1}{3} \cdot 3.4 \times 10^{-11} \cdot \exp\left(\frac{-2.99\times10^3}{T_g}\right)$	[8]
278	$C_2H_4 + OH \rightarrow CH_2CH_2OH$	$1.92 \times 10^{-18} \cdot T_g^{2.03} \cdot \exp\left(\frac{7.97\times10^3}{R\cdot T_g}\right)$	[61]
279	$C_2H_4 + HO_2 \rightarrow C_2H_5 + O_2$	$1 \times 10^{-13} \cdot T_g^{0.07} \cdot \exp\left(\frac{-6.58\times10^3}{T_g}\right)$	[8]
280	$C_2H_3 + OH \rightarrow CH_3 + HCO$	$1.09 \times 10^{-5} \cdot T_g^{-1.85} \cdot \exp\left(\frac{-5.01\times10^2}{T_g}\right)$	[62]
281	$C_2H_3 + OH \rightarrow CH_3CO + H$	$9.42 \times 10^{-9} \cdot T_g^{-1.014} \cdot \exp\left(\frac{-1.95\times10^2}{T_g}\right)$	[62]
282	$C_2H_3 + OH \rightarrow C_2H_2 + H_2O$	$3.96 \times 10^{-13} \cdot T_g^{0.081} \cdot \exp\left(\frac{1.91\times10^2}{T_g}\right)$	[62]
283	$C_2H_3 + OH \rightarrow CH_2CO + H_2$	$1.26 \times 10^{-8} \cdot T_g^{-1.517} \cdot \exp\left(\frac{-3.63\times10^2}{T_g}\right)$	[62]
284	$C_2H_3 + OH \rightarrow CH_4 + CO$	$1.32 \times 10^{-8} \cdot T_g^{-1.328} \cdot \exp\left(\frac{-2.98\times10^2}{T_g}\right)$	[62]
285	$C_2H_3 + H_2O \rightarrow C_2H_4 + OH$	$8 \times 10^{-22} \cdot T_g^{2.9} \cdot \exp\left(\frac{-7.48\times10^3}{T_g}\right)$	[17]
286	$C_2H_3 + H_2O_2 \rightarrow C_2H_4 + HO_2$	$2 \times 10^{-14} \cdot \exp\left(\frac{3\times10^2}{T_g}\right)$	[17]
287	$C_2H_2 + OH \rightarrow CH_2CO + H$	$0.5 \cdot 1.3 \times 10^{-10} \cdot \exp\left(\frac{-6.8\times10^3}{T_g}\right)$	[8]
288	$C_2H_2 + OH \rightarrow C_2H + H_2O$	$0.5 \cdot 1.3 \times 10^{-10} \cdot \exp\left(\frac{-6.8\times10^3}{T_g}\right)$	[8]
289	$C_2H_2 + HO_2 \rightarrow C_2H_3 + O_2$	$5.18 \times 10^{-18} \cdot T_g^{1.61} \cdot \exp\left(\frac{-7.1309\times10^3}{T_g}\right)$	[63]
290	$C_2H + OH \rightarrow C_2H_2 + O$	3×10^{-11}	[17]
291	$C_2H + OH \rightarrow CH_2 + CO$	3×10^{-11}	[17]
292	$C_2H + H_2O \rightarrow C_2H_2 + OH$	$2.2 \times 10^{-21} \cdot T_g^{3.05} \cdot \exp\left(\frac{-3.76\times10^2}{T_g}\right)$	[64]
293	$C_2H + HO_2 \rightarrow C_2H_2 + O_2$	3×10^{-11}	[17]
294	$C_2H + HO_2 \rightarrow HCCO + OH$	3×10^{-11}	[17]
295	$C_2H_6 + O \rightarrow C_2H_5 + OH$	$3 \times 10^{-19} \cdot T_g^{2.8} \cdot \exp\left(\frac{-2.92\times10^3}{T_g}\right)$	[8]
296	$C_2H_6 + O_2 \rightarrow C_2H_5 + HO_2$	$1.21 \times 10^{-18} \cdot T_g^{2.5} \cdot \exp\left(\frac{-2.474\times10^4}{T_g}\right)$	[8]
297	$C_2H_5 + O \rightarrow CH_3CHO + H$	8.8×10^{-11}	[8]
298	$C_2H_5 + O \rightarrow CH_3 + HCHO$	6.6×10^{-11}	[8]
299	$C_2H_5 + O \rightarrow C_2H_4 + OH$	4.4×10^{-11}	[8]
300	$C_2H_5 + O_2 \rightarrow C_2H_4 + HO_2$	1×10^{-13}	[8]
301	$C_2H_4 + O \rightarrow CH_3 + HCO$	$0.6 \cdot 2.25 \times 10^{-17} \cdot T_g^{1.88} \cdot \exp\left(\frac{-9.2\times10^1}{T_g}\right)$	[8]

#	Reaction	Rate equation	Ref.
302	$C_2H_4 + O \rightarrow CH_2CO + H_2$	$0.05 \cdot 2.25 \times 10^{-17} \cdot T_g^{1.88} \cdot \exp\left(\frac{-9.2\times10^{1}}{T_g}\right)$	[8]
303	$C_2H_4 + O_2 \rightarrow C_2H_3 + HO_2$	$7 \times 10^{-11} \cdot \exp\left(\frac{-2.9\times10^{4}}{T_g}\right)$	[17]
304	$C_2H_3 + O \rightarrow C_2H_2 + OH$	$1.6666667 \times 10^{-11}$	[8]
305	$C_2H_3 + O \rightarrow CH_3 + CO$	$1.6666667 \times 10^{-11}$	[8]
306	$C_2H_3 + O \rightarrow CH_2 + HCO$	$1.6666667 \times 10^{-11}$	[8]
307	$C_2H_3 + O_2 \rightarrow C_2H_2 + HO_2$	$\frac{6.6\times10^{21}}{N_A} \cdot T_g^{-3.3} \cdot \exp\left(\frac{-5.41\times10^{3}\cdot4.184}{R\cdot T_g}\right)$	[65]
308	$C_2H_3 + O_2 \rightarrow HCHO + HCO$	$\frac{4\times10^{21}}{N_A} \cdot T_g^{-3} \cdot \exp\left(\frac{-2.4\times10^{3}\cdot4.184}{R\cdot T_g}\right)$	[65]
309	$C_2H_2 + O \rightarrow CH_2 + CO$	$0.2 \cdot 1.95 \times 10^{-15} \cdot T_g^{1.4} \cdot \exp\left(\frac{-1.11\times10^{3}}{T_g}\right)$	[8]
310	$C_2H_2 + O \rightarrow H + HCCO$	$0.8 \cdot 1.95 \times 10^{-15} \cdot T_g^{1.4} \cdot \exp\left(\frac{-1.11\times10^{3}}{T_g}\right)$	[8]
311	$C_2H_2 + O_2 \rightarrow HCO + HCO$	$\frac{6.1\times10^{12}}{N_A} \cdot \exp\left(\frac{-5.325\times10^{4}\cdot4.184}{R\cdot T_g}\right)$	[66]
312	$C_2H + O \rightarrow CH + CO$	9.9×10^{-11}	[8]
313	$C_2H + O_2 \rightarrow CO + HCO$	$0.45 \cdot 2.7 \times 10^{-10} \cdot T_g^{-0.35}$	[8]
314	$C_2H + O_2 \rightarrow CH + CO_2$	$0.1 \cdot 2.7 \times 10^{-10} \cdot T_g^{-0.35}$	[8]
315	$C_2H_4 + CO \rightarrow C_2H_3 + HCO$	$2.5 \times 10^{-10} \cdot \exp\left(\frac{-4.56\times10^{4}}{T_g}\right)$	[17]
316	$C_2H_2 + CO \rightarrow C_2H + HCO$	$8 \times 10^{-10} \cdot \exp\left(\frac{-5.37\times10^{4}}{T_g}\right)$	[17]
317	$C_2H_6 + HCO \rightarrow C_2H_5 + HCHO$	$7.8 \times 10^{-20} \cdot T_g^{2.72} \cdot \exp\left(\frac{-9.176\times10^{3}}{T_g}\right)$	[17]
318	$C_2H_6 + CH_3O \rightarrow C_2H_5 + CH_3OH$	$4 \times 10^{-13} \cdot \exp\left(\frac{-3.57\times10^{3}}{T_g}\right)$	[17]
319	$C_2H_6 + CH_2OH \rightarrow C_2H_5 + CH_3OH$	$3.3 \times 10^{-22} \cdot T_g^{2.95} \cdot \exp\left(\frac{-7.033\times10^{3}}{T_g}\right)$	[51]
320	$C_2H_6 + CH_3OO \rightarrow C_2H_5 + CH_3OOH$	$4.9 \times 10^{-13} \cdot \exp\left(\frac{-7.52\times10^{3}}{T_g}\right)$	[17]
321	$C_2H_5 + HCO \rightarrow C_2H_6 + CO$	2×10^{-10}	[17]
322	$C_2H_5 + HCHO \rightarrow C_2H_6 + HCO$	$9.2 \times 10^{-21} \cdot T_g^{2.81} \cdot \exp\left(\frac{-2.95\times10^{3}}{T_g}\right)$	[17]
323	$C_2H_5 + CH_3O \rightarrow C_2H_6 + HCHO$	4×10^{-11}	[17]
324	$C_2H_5 + CH_2OH \rightarrow C_2H_4 + CH_3OH$	4×10^{-12}	[51]
325	$C_2H_5 + CH_2OH \rightarrow C_2H_6 + HCHO$	4×10^{-12}	[51]
326	$C_2H_5 + CH_3OH \rightarrow C_2H_6 + CH_2OH$	$5.3 \times 10^{-23} \cdot T_g^{3.2} \cdot \exp\left(\frac{-4.61\times10^{3}}{T_g}\right)$	[51]
327	$C_2H_5 + CH_3OH \rightarrow C_2H_6 + CH_3O$	$2.4 \times 10^{-23} \cdot T_g^{3.1} \cdot \exp\left(\frac{-4.5\times10^{3}}{T_g}\right)$	[51]
328	$C_2H_5 + CH_3OO \rightarrow CH_3CH_2O + CH_3O$	4×10^{-11}	[17]
329	$C_2H_4 + COOH \rightarrow C_2H_5 + CO_2$	1×10^{-14}	[44]
330	$C_2H_4 + CH_2OH \rightarrow C_2H_5 + HCHO$	$\frac{8\times10^{-14}\cdot\exp\left(\frac{-3.5\times10^{3}}{T_g}\right)\cdot\exp\left(\frac{-2\times10^{3}}{T_g}\right)}{1.0+\exp\left(\frac{-2\times10^{3}}{T_g}\right)}$	[51]
331	$C_2H_3 + HCO \rightarrow C_2H_4 + CO$	1.5×10^{-10}	[17]
332	$C_2H_3 + HCHO \rightarrow C_2H_4 + HCO$	$9 \times 10^{-21} \cdot T_g^{2.81} \cdot \exp\left(\frac{-2.95\times10^{3}}{T_g}\right)$	[17]
333	$C_2H_3 + CH_3O \rightarrow C_2H_4 + HCHO$	4×10^{-11}	[17]
334	$C_2H_3 + CH_2OH \rightarrow C_2H_4 + HCHO$	5×10^{-11}	[51]
335	$C_2H_3 + CH_3OH \rightarrow C_2H_4 + CH_2OH$	$5.3 \times 10^{-23} \cdot T_g^{3.2} \cdot \exp\left(\frac{-3.609\times10^{3}}{T_g}\right)$	[51]
336	$C_2H_3 + CH_3OH \rightarrow C_2H_4 + CH_3O$	$2.4 \times 10^{-23} \cdot T_g^{3.1} \cdot \exp\left(\frac{-3.49\times10^{3}}{T_g}\right)$	[51]
337	$C_2H_2 + COOH \rightarrow C_2H_3 + CO_2$	3×10^{-14}	[44]
338	$C_2H_2 + CH_2OH \rightarrow C_2H_3 + HCHO$	$1.2 \times 10^{-12} \cdot \exp\left(\frac{-4.531\times10^{3}}{T_g}\right)$	[51]
339	$C_2H + HCO \rightarrow C_2H_2 + CO$	1×10^{-10}	[17]

#	Reaction	Rate equation	Ref.
340	$C_2H + CH_3O \rightarrow C_2H_2 + HCHO$	4×10^{-11}	[17]
341	$C_2H + CH_2OH \rightarrow C_2H_2 + HCHO$	6×10^{-11}	[51]
342	$C_2H + CH_3OH \rightarrow C_2H_2 + CH_2OH$	1×10^{-11}	[51]
343	$C_2H + CH_3OH \rightarrow C_2H_2 + CH_3O$	2×10^{-12}	[51]
344	$C_2H + CH_3OO \rightarrow CH_3O + HCCO$	4×10^{-11}	[17]
345	$H + HCCO \rightarrow CH_2 + CO$	$9.92 \times 10^{-13} \cdot T_g^{0.76} \cdot \exp\left(\frac{4.38\times10^2}{T_g}\right)$	[67]
346	$CH_2CO + H \rightarrow CH_3 + CO$	$\frac{1.11\times10^7}{N_A} \cdot T_g^2 \cdot \exp\left(\frac{-2\times10^3\cdot4.184}{R\cdot T_g}\right)$	[68]
347	$CH_2CO + H \rightarrow H_2 + HCCO$	$\frac{1.8\times10^{14}}{N_A} \cdot \exp\left(\frac{-8.6\times10^3\cdot4.184}{R\cdot T_g}\right)$	[68]
348	$CH_2CO + H \rightarrow CH_3CO$	$\frac{1.63\times10^9}{N_A} \cdot T_g^{1.3766} \cdot \exp\left(\frac{-1.664\times10^3\cdot4.184}{R\cdot T_g}\right)$	[69]
349	$CH_3CO + H \rightarrow CH_3 + HCO$	$\frac{0.65\cdot2\times10^{13}}{N_A}$	[70, 71]
350	$CH_3CO + H \rightarrow CH_2CO + H_2$	$\frac{0.35\cdot2\times10^{13}}{N_A}$	[70, 71]
351	$CH_3CO + H \rightarrow CH_3CHO$	$6.02 \times 10^{-11} \cdot T_g^{0.16}$	[62]
352	$CH_3CO + H_2 \rightarrow CH_3CHO + H$	$6.8 \times 10^{-18} \cdot T_g^{1.82} \cdot \exp\left(\frac{-8.862\times10^3}{T_g}\right)$	[17]
353	$CH_3CHO + H \rightarrow CH_3CO + H_2$	$2.18 \times 10^{-19} \cdot T_g^{2.58} \cdot \exp\left(\frac{-6.14\times10^2}{T_g}\right)$	[72]
354	$CH_3CHO + H \rightarrow CH_3CH_2O$	$7.66 \times 10^{-17} \cdot T_g^{1.71} \cdot \exp\left(\frac{-3.57\times10^3}{T_g}\right)$	[72]
355	$CH_3CHO + H \rightarrow CH_3CHOH$	$2.89 \times 10^{-18} \cdot T_g^{2.2} \cdot \exp\left(\frac{-3.78\times10^3}{T_g}\right)$	[72]
356	$CH_3CH_2O + H \rightarrow CH_2OH + CH_3$	$2.26 \times 10^{-12} \cdot T_g^{0.701} \cdot \exp\left(\frac{-1.74\times10^2}{T_g}\right)$	[73]
357	$CH_3CH_2O + H \rightarrow CH_3CH_2OH$	$5.11 \times 10^{-13} \cdot T_g^{0.894} \cdot \exp\left(\frac{-6.5}{T_g}\right)$	[73]
358	$CH_3CH_2O + H \rightarrow C_2H_5 + OH$	$9.04 \times 10^{-16} \cdot T_g^{1.27} \cdot \exp\left(\frac{-1.57\times10^2}{T_g}\right)$	[73]
359	$CH_3CH_2O + H \rightarrow CH_3CHOH + H$	$1.33 \times 10^{-22} \cdot T_g^{3.1} \cdot \exp\left(\frac{-1.42\times10^2}{T_g}\right)$	[73]
360	$CH_3CH_2O + H \rightarrow C_2H_4 + H_2O$	$9.95 \times 10^{-10} \cdot T_g^{-0.813} \cdot \exp\left(\frac{-3.59\times10^2}{T_g}\right)$	[73]
361	$CH_3CH_2O + H \rightarrow CH_3CHO + H_2$	$1.25 \times 10^{-20} \cdot T_g^{1.78} \cdot \exp\left(\frac{-4.07\times10^1}{T_g}\right)$ $+1.24 \times 10^{-14} \cdot T_g^{1.15} \cdot \exp\left(\frac{-3.39\times10^2}{T_g}\right)$	[73]
362	$CH_3CH_2O + H \rightarrow CH_4 + HCHO$	$1.32 \times 10^{-21} \cdot T_g^{2.21} \cdot \exp\left(\frac{9.05\times10^1}{T_g}\right)$	[73]
363	$CH_3CHOH + H \rightarrow CH_3CH_2OH$	$5.99 \times 10^{-11} \cdot T_g^{0.06} \cdot \exp\left(\frac{-2.2\times10^2}{T_g}\right)$	[73]
364	$CH_3CHOH + H \rightarrow CH_2OH + CH_3$	$1.44 \times 10^{-7} \cdot T_g^{-0.891} \cdot \exp\left(\frac{-1.461\times10^3}{T_g}\right)$	[73]
365	$CH_3CHOH + H \rightarrow C_2H_5 + OH$	$4.02 \times 10^{-9} \cdot T_g^{-0.83} \cdot \exp\left(\frac{-2.414\times10^3}{T_g}\right)$	[73]
366	$CH_3CHOH + H \rightarrow CH_3CH_2O + H$	$4.95 \times 10^{-23} \cdot T_g^{2.94} \cdot \exp\left(\frac{-4.266\times10^3}{T_g}\right)$	[73]
367	$CH_3CHOH + H \rightarrow C_2H_4 + H_2O$	$7.81 \times 10^{-3} \cdot T_g^{-3.02} \cdot \exp\left(\frac{-1.432\times10^3}{T_g}\right)$	[73]
368	$CH_3CHOH + H \rightarrow CH_3CHO + H_2$	$7.42 \times 10^{-21} \cdot T_g^{1.62} \cdot \exp\left(\frac{5.4}{T_g}\right)$ $+2.26 \times 10^{-15} \cdot T_g^{1.29} \cdot \exp\left(\frac{-1.421\times10^3}{T_g}\right)$	[73]
369	$CH_3CHOH + H \rightarrow CH_4 + HCHO$	$5.56 \times 10^{-22} \cdot T_g^{2.1} \cdot \exp\left(\frac{-1.07\times10^2}{T_g}\right)$	[73]
370	$CH_3CH_2OH + H \rightarrow C_2H_5 + H_2O$	$\frac{5.9\times10^{11}}{N_A} \cdot \exp\left(\frac{-3.45\times10^3\cdot4.184}{R\cdot T_g}\right)$	[74]
371	$CH_3CH_2OH + H \rightarrow CH_3CHOH + H_2$	$1.46 \times 10^{-19} \cdot T_g^{2.68} \cdot \exp\left(\frac{-1.467\times10^3}{T_g}\right)$	[75]
372	$CH_3CH_2OH + H \rightarrow CH_2CH_2OH + H_2$	$8.82 \times 10^{-20} \cdot T_g^{2.81} \cdot \exp\left(\frac{-3.772\times10^3}{T_g}\right)$	[75]
373	$CH_3CH_2OH + H \rightarrow CH_3CH_2O + H_2$	$1.57 \times 10^{-21} \cdot T_g^{3.14} \cdot \exp\left(\frac{-4.379\times10^3}{T_g}\right)$	[75]
374	$HCCO + OH \rightarrow CH_2CO + O$	$2.1 \times 10^{-18} \cdot T_g^{1.99} \cdot \exp\left(\frac{-1.128\times10^4\cdot4.184}{R\cdot T_g}\right)$	[76]
375	$CH_2CO + OH \rightarrow CH_2OH + CO$	$0.6 \cdot 2.8 \times 10^{-12} \cdot \exp\left(\frac{5.1\times10^2}{T_g}\right)$	[8]

#	Reaction	Rate equation	Ref.
376	$CH_2CO + OH \rightarrow H_2O + HCCO$	$0.01 \cdot 2.8 \times 10^{-12} \cdot \exp\left(\frac{5.1\times10^2}{T_g}\right)$	[8]
377	$CH_2CO + OH \rightarrow HCHO + HCO$	$0.02 \cdot 2.8 \times 10^{-12} \cdot \exp\left(\frac{5.1\times10^2}{T_g}\right)$	[8]
378	$CH_2CO + OH \rightarrow CH_3 + CO_2$	$0.37 \cdot 2.8 \times 10^{-12} \cdot \exp\left(\frac{5.1\times10^2}{T_g}\right)$	[8]
379	$CH_3CO + OH \rightarrow CH_2CO + H_2O$	2×10^{-11}	[17]
380	$CH_3CO + H_2O_2 \rightarrow CH_3CHO + HO_2$	$3 \times 10^{-13} \cdot \exp\left(\frac{-4.14\times10^3}{T_g}\right)$	[17]
381	$CH_3CHO + OH \rightarrow CH_3CO + H_2O$	$0.93 \cdot 4.8 \times 10^{-16} \cdot T_g^{1.35} \cdot \exp\left(\frac{7.92\times10^2}{T_g}\right)$	[8]
382	$CH_3CHO + OH \rightarrow CH_3 + HCOOH$	$0.03 \cdot 4.8 \times 10^{-16} \cdot T_g^{1.35} \cdot \exp\left(\frac{7.92\times10^2}{T_g}\right)$	[8, 77]
383	$CH_3CHO + OH \rightarrow CH_3COOH + H$	$0.02 \cdot 4.8 \times 10^{-16} \cdot T_g^{1.35} \cdot \exp\left(\frac{7.92\times10^2}{T_g}\right)$	[8, 77]
384	$CH_3CHO + HO_2 \rightarrow CH_3CO + H_2O_2$	$6.8 \times 10^{-20} \cdot T_g^{2.5} \cdot \exp\left(\frac{-5.135\times10^3}{T_g}\right)$	[8]
385	$CH_3CH_2OH + OH \rightarrow CH_2CH_2OH + H_2O$	$\frac{1.74\times10^{11}}{N_A} \cdot T_g^{0.27} \cdot \exp\left(\frac{-6\times10^2\cdot4.184}{R\cdot T_g}\right)$	[78]
386	$CH_3CH_2OH + OH \rightarrow CH_3CHOH + H_2O$	$\frac{4.64\times10^{11}}{N_A} \cdot T_g^{0.15}$	[78]
387	$CH_3CH_2OH + OH \rightarrow CH_3CH_2O + H_2O$	$\frac{7.46\times10^{11}}{N_A} \cdot T_g^{0.3} \cdot \exp\left(\frac{-1.634\times10^3\cdot4.184}{R\cdot T_g}\right)$	[78]
388	$CH_3CH_2OH + HO_2 \rightarrow CH_3CHOH + H_2O_2$	$\frac{5.544\times10^{18}}{N_A} \cdot T_g^{-1.808} \cdot \exp\left(\frac{-8.29197\times10^3}{T_g}\right)$	[79]
389	$HCCO + O \rightarrow CH + CO_2$	$4.9 \times 10^{-11} \cdot \exp\left(\frac{-5.6\times10^2}{T_g}\right)$	[8]
390	$CH_2CO + O \rightarrow HCCO + OH$	$3.11 \times 10^{-10} \cdot \exp\left(\frac{-1.669\times10^4\cdot4.184}{R\cdot T_g}\right)$	[76]
391	$CH_2CO + O \rightarrow CO + HCHO$	$0.2 \cdot 3 \times 10^{-12} \cdot \exp\left(\frac{-6.8\times10^2}{T_g}\right)$	[8]
392	$CH_2CO + O \rightarrow HCO + HCO$	$0.1 \cdot 3 \times 10^{-12} \cdot \exp\left(\frac{-6.8\times10^2}{T_g}\right)$	[8]
393	$CH_2CO + O \rightarrow CH_2 + CO_2$	$0.6 \cdot 3 \times 10^{-12} \cdot \exp\left(\frac{-6.8\times10^2}{T_g}\right)$	[8]
394	$CH_3CO + O \rightarrow CH_2CO + OH$	8.75×10^{-11}	[8]
395	$CH_3CO + O \rightarrow CH_3 + CO_2$	2.625×10^{-10}	[8]
396	$CH_3CHO + O \rightarrow CH_3CO + OH$	$\frac{5\times10^{12}}{N_A} \cdot \exp\left(\frac{-7.5\times10^3}{R\cdot T_g}\right)$	[71]
397	$CH_3CHO + O_2 \rightarrow CH_3CO + HO_2$	$2 \times 10^{-19} \cdot T_g^{2.5} \cdot \exp\left(\frac{-1.89\times10^4}{T_g}\right)$	[8]
398	$CH_3CH_2O + O_2 \rightarrow CH_3CHO + HO_2$	$3.8 \times 10^{-14} \cdot \exp\left(\frac{-4.4\times10^2}{T_g}\right)$	[8]
399	$CH_3CHOH + O \rightarrow CH_3 + HCOOH$	$3.9 \times 10^{-10} \cdot \left(\frac{T_g}{3\times10^2}\right)^{0.18} \cdot \exp\left(\frac{-0.49}{T_g}\right)$	[80]
400	$CH_3CHOH + O \rightarrow CH_3CHO + OH$	$4.8 \times 10^{-11} \cdot \left(\frac{T_g}{3\times10^2}\right)^{0.19} \cdot \exp\left(\frac{-0.39}{T_g}\right)$	[80]
401	$CH_3CHOH + O \rightarrow CH_3COOH + H$	$2.2 \times 10^{-10} \cdot \left(\frac{T_g}{3\times10^2}\right)^{0.16} \cdot \exp\left(\frac{-0.59}{T_g}\right)$	[80]
402	$CH_3CHOH + O_2 \rightarrow CH_3CHO + HO_2$	$\frac{5.28\times10^{17}}{N_A} \cdot T_g^{-1.638} \cdot \exp\left(\frac{-0.839\cdot4.184\times10^3}{R\cdot T_g}\right)$	[81]
403	$CH_2CH_2OH + O \rightarrow CH_2OH + HCHO$	$4.6 \times 10^{-10} \cdot \left(\frac{T_g}{3\times10^2}\right)^{0.17} \cdot \exp\left(\frac{-0.51}{T_g}\right)$	[80]
404	$CH_3CH_2OH + O \rightarrow CH_3CHOH + OH$	$0.99 \cdot 1 \times 10^{-18} \cdot T_g^{2.5} \cdot \exp\left(\frac{-9.3\times10^2}{T_g}\right)$	[8]
405	$CH_3CH_2OH + O \rightarrow CH_2CH_2OH + OH$	$0.005 \cdot 1 \times 10^{-18} \cdot T_g^{2.5} \cdot \exp\left(\frac{-9.3\times10^2}{T_g}\right)$	[8]
406	$CH_3CH_2OH + O \rightarrow CH_3CH_2O + OH$	$0.005 \cdot 1 \times 10^{-18} \cdot T_g^{2.5} \cdot \exp\left(\frac{-9.3\times10^2}{T_g}\right)$	[8]
407	$CH_3CH_2OH + O_2 \rightarrow CH_3CHOH + HO_2$	$4 \times 10^{-19} \cdot T_g^{2.5} \cdot \exp\left(\frac{-2.217\times10^4}{T_g}\right)$	[8]
408	$CH_3CH_2OH + O_2 \rightarrow CH_2CH_2OH + HO_2$	$6 \times 10^{-19} \cdot T_g^{2.5} \cdot \exp\left(\frac{-2.403\times10^4}{T_g}\right)$	[8]
409	$CH_3CH_2OH + O_2 \rightarrow CH_3CH_2O + HO_2$	$2 \times 10^{-19} \cdot T_g^{2.5} \cdot \exp\left(\frac{-2.653\times10^4}{T_g}\right)$	[8]
410	$CH_2CO + CH_3 \rightarrow C_2H_5 + CO$	$\frac{1.24\times10^5}{N_A} \cdot T_g^{2.29} \cdot \exp\left(\frac{-1.0642\times10^4\cdot4.184}{R\cdot T_g}\right)$	[82]
411	$CH_2CO + CH_3 \rightarrow CH_4 + HCCO$	$\frac{1.55\times10^2}{N_A} \cdot T_g^{3.38} \cdot \exp\left(\frac{-1.0512\times10^4\cdot4.184}{R\cdot T_g}\right)$	[82]
412	$CH_2 + CH_2CO \rightarrow C_2H_4 + CO$	$\frac{1\times10^{12}}{N_A}$	[83]
413	$CH_2 + CH_2CO \rightarrow CH_3 + HCCO$	$\frac{3.6\times10^{13}}{N_A} \cdot \exp\left(\frac{-1.1\times10^4\cdot4.184}{R\cdot T_g}\right)$	[68]

#	Reaction	Rate equation	Ref.
414	$CH_3CO + CH_4 \rightarrow CH_3 + CH_3CHO$	$3.6 \times 10^{-21} \cdot T_g^{2.88} \cdot \exp\left(\frac{-1.08\times10^4}{T_g}\right)$	[17]
415	$CH_3 + CH_3CO \rightarrow CH_2CO + CH_4$	$\frac{6.1\times10^9\cdot1\times10^3}{N_A}$	[84]
416	$CH_2 + CH_3CO \rightarrow CH_2CO + CH_3$	3×10^{-11}	[17]
417	$CH_3 + CH_3CHO \rightarrow CH_3CO + CH_4$	$0.993 \cdot 5.8 \times 10^{-32} \cdot T_g^{6.21} \cdot \exp\left(\frac{-8.2\times10^2}{T_g}\right)$	[8]
418	$CH_3 + CH_3CH_2OH \rightarrow CH_3CHOH + CH_4$	$\frac{2.476\times10^1}{N_A} \cdot T_g^{3.368} \cdot \exp\left(\frac{-3.95579\times10^3}{T_g}\right)$	[79]
419	$CH_3 + CH_3CH_2OH \rightarrow CH_2CH_2OH + CH_4$	$\frac{1.861\times10^2}{N_A} \cdot T_g^{3.45} \cdot \exp\left(\frac{-5.54285\times10^3}{T_g}\right)$	[79]
420	$CH_3 + CH_3CH_2OH \rightarrow CH_3CH_2O + CH_4$	$\frac{0.09533}{N_A} \cdot T_g^{4.159} \cdot \exp\left(\frac{-4.119\times10^3}{T_g}\right)$	[79]
421	$C_2H_6 + CH_3CO \rightarrow C_2H_5 + CH_3CHO$	$3 \times 10^{-20} \cdot T_g^{2.75} \cdot \exp\left(\frac{-8.82\times10^3}{T_g}\right)$	[17]
422	$C_2H_5 + CH_3CHO \rightarrow C_2H_6 + CH_3CO$	$\frac{1.25892541\times10^{12}}{N_A} \cdot \exp\left(\frac{-8.5\cdot4.184\times10^3}{R\cdot T_g}\right)$	[85]
423	$CH_3CO + HCO \rightarrow CH_3CHO + CO$	1.5×10^{-11}	[17]
424	$CH_3CO + HCHO \rightarrow CH_3CHO + HCO$	$3 \times 10^{-13} \cdot \exp\left(\frac{-6.5\times10^3}{T_g}\right)$	[17]
425	$CH_3CO + CH_3O \rightarrow CH_2CO + CH_3OH$	1×10^{-11}	[17]
426	$CH_3CO + CH_3O \rightarrow CH_3CHO + HCHO$	1×10^{-11}	[17]
427	$CH_3CO + CH_3OH \rightarrow CH_2OH + CH_3CHO$	$8.06 \times 10^{-21} \cdot T_g^{2.99} \cdot \exp\left(\frac{-6.21\times10^3}{T_g}\right)$	[51]
428	$CH_3CHO + CH_3O \rightarrow CH_3CO + CH_3OH$	$\frac{1.69\times10^5}{N_A} \cdot T_g^{2.04} \cdot \exp\left(\frac{-2.353\times10^3\cdot4.184}{R\cdot T_g}\right)$ $+\frac{9.62\times10^3}{N_A} \cdot T_g^{2.5} \cdot \exp\left(\frac{-1.59\times10^2\cdot4.184}{R\cdot T_g}\right)$	[86]
429	$CH_3CHO + CH_3OO \rightarrow CH_3CO + CH_3OOH$	$\frac{0.322}{N_A} \cdot T_g^{3.94} \cdot \exp\left(\frac{-9.503\times10^3\cdot4.184}{R\cdot T_g}\right)$ $+\frac{4.99\times10^{-6}}{N_A} \cdot T_g^{4.98} \cdot \exp\left(\frac{-5.2682\times10^3\cdot4.184}{R\cdot T_g}\right)$	[86]
430	$CH_3CO + CH_3CO \rightarrow CH_2CO + CH_3CHO$	$\frac{9\times10^9\cdot1\times10^3}{N_A}$	[84]
431	$COOH \rightarrow CO + OH$	$k_0 = \frac{10^{2.5137\times10^1}}{N_A} \cdot T_g^{-2.396} \cdot \exp\left(\frac{-1.8862\times10^4}{T_g}\right)$ $k_\infty = 10^{1.4074\times10^1} \cdot T_g^{0.132} \cdot \exp\left(\frac{-1.8349\times10^4}{T_g}\right)$ $F_c = 0.729 \cdot \exp\left(\frac{-5.13\times10^2}{T_g}\right)$ $+\exp\left(\frac{-T_g}{5.4\times10^2}\right)$	[87][a]
432	$COOH \rightarrow CO_2 + H$	$k_0 = \frac{10^{2.6775\times10^1}}{N_A} \cdot T_g^{-3.148} \cdot \exp\left(\frac{-1.8629\times10^4}{T_g}\right)$ $k_\infty = 10^{1.1915\times10^1} \cdot T_g^{0.413} \cdot \exp\left(\frac{-1.7783\times10^4}{T_g}\right)$ $F_c = 1.049 \cdot \exp\left(\frac{-2.407\times10^3}{T_g}\right)$ $+\exp\left(\frac{-T_g}{8.23\times10^2}\right)$	[87][a]
433	$HCHO \rightarrow H + HCO$	$8.09 \times 10^{-9} \cdot \exp\left(\frac{-3.805\times10^4}{T_g}\right) \cdot n_M$	[8]
434	$CH_2OH \rightarrow H + HCHO$	$k_0 = \frac{6.01\times10^{33}}{N_A} \cdot T_g^{-5.39} \cdot \exp\left(\frac{-3.62\times10^4\cdot4.184}{R\cdot T_g}\right)$ $k_\infty = 2.8 \times 10^{14} \cdot T_g^{-0.73} \cdot \exp\left(\frac{-3.282\times10^4\cdot4.184}{R\cdot T_g}\right)$ $F_c = (1-0.96) \cdot \exp\left(\frac{-T_g}{6.76\times10^1}\right)$ $+0.96 \cdot \exp\left(\frac{-T_g}{1.855\times10^3}\right)$ $+\exp\left(\frac{-7.543\times10^3}{T_g}\right)$	[88][a]
435	$CH_3OH \rightarrow CH_3 + OH$	$0.8 \cdot k$ $k_0 = 1.1 \times 10^{-7} \cdot \exp\left(\frac{-3.308\times10^4}{T_g}\right)$ $k_\infty = 2.5 \times 10^{19} \cdot T_g^{-0.94} \cdot \exp\left(\frac{-4.703\times10^4}{T_g}\right)$ $F_c = 0.18 \cdot \exp\left(\frac{-T_g}{2\times10^2}\right)$ $+0.82 \cdot \exp\left(\frac{-T_g}{1.438\times10^3}\right)$	[8, 89][a]

#	Reaction	Rate equation	Ref.
436	$CH_3OH \rightarrow CH_2 + H_2O$	$0.15 \cdot k$<br>$k_0 = 1.1 \times 10^{-7} \cdot \exp\left(\frac{-3.308\times10^4}{T_g}\right)$<br>$k_\infty = 2.5 \times 10^{19} \cdot T_g^{-0.94} \cdot \exp\left(\frac{-4.703\times10^4}{T_g}\right)$<br>$F_c = 0.18 \cdot \exp\left(\frac{-T_g}{2\times10^2}\right)$<br>$+0.82 \cdot \exp\left(\frac{-T_g}{1.438\times10^3}\right)$	[8, 89][a]
437	$CH_3OH \rightarrow CH_2OH + H$	$0.05 \cdot k$<br>$k_0 = 1.1 \times 10^{-7} \cdot \exp\left(\frac{-3.308\times10^4}{T_g}\right)$<br>$k_\infty = 2.5 \times 10^{19} \cdot T_g^{-0.94} \cdot \exp\left(\frac{-4.703\times10^4}{T_g}\right)$<br>$F_c = 0.18 \cdot \exp\left(\frac{-T_g}{2\times10^2}\right)$<br>$+0.82 \cdot \exp\left(\frac{-T_g}{1.438\times10^3}\right)$	[8, 89][a]
438	$CH_3OOH \rightarrow CH_3O + OH$	$6 \times 10^{14} \cdot \exp\left(\frac{-2.13\times10^4}{T_g}\right)$	[8]
439	$HCCO \rightarrow CH + CO$	$\frac{6\times10^{15}}{N_A} \cdot \exp\left(\frac{-2.96\times10^4}{T_g}\right) \cdot n_M$	[90]
440	$CH_2CO \rightarrow CH_2 + CO$	$\frac{2.3\times10^{15}}{N_A} \cdot \exp\left(\frac{-2.899\times10^4}{T_g}\right) \cdot n_M$	[83]
441	$CH_3CO \rightarrow CH_3 + CO$	$k_0 = 1 \times 10^{-8} \cdot \exp\left(\frac{-7.08\times10^3}{T_g}\right)$<br>$k_\infty = 2 \times 10^{13} \cdot \exp\left(\frac{-8.63\times10^3}{T_g}\right)$<br>$F_c = 0.5$	[8][a]
442	$CH_3CO \rightarrow CH_2CO + H$	$1.36 \times 10^8 \cdot T_g^{1.9433} \cdot \exp\left(\frac{-4.6005\times10^4\cdot4.184}{R\cdot T_g}\right)$	[69]
443	$CH_3CHO \rightarrow CH_3CO + H$	$5 \times 10^{14} \cdot \exp\left(\frac{-8.79\times10^4\cdot4.184}{R\cdot T_g}\right)$	[91]
444	$CH_3CHO \rightarrow CH_3 + HCO$	$2.1 \times 10^{16} \cdot \exp\left(\frac{-4.1135\times10^4}{T_g}\right)$	[8]
445	$CH_3COOH \rightarrow CH_3 + COOH$	$10^{5.7\times10^1} \cdot T_g^{-1.204\times10^1} \cdot \exp\left(\frac{-1.1313\times10^5\cdot4.182}{R\cdot T_g}\right)$	[92]
446	$CH_3CH_2O \rightarrow CH_3CHO + H$	$\frac{5.43\times10^{15}}{N_A} \cdot T_g^{-0.69} \cdot \exp\left(\frac{-2.223\times10^4\cdot4.184}{R\cdot T_g}\right)$	[48]
447	$CH_3CH_2O \rightarrow CH_3 + HCHO$	$k_0 = \frac{4.7\times10^{25}}{N_A} \cdot T_g^{-3} \cdot \exp\left(\frac{-8.32\times10^3}{T_g}\right)$<br>$k_\infty = 6.31 \times 10^{10} \cdot T_g^{0.93} \cdot \exp\left(\frac{-8.605\times10^3}{T_g}\right)$<br>$F_c = (1-0.426) \cdot \exp\left(\frac{-T_g}{0.3}\right)$<br>$+0.426 \cdot \exp\left(\frac{-T_g}{2.278\times10^3}\right)$<br>$+\exp\left(\frac{-1\times10^5}{T_g}\right)$	[93][a]
448	$CH_3CHOH \rightarrow CH_3CHO + H$	$k_0 = \frac{1.77\times10^{16}}{N_A} \cdot \exp\left(\frac{-1.0458\times10^4}{T_g}\right)$<br>$k_\infty = 6.17 \times 10^9 \cdot T_g^{1.31} \cdot \exp\left(\frac{-1.6998\times10^4}{T_g}\right)$<br>$F_c = (1-0.187) \cdot \exp\left(\frac{-T_g}{6.52\times10^1}\right)$<br>$+0.187 \cdot \exp\left(\frac{-T_g}{2.568\times10^3}\right)$<br>$+\exp\left(\frac{-4.1226\times10^4}{T_g}\right)$	[93][a]
449	$CH_3CHOH \rightarrow CH_3 + HCHO$	$k_0 = \frac{5.86\times10^{15}}{N_A} \cdot \exp\left(\frac{-1.0735\times10^4}{T_g}\right)$<br>$k_\infty = 2.22 \times 10^9 \cdot T_g^{1.18} \cdot \exp\left(\frac{-1.7103\times10^4}{T_g}\right)$<br>$F_c = (1-0.124) \cdot \exp\left(\frac{-T_g}{1}\right)$<br>$+0.124 \cdot \exp\left(\frac{-T_g}{1.729\times10^3}\right)$<br>$+\exp\left(\frac{-5\times10^4}{T_g}\right)$	[93][a]
450	$CH_3CH_2OH \rightarrow CH_2OH + CH_3$	$k_0 = \frac{2.88\times10^{85}}{N_A} \cdot T_g^{-1.89\times10^1} \cdot \exp\left(\frac{-5.5317\times10^4}{T_g}\right)$<br>$k_\infty = 5.94 \times 10^{23} \cdot T_g^{-1.68} \cdot \exp\left(\frac{-4.588\times10^4}{T_g}\right)$<br>$F_c = 0.5 \cdot \exp\left(\frac{-T_g}{2\times10^2}\right)$<br>$+0.5 \cdot \exp\left(\frac{-T_g}{8.9\times10^2}\right)$<br>$+\exp\left(\frac{-4.6\times10^3}{T_g}\right)$	[78][a]

#	Reaction	Rate equation	Ref.
451	$M+O+O \rightarrow M+O_2$	$5.2\times10^{-35}\cdot\exp\left(\frac{9\times10^2}{T_g}\right)$	[17]
452	$O+O_3 \rightarrow O_2+O_2$	$8\times10^{-12}\cdot\exp\left(\frac{-2.060\times10^3}{T_g}\right)$	[31]
453	$M+O+O_2 \rightarrow M+O_3$	$5.4\times10^{-34}\cdot\left(\frac{3\times10^2}{T_g}\right)^{1.9}$	[29]
454	$O_2+O_2 \rightarrow O+O_3$	$2\times10^{-11}\cdot\exp\left(\frac{-4.980\times10^4}{T_g}\right)$	[29]
455	$M+O_2 \rightarrow M+O+O$	$3\times10^{-6}\cdot T_g^{-1}\cdot\exp\left(\frac{-5.938\times10^4}{T_g}\right)$	[17]
456	$M+O_3 \rightarrow M+O+O_2$	$6.6\times10^{-10}\cdot\exp\left(\frac{-1.160\times10^4}{T_g}\right)$	[29]
457	$M+C+O \rightarrow M+CO$	$9.1\times10^{-22}\cdot T_g^{-3.08}\cdot\exp\left(\frac{-2.114\times10^3}{T_g}\right)$	[94]
458	$C+O_2 \rightarrow CO+O$	$\frac{1.2\times10^{14}}{N_A}\cdot\exp\left(\frac{-2.01\times10^3}{T_g}\right)$	[95]
459	$CO_2+O \rightarrow CO+O_2$	$\frac{1.7\times10^{13}}{N_A}\cdot\exp\left(\frac{-2.65\times10^4}{T_g}\right)$	[94]
460	$M+CO+O \rightarrow M+CO_2$	$8.3\times10^{-34}\cdot\exp\left(\frac{-1.51\times10^3}{T_g}\right)$	[17]
461	$CO+O_2 \rightarrow CO_2+O$	$4.2\times10^{-12}\cdot\exp\left(\frac{-2.4\times10^4}{T_g}\right)$	[17]
462	$CO+O_3 \rightarrow CO_2+O_2$	4×10^{-25}	[96]
463	$C+CO_2 \rightarrow CO+CO$	1×10^{-15}	[97]
464	$M+CO_2 \rightarrow M+CO+O$	$\frac{3.65\times10^{14}}{N_A}\cdot\exp\left(\frac{-5.2525\times10^4}{T_g}\right)$	[98]
465	$M+CO \rightarrow M+C+O$	$1.46\times10^{6}\cdot T_g^{-3.52}\cdot\exp\left(\frac{-1.287\times10^5}{T_g}\right)$	[94]
466	$M+H+H \rightarrow M+H_2$	$\frac{1.5\times10^{-29}}{N_A}\cdot T_g^{-1.3}$	[17]
467	$M+H_2 \rightarrow M+H+H$	$\frac{7.6\times10^{-5}}{N_A}\cdot T_g^{-1.4}\cdot\exp\left(\frac{-5.253\times10^4}{T_g}\right)$	[17]
468	$C+H_2 \rightarrow CH+H$	$k_{rev}\cdot K_{eq}$	b
469	$C_2H_4 \rightarrow C+CH_4$	$k_{rev}\cdot K_{eq}$	b
470	$C_2H_2+H \rightarrow C+CH_3$	$k_{rev}\cdot K_{eq}$	b
471	$C_2H+H \rightarrow C+CH_2$	$k_{rev}\cdot K_{eq}$	b
472	$C_2H_6+H \rightarrow CH_3+CH_4$	$k_{rev}\cdot K_{eq}$	b
473	$C_2H_5+H_2 \rightarrow CH_3+CH_4$	$k_{rev}\cdot K_{eq}$	b
474	$C_2H_4+H \rightarrow CH+CH_4$	$k_{rev}\cdot K_{eq}$	b
475	$C_2H_4+H \rightarrow CH_2+CH_3$	$k_{rev}\cdot K_{eq}$	b
476	$C_2H_2+H_2 \rightarrow CH_2+CH_2$	$k_{rev}\cdot K_{eq}$	b
477	$C_2H_2 \rightarrow CH+CH$	$k_{rev}\cdot K_{eq}$	b
478	$CH_2+H \rightarrow CH_3$	$k_{rev}\cdot K_{eq}$	b
479	$CH+H \rightarrow CH_2$	$k_{rev}\cdot K_{eq}$	b
480	$C+H_2 \rightarrow CH_2$	$k_{rev}\cdot K_{eq}$	b
481	$C+H \rightarrow CH$	$k_{rev}\cdot K_{eq}$	b
482	$C_2H_2+CH_2 \rightarrow C+C_2H_4$	$k_{rev}\cdot K_{eq}$	b
483	$C_2H_4+CH_3 \rightarrow C_2H_6+CH$	$k_{rev}\cdot K_{eq}$	b
484	$C_2H_4+CH_4 \rightarrow C_2H_5+CH_3$	$k_{rev}\cdot K_{eq}$	b
485	$C_2H_4+CH_3 \rightarrow C_2H_5+CH_2$	$k_{rev}\cdot K_{eq}$	b
486	$C_2H_2+CH_4 \rightarrow C_2H_3+CH_3$	$k_{rev}\cdot K_{eq}$	b
487	$C_2H_2+CH_3 \rightarrow C_2H_3+CH_2$	$k_{rev}\cdot K_{eq}$	b
488	$C_2H_2+CH \rightarrow C_2H+CH_2$	$k_{rev}\cdot K_{eq}$	b
489	$C_2H_4+C_2H_6 \rightarrow C_2H_5+C_2H_5$	$k_{rev}\cdot K_{eq}$	b

#	Reaction	Rate equation	Ref.
490	$C_2H_2 + C_2H_6 \rightarrow C_2H_3 + C_2H_5$	$k_{rev} \cdot K_{eq}$	b
491	$C_2H_2 + C_2H_4 \rightarrow C_2H + C_2H_5$	$k_{rev} \cdot K_{eq}$	b
492	$C_2H_2 + C_2H_3 \rightarrow C_2H + C_2H_4$	$k_{rev} \cdot K_{eq}$	b
493	$H_2 + O_2 \rightarrow OH + OH$	$k_{rev} \cdot K_{eq}$	b
494	$OH + OH \rightarrow H_2O_2$	$k_{rev} \cdot K_{eq}$	b
495	$M + H + OH \rightarrow M + H_2O$	$k_{rev} \cdot K_{eq}$	b
496	$H_2O_2 + O \rightarrow H_2O + O_2$	$k_{rev} \cdot K_{eq}$	b
497	$M + HO_2 \rightarrow M + H + O_2$	$k_{rev} \cdot K_{eq}$	b
498	$HO_2 + O \rightarrow H + O_3$	$k_{rev} \cdot K_{eq}$	b
499	$HO_2 + OH \rightarrow H_2 + O_3$	$k_{rev} \cdot K_{eq}$	b
500	$CO + H \rightarrow C + OH$	$k_{rev} \cdot K_{eq}$	b
501	$HCO \rightarrow CO + H$	$k_{rev} \cdot K_{eq}$	b
502	$CO_2 + OH \rightarrow CO + HO_2$	$k_{rev} \cdot K_{eq}$	b
503	$COOH + OH \rightarrow CO + H_2O_2$	$k_{rev} \cdot K_{eq}$	b
504	$H + HCHO \rightarrow CH_3 + O$	$k_{rev} \cdot K_{eq}$	b
505	$HCHO + OH \rightarrow CH_3 + O_2$	$k_{rev} \cdot K_{eq}$	b
506	$CH_3OO \rightarrow CH_3 + O_2$	$k_{rev} \cdot K_{eq}$	b
507	$CO + H_2 \rightarrow CH_2 + O$	$k_{rev} \cdot K_{eq}$	b
508	$HCHO + O \rightarrow CH_2 + O_2$	$k_{rev} \cdot K_{eq}$	b
509	$CO + H_2O \rightarrow CH_2 + O_2$	$k_{rev} \cdot K_{eq}$	b
510	$CO + H \rightarrow CH + O$	$k_{rev} \cdot K_{eq}$	b
511	$CO_2 + H \rightarrow CH + O_2$	$k_{rev} \cdot K_{eq}$	b
512	$CO + OH \rightarrow CH + O_2$	$k_{rev} \cdot K_{eq}$	b
513	$HCO + O \rightarrow CH + O_2$	$k_{rev} \cdot K_{eq}$	b
514	$CO + HCHO \rightarrow CH_2 + CO_2$	$k_{rev} \cdot K_{eq}$	b
515	$CO + HCO \rightarrow CH + CO_2$	$k_{rev} \cdot K_{eq}$	b
516	$CH_3O + H \rightarrow CH_3 + OH$	$k_{rev} \cdot K_{eq}$	b
517	$H_2 + HCHO \rightarrow CH_3 + OH$	$k_{rev} \cdot K_{eq}$	b
518	$CH_3O + OH \rightarrow CH_3 + HO_2$	$k_{rev} \cdot K_{eq}$	b
519	$H + HCHO \rightarrow CH_2 + OH$	$k_{rev} \cdot K_{eq}$	b
520	$HCHO + OH \rightarrow CH_2 + HO_2$	$k_{rev} \cdot K_{eq}$	b
521	$CH_3 + HO_2 \rightarrow CH_2 + H_2O_2$	$k_{rev} \cdot K_{eq}$	b
522	$C + H_2O \rightarrow CH + OH$	$k_{rev} \cdot K_{eq}$	b
523	$H + HCO \rightarrow CH + OH$	$k_{rev} \cdot K_{eq}$	b
524	$H + HCHO \rightarrow CH + H_2O$	$k_{rev} \cdot K_{eq}$	b
525	$CO_2 + HCO \rightarrow CO + COOH$	$k_{rev} \cdot K_{eq}$	b
526	$CH_3 + CO_2 \rightarrow CH_3O + CO$	$k_{rev} \cdot K_{eq}$	b
527	$HCHO + HCO \rightarrow CH_3O + CO$	$k_{rev} \cdot K_{eq}$	b
528	$CH_3O + CO_2 \rightarrow CH_3OO + CO$	$k_{rev} \cdot K_{eq}$	b
529	$CO + H_2 \rightarrow H + HCO$	$k_{rev} \cdot K_{eq}$	b

#	Reaction	Rate equation	Ref.
530	$CH_2 + O \rightarrow H + HCO$	$k_{rev} \cdot K_{eq}$	b
531	$H_2 + HCO \rightarrow H + HCHO$	$k_{rev} \cdot K_{eq}$	b
532	$CH_3O \rightarrow H + HCHO$	$k_{rev} \cdot K_{eq}$	b
533	$H_2 + HCHO \rightarrow CH_3O + H$	$k_{rev} \cdot K_{eq}$	b
534	$CH_3OH \rightarrow CH_3O + H$	$k_{rev} \cdot K_{eq}$	b
535	$CH_3OH + H \rightarrow CH_3O + H_2$	$k_{rev} \cdot K_{eq}$	b
536	$H_2 + HCHO \rightarrow CH_2OH + H$	$k_{rev} \cdot K_{eq}$	b
537	$CH_3 + H_2O \rightarrow CH_3OH + H$	$k_{rev} \cdot K_{eq}$	b
538	$CH_3O + OH \rightarrow CH_3OO + H$	$k_{rev} \cdot K_{eq}$	b
539	$CH_3OOH + H \rightarrow CH_3OO + H_2$	$k_{rev} \cdot K_{eq}$	b
540	$CO + H_2O \rightarrow HCO + OH$	$k_{rev} \cdot K_{eq}$	b
541	$H + HCOOH \rightarrow HCHO + OH$	$k_{rev} \cdot K_{eq}$	b
542	$COOH + H_2O \rightarrow HCOOH + OH$	$k_{rev} \cdot K_{eq}$	b
543	$H_2O + HCHO \rightarrow CH_3O + OH$	$k_{rev} \cdot K_{eq}$	b
544	$H_2O_2 + HCHO \rightarrow CH_3O + HO_2$	$k_{rev} \cdot K_{eq}$	b
545	$CH_3OH + O_2 \rightarrow CH_3O + HO_2$	$k_{rev} \cdot K_{eq}$	b
546	$H_2O + HCHO \rightarrow CH_2OH + OH$	$k_{rev} \cdot K_{eq}$	b
547	$CH_3OH + OH \rightarrow CH_2OH + H_2O$	$k_{rev} \cdot K_{eq}$	b
548	$H_2O_2 + HCHO \rightarrow CH_2OH + HO_2$	$k_{rev} \cdot K_{eq}$	b
549	$H_2O + HCOOH \rightarrow CH_2OH + HO_2$	$k_{rev} \cdot K_{eq}$	b
550	$CH_3O + H_2O_2 \rightarrow CH_3OH + HO_2$	$k_{rev} \cdot K_{eq}$	b
551	$CH_3OO + H_2O \rightarrow CH_3OOH + OH$	$k_{rev} \cdot K_{eq}$	b
552	$CH_3OH + O_2 \rightarrow CH_3OO + OH$	$k_{rev} \cdot K_{eq}$	b
553	$CH_3OOH + O_2 \rightarrow CH_3OO + HO_2$	$k_{rev} \cdot K_{eq}$	b
554	$CH_3OOH + HO_2 \rightarrow CH_3OO + H_2O_2$	$k_{rev} \cdot K_{eq}$	b
555	$CO + OH \rightarrow HCO + O$	$k_{rev} \cdot K_{eq}$	b
556	$CO_2 + H \rightarrow HCO + O$	$k_{rev} \cdot K_{eq}$	b
557	$CO + HO_2 \rightarrow HCO + O_2$	$k_{rev} \cdot K_{eq}$	b
558	$HCO + OH \rightarrow HCHO + O$	$k_{rev} \cdot K_{eq}$	b
559	$HCO + HO_2 \rightarrow HCHO + O_2$	$k_{rev} \cdot K_{eq}$	b
560	$HCHO + OH \rightarrow CH_3O + O$	$k_{rev} \cdot K_{eq}$	b
561	$HCHO + HO_2 \rightarrow CH_3O + O_2$	$k_{rev} \cdot K_{eq}$	b
562	$CH_2OH + OH \rightarrow CH_3OH + O$	$k_{rev} \cdot K_{eq}$	b
563	$CH_3O + O_2 \rightarrow CH_3OO + O$	$k_{rev} \cdot K_{eq}$	b
564	$CH_4 + CO \rightarrow CH_3 + HCO$	$k_{rev} \cdot K_{eq}$	b
565	$CH_3 + CO \rightarrow CH_2 + HCO$	$k_{rev} \cdot K_{eq}$	b
566	$CH_2CO + H_2O \rightarrow CH_3 + COOH$	$k_{rev} \cdot K_{eq}$	b
567	$CH_4 + CO_2 \rightarrow CH_3 + COOH$	$k_{rev} \cdot K_{eq}$	b
568	$CH_3 + HCO \rightarrow CH_2 + HCHO$	$k_{rev} \cdot K_{eq}$	b
569	$CH_2CO + H \rightarrow CH + HCHO$	$k_{rev} \cdot K_{eq}$	b

#	Reaction	Rate equation	Ref.
570	$CH_4 + HCHO \rightarrow CH_3 + CH_3O$	$k_{rev} \cdot K_{eq}$	b
571	$CH_3 + HCHO \rightarrow CH_2 + CH_3O$	$k_{rev} \cdot K_{eq}$	b
572	$CH_4 + HCHO \rightarrow CH_2OH + CH_3$	$k_{rev} \cdot K_{eq}$	b
573	$C_2H_4 + OH \rightarrow CH_2 + CH_2OH$	$k_{rev} \cdot K_{eq}$	b
574	$CH_3 + HCHO \rightarrow CH_2 + CH_2OH$	$k_{rev} \cdot K_{eq}$	b
575	$CH_2OH + CH_3 \rightarrow CH_2 + CH_3OH$	$k_{rev} \cdot K_{eq}$	b
576	$CH_3 + CH_3O \rightarrow CH_2 + CH_3OH$	$k_{rev} \cdot K_{eq}$	b
577	$CH_3 + CH_3OOH \rightarrow CH_3OO + CH_4$	$k_{rev} \cdot K_{eq}$	b
578	$CH_3O + CH_3O \rightarrow CH_3 + CH_3OO$	$k_{rev} \cdot K_{eq}$	b
579	$CH_3O + HCHO \rightarrow CH_2 + CH_3OO$	$k_{rev} \cdot K_{eq}$	b
580	$C_2H_5 + O_2 \rightarrow CH_2 + CH_3OO$	$k_{rev} \cdot K_{eq}$	b
581	$CO + HCHO \rightarrow HCO + HCO$	$k_{rev} \cdot K_{eq}$	b
582	$CH_3OH + CO \rightarrow CH_3O + HCO$	$k_{rev} \cdot K_{eq}$	b
583	$CH_3OH + CO \rightarrow CH_2OH + HCO$	$k_{rev} \cdot K_{eq}$	b
584	$HCHO + HCHO \rightarrow CH_2OH + HCO$	$k_{rev} \cdot K_{eq}$	b
585	$CH_3OH + HCHO \rightarrow CH_3O + CH_3O$	$k_{rev} \cdot K_{eq}$	b
586	$CH_3OH + HCHO \rightarrow CH_2OH + CH_3O$	$k_{rev} \cdot K_{eq}$	b
587	$CH_3OH + HCHO \rightarrow CH_2OH + CH_2OH$	$k_{rev} \cdot K_{eq}$	b
588	$CH_3OOH + HCHO \rightarrow CH_3O + CH_3OO$	$k_{rev} \cdot K_{eq}$	b
589	$CH_2OH + CH_3OOH \rightarrow CH_3OH + CH_3OO$	$k_{rev} \cdot K_{eq}$	b
590	$CH_3O + CH_3OOH \rightarrow CH_3OH + CH_3OO$	$k_{rev} \cdot K_{eq}$	b
591	$CH_3OOH + HCHO \rightarrow CH_2OH + CH_3OO$	$k_{rev} \cdot K_{eq}$	b
592	$CH_3OH + HCOOH \rightarrow CH_2OH + CH_3OO$	$k_{rev} \cdot K_{eq}$	b
593	$CH_3OOH + HCO \rightarrow CH_3OO + HCHO$	$k_{rev} \cdot K_{eq}$	b
594	$C_2H_4 + H_2O \rightarrow C_2H_5 + OH$	$k_{rev} \cdot K_{eq}$	b
595	$C_2H_4 + H_2O_2 \rightarrow C_2H_5 + HO_2$	$k_{rev} \cdot K_{eq}$	b
596	$CH_3 + HCHO \rightarrow C_2H_4 + OH$	$k_{rev} \cdot K_{eq}$	b
597	$CH_3CHO + H \rightarrow C_2H_4 + OH$	$k_{rev} \cdot K_{eq}$	b
598	$CH_2CH_2OH \rightarrow C_2H_4 + OH$	$k_{rev} \cdot K_{eq}$	b
599	$CH_3 + HCO \rightarrow C_2H_3 + OH$	$k_{rev} \cdot K_{eq}$	b
600	$CH_3CO + H \rightarrow C_2H_3 + OH$	$k_{rev} \cdot K_{eq}$	b
601	$C_2H_2 + H_2O \rightarrow C_2H_3 + OH$	$k_{rev} \cdot K_{eq}$	b
602	$CH_2CO + H_2 \rightarrow C_2H_3 + OH$	$k_{rev} \cdot K_{eq}$	b
603	$CH_4 + CO \rightarrow C_2H_3 + OH$	$k_{rev} \cdot K_{eq}$	b
604	$C_2H_4 + OH \rightarrow C_2H_3 + H_2O$	$k_{rev} \cdot K_{eq}$	b
605	$C_2H_4 + HO_2 \rightarrow C_2H_3 + H_2O_2$	$k_{rev} \cdot K_{eq}$	b
606	$CH_2CO + H \rightarrow C_2H_2 + OH$	$k_{rev} \cdot K_{eq}$	b
607	$C_2H_2 + O \rightarrow C_2H + OH$	$k_{rev} \cdot K_{eq}$	b
608	$CH_2 + CO \rightarrow C_2H + OH$	$k_{rev} \cdot K_{eq}$	b
609	$C_2H_2 + O_2 \rightarrow C_2H + HO_2$	$k_{rev} \cdot K_{eq}$	b

#	Reaction	Rate equation	Ref.
610	$HCCO + OH \rightarrow C_2H + HO_2$	$k_{rev} \cdot K_{eq}$	b
611	$CH_3CHO + H \rightarrow C_2H_5 + O$	$k_{rev} \cdot K_{eq}$	b
612	$CH_3 + HCHO \rightarrow C_2H_5 + O$	$k_{rev} \cdot K_{eq}$	b
613	$C_2H_4 + OH \rightarrow C_2H_5 + O$	$k_{rev} \cdot K_{eq}$	b
614	$CH_3 + HCO \rightarrow C_2H_4 + O$	$k_{rev} \cdot K_{eq}$	b
615	$CH_2CO + H_2 \rightarrow C_2H_4 + O$	$k_{rev} \cdot K_{eq}$	b
616	$C_2H_3 + HO_2 \rightarrow C_2H_4 + O_2$	$k_{rev} \cdot K_{eq}$	b
617	$C_2H_2 + OH \rightarrow C_2H_3 + O$	$k_{rev} \cdot K_{eq}$	b
618	$CH_3 + CO \rightarrow C_2H_3 + O$	$k_{rev} \cdot K_{eq}$	b
619	$CH_2 + HCO \rightarrow C_2H_3 + O$	$k_{rev} \cdot K_{eq}$	b
620	$HCHO + HCO \rightarrow C_2H_3 + O_2$	$k_{rev} \cdot K_{eq}$	b
621	$CH_2 + CO \rightarrow C_2H_2 + O$	$k_{rev} \cdot K_{eq}$	b
622	$H + HCCO \rightarrow C_2H_2 + O$	$k_{rev} \cdot K_{eq}$	b
623	$HCO + HCO \rightarrow C_2H_2 + O_2$	$k_{rev} \cdot K_{eq}$	b
624	$CH + CO \rightarrow C_2H + O$	$k_{rev} \cdot K_{eq}$	b
625	$CO + HCO \rightarrow C_2H + O_2$	$k_{rev} \cdot K_{eq}$	b
626	$CH + CO_2 \rightarrow C_2H + O_2$	$k_{rev} \cdot K_{eq}$	b
627	$C_2H_5 + CH_3OOH \rightarrow C_2H_6 + CH_3OO$	$k_{rev} \cdot K_{eq}$	b
628	$C_2H_6 + CO \rightarrow C_2H_5 + HCO$	$k_{rev} \cdot K_{eq}$	b
629	$C_2H_6 + HCHO \rightarrow C_2H_5 + CH_3O$	$k_{rev} \cdot K_{eq}$	b
630	$C_2H_4 + CH_3OH \rightarrow C_2H_5 + CH_2OH$	$k_{rev} \cdot K_{eq}$	b
631	$C_2H_6 + HCHO \rightarrow C_2H_5 + CH_2OH$	$k_{rev} \cdot K_{eq}$	b
632	$CH_3CH_2O + CH_3O \rightarrow C_2H_5 + CH_3OO$	$k_{rev} \cdot K_{eq}$	b
633	$C_2H_5 + CO_2 \rightarrow C_2H_4 + COOH$	$k_{rev} \cdot K_{eq}$	b
634	$C_2H_5 + HCHO \rightarrow C_2H_4 + CH_2OH$	$k_{rev} \cdot K_{eq}$	b
635	$C_2H_4 + HCO \rightarrow C_2H_3 + HCHO$	$k_{rev} \cdot K_{eq}$	b
636	$C_2H_4 + HCHO \rightarrow C_2H_3 + CH_3O$	$k_{rev} \cdot K_{eq}$	b
637	$C_2H_4 + HCHO \rightarrow C_2H_3 + CH_2OH$	$k_{rev} \cdot K_{eq}$	b
638	$C_2H_4 + CH_2OH \rightarrow C_2H_3 + CH_3OH$	$k_{rev} \cdot K_{eq}$	b
639	$C_2H_4 + CH_3O \rightarrow C_2H_3 + CH_3OH$	$k_{rev} \cdot K_{eq}$	b
640	$C_2H_3 + CO_2 \rightarrow C_2H_2 + COOH$	$k_{rev} \cdot K_{eq}$	b
641	$C_2H_3 + HCHO \rightarrow C_2H_2 + CH_2OH$	$k_{rev} \cdot K_{eq}$	b
642	$C_2H_2 + HCHO \rightarrow C_2H + CH_3O$	$k_{rev} \cdot K_{eq}$	b
643	$C_2H_2 + HCHO \rightarrow C_2H + CH_2OH$	$k_{rev} \cdot K_{eq}$	b
644	$C_2H_2 + CH_2OH \rightarrow C_2H + CH_3OH$	$k_{rev} \cdot K_{eq}$	b
645	$C_2H_2 + CH_3O \rightarrow C_2H + CH_3OH$	$k_{rev} \cdot K_{eq}$	b
646	$CH_3O + HCCO \rightarrow C_2H + CH_3OO$	$k_{rev} \cdot K_{eq}$	b
647	$CH_2 + CO \rightarrow H + HCCO$	$k_{rev} \cdot K_{eq}$	b
648	$CH_3 + CO \rightarrow CH_2CO + H$	$k_{rev} \cdot K_{eq}$	b
649	$H_2 + HCCO \rightarrow CH_2CO + H$	$k_{rev} \cdot K_{eq}$	b

#	Reaction	Rate equation	Ref.
650	$CH_3 + HCO \rightarrow CH_3CO + H$	$k_{rev} \cdot K_{eq}$	[b]
651	$CH_2CO + H_2 \rightarrow CH_3CO + H$	$k_{rev} \cdot K_{eq}$	[b]
652	$CH_2OH + CH_3 \rightarrow CH_3CH_2O + H$	$k_{rev} \cdot K_{eq}$	[b]
653	$CH_3CH_2OH \rightarrow CH_3CH_2O + H$	$k_{rev} \cdot K_{eq}$	[b]
654	$C_2H_5 + OH \rightarrow CH_3CH_2O + H$	$k_{rev} \cdot K_{eq}$	[b]
655	$C_2H_4 + H_2O \rightarrow CH_3CH_2O + H$	$k_{rev} \cdot K_{eq}$	[b]
656	$CH_3CHO + H_2 \rightarrow CH_3CH_2O + H$	$k_{rev} \cdot K_{eq}$	[b]
657	$CH_4 + HCHO \rightarrow CH_3CH_2O + H$	$k_{rev} \cdot K_{eq}$	[b]
658	$CH_3CH_2OH \rightarrow CH_3CHOH + H$	$k_{rev} \cdot K_{eq}$	[b]
659	$CH_2OH + CH_3 \rightarrow CH_3CHOH + H$	$k_{rev} \cdot K_{eq}$	[b]
660	$C_2H_5 + OH \rightarrow CH_3CHOH + H$	$k_{rev} \cdot K_{eq}$	[b]
661	$C_2H_4 + H_2O \rightarrow CH_3CHOH + H$	$k_{rev} \cdot K_{eq}$	[b]
662	$CH_3CHO + H_2 \rightarrow CH_3CHOH + H$	$k_{rev} \cdot K_{eq}$	[b]
663	$CH_4 + HCHO \rightarrow CH_3CHOH + H$	$k_{rev} \cdot K_{eq}$	[b]
664	$C_2H_5 + H_2O \rightarrow CH_3CH_2OH + H$	$k_{rev} \cdot K_{eq}$	[b]
665	$CH_3CHOH + H_2 \rightarrow CH_3CH_2OH + H$	$k_{rev} \cdot K_{eq}$	[b]
666	$CH_2CH_2OH + H_2 \rightarrow CH_3CH_2OH + H$	$k_{rev} \cdot K_{eq}$	[b]
667	$CH_3CH_2O + H_2 \rightarrow CH_3CH_2OH + H$	$k_{rev} \cdot K_{eq}$	[b]
668	$CH_2OH + CO \rightarrow CH_2CO + OH$	$k_{rev} \cdot K_{eq}$	[b]
669	$H_2O + HCCO \rightarrow CH_2CO + OH$	$k_{rev} \cdot K_{eq}$	[b]
670	$HCHO + HCO \rightarrow CH_2CO + OH$	$k_{rev} \cdot K_{eq}$	[b]
671	$CH_3 + CO_2 \rightarrow CH_2CO + OH$	$k_{rev} \cdot K_{eq}$	[b]
672	$CH_2CO + H_2O \rightarrow CH_3CO + OH$	$k_{rev} \cdot K_{eq}$	[b]
673	$CH_3CO + H_2O \rightarrow CH_3CHO + OH$	$k_{rev} \cdot K_{eq}$	[b]
674	$CH_3 + HCOOH \rightarrow CH_3CHO + OH$	$k_{rev} \cdot K_{eq}$	[b]
675	$CH_3COOH + H \rightarrow CH_3CHO + OH$	$k_{rev} \cdot K_{eq}$	[b]
676	$CH_2CH_2OH + H_2O \rightarrow CH_3CH_2OH + OH$	$k_{rev} \cdot K_{eq}$	[b]
677	$CH_3CHOH + H_2O \rightarrow CH_3CH_2OH + OH$	$k_{rev} \cdot K_{eq}$	[b]
678	$CH_3CH_2O + H_2O \rightarrow CH_3CH_2OH + OH$	$k_{rev} \cdot K_{eq}$	[b]
679	$CH_3CHOH + H_2O_2 \rightarrow CH_3CH_2OH + HO_2$	$k_{rev} \cdot K_{eq}$	[b]
680	$CH + CO_2 \rightarrow HCCO + O$	$k_{rev} \cdot K_{eq}$	[b]
681	$CO + HCHO \rightarrow CH_2CO + O$	$k_{rev} \cdot K_{eq}$	[b]
682	$HCO + HCO \rightarrow CH_2CO + O$	$k_{rev} \cdot K_{eq}$	[b]
683	$CH_2 + CO_2 \rightarrow CH_2CO + O$	$k_{rev} \cdot K_{eq}$	[b]
684	$CH_2CO + OH \rightarrow CH_3CO + O$	$k_{rev} \cdot K_{eq}$	[b]
685	$CH_3 + CO_2 \rightarrow CH_3CO + O$	$k_{rev} \cdot K_{eq}$	[b]
686	$CH_3CO + OH \rightarrow CH_3CHO + O$	$k_{rev} \cdot K_{eq}$	[b]
687	$CH_3CO + HO_2 \rightarrow CH_3CHO + O_2$	$k_{rev} \cdot K_{eq}$	[b]
688	$CH_3CHO + HO_2 \rightarrow CH_3CH_2O + O_2$	$k_{rev} \cdot K_{eq}$	[b]
689	$CH_3 + HCOOH \rightarrow CH_3CHOH + O$	$k_{rev} \cdot K_{eq}$	[b]

#	Reaction	Rate equation	Ref.
690	$CH_3CHO + OH \rightarrow CH_3CHOH + O$	$k_{rev} \cdot K_{eq}$	b
691	$CH_3COOH + H \rightarrow CH_3CHOH + O$	$k_{rev} \cdot K_{eq}$	b
692	$CH_3CHO + HO_2 \rightarrow CH_3CHOH + O_2$	$k_{rev} \cdot K_{eq}$	b
693	$CH_2OH + HCHO \rightarrow CH_2CH_2OH + O$	$k_{rev} \cdot K_{eq}$	b
694	$CH_3CHOH + OH \rightarrow CH_3CH_2OH + O$	$k_{rev} \cdot K_{eq}$	b
695	$CH_2CH_2OH + OH \rightarrow CH_3CH_2OH + O$	$k_{rev} \cdot K_{eq}$	b
696	$CH_3CH_2O + OH \rightarrow CH_3CH_2OH + O$	$k_{rev} \cdot K_{eq}$	b
697	$CH_3CHOH + HO_2 \rightarrow CH_3CH_2OH + O_2$	$k_{rev} \cdot K_{eq}$	b
698	$CH_2CH_2OH + HO_2 \rightarrow CH_3CH_2OH + O_2$	$k_{rev} \cdot K_{eq}$	b
699	$CH_3CH_2O + HO_2 \rightarrow CH_3CH_2OH + O_2$	$k_{rev} \cdot K_{eq}$	b
700	$C_2H_5 + CO \rightarrow CH_2CO + CH_3$	$k_{rev} \cdot K_{eq}$	b
701	$CH_4 + HCCO \rightarrow CH_2CO + CH_3$	$k_{rev} \cdot K_{eq}$	b
702	$C_2H_4 + CO \rightarrow CH_2 + CH_2CO$	$k_{rev} \cdot K_{eq}$	b
703	$CH_3 + HCCO \rightarrow CH_2 + CH_2CO$	$k_{rev} \cdot K_{eq}$	b
704	$CH_2CO + CH_4 \rightarrow CH_3 + CH_3CO$	$k_{rev} \cdot K_{eq}$	b
705	$CH_2CO + CH_3 \rightarrow CH_2 + CH_3CO$	$k_{rev} \cdot K_{eq}$	b
706	$CH_3CHOH + CH_4 \rightarrow CH_3 + CH_3CH_2OH$	$k_{rev} \cdot K_{eq}$	b
707	$CH_2CH_2OH + CH_4 \rightarrow CH_3 + CH_3CH_2OH$	$k_{rev} \cdot K_{eq}$	b
708	$CH_3CH_2O + CH_4 \rightarrow CH_3 + CH_3CH_2OH$	$k_{rev} \cdot K_{eq}$	b
709	$CH_3CHO + CO \rightarrow CH_3CO + HCO$	$k_{rev} \cdot K_{eq}$	b
710	$CH_3CHO + HCO \rightarrow CH_3CO + HCHO$	$k_{rev} \cdot K_{eq}$	b
711	$CH_2CO + CH_3OH \rightarrow CH_3CO + CH_3O$	$k_{rev} \cdot K_{eq}$	b
712	$CH_3CHO + HCHO \rightarrow CH_3CO + CH_3O$	$k_{rev} \cdot K_{eq}$	b
713	$CH_2OH + CH_3CHO \rightarrow CH_3CO + CH_3OH$	$k_{rev} \cdot K_{eq}$	b
714	$CH_3CO + CH_3OH \rightarrow CH_3CHO + CH_3O$	$k_{rev} \cdot K_{eq}$	b
715	$CH_3CO + CH_3OOH \rightarrow CH_3CHO + CH_3OO$	$k_{rev} \cdot K_{eq}$	b
716	$CH_2CO + CH_3CHO \rightarrow CH_3CO + CH_3CO$	$k_{rev} \cdot K_{eq}$	b
717	$CO + OH \rightarrow COOH$	$k_{rev} \cdot K_{eq}$	b
718	$CO_2 + H \rightarrow COOH$	$k_{rev} \cdot K_{eq}$	b
719	$H + HCO \rightarrow HCHO$	$k_{rev} \cdot K_{eq}$	b
720	$H + HCHO \rightarrow CH_2OH$	$k_{rev} \cdot K_{eq}$	b
721	$CH_2 + H_2O \rightarrow CH_3OH$	$k_{rev} \cdot K_{eq}$	b
722	$CH_2OH + H \rightarrow CH_3OH$	$k_{rev} \cdot K_{eq}$	b
723	$CH_3O + OH \rightarrow CH_3OOH$	$k_{rev} \cdot K_{eq}$	b
724	$CH_3 + COOH \rightarrow CH_3COOH$	$k_{rev} \cdot K_{eq}$	b
725	$CH_3 + HCHO \rightarrow CH_3CHOH$	$k_{rev} \cdot K_{eq}$	b
726	$CO + O \rightarrow C + O_2$	$k_{rev} \cdot K_{eq}$	b
727	$CO_2 + O_2 \rightarrow CO + O_3$	$k_{rev} \cdot K_{eq}$	b
728	$CO + CO \rightarrow C + CO_2$	$k_{rev} \cdot K_{eq}$	b

Constants:

$N_A = 6.02214076 \times 10^{23} mol^{-1}$

$k_B = 1.38064852 \times 10^{-23} J/K$

$R = 8.31446261815324 JK^{-1} mol^{-1}$

n_M = total number density of neutral species (cm^{-3})

Notes:

[a] falloff expression, Lindemann-Hinshelwood expression with broadening factor:

$k = \frac{k_0[M]k_\infty}{k_0[M]+k_\infty} F$; $logF = \frac{logF_c}{1+\left[\frac{log(k_0[M]/k_\infty)}{N}\right]^2}$; $N = 0.75 - 1.27 logF_c$

[b] reaction rate expression calculated from equilibrium constant and reverse reaction rate:

$K_{eq} = e^{\left(\frac{-\Delta G_r}{RT}\right)} \cdot \left(\frac{p}{R \cdot T}\right)^{\Delta v}$; $p = 1bar$; $\Delta v = \sum \mu_P - \sum \mu_R$

References

(1) B. J. McBride, M. J. Zehe and S. Gordon, *John H. Glenn Research Center at Lewis Field*, 2002, 295.

(2) A. Burcat, B. Ruscic and Chemistry, *Third millenium ideal gas and condensed phase thermochemical database for combustion (with update from active thermochemical tables)*. Tech. rep., Argonne National Laboratory (ANL), Argonne, IL, 2005, DOI: 10.2172/925269.

(3) E. A. Mason and S. C. Saxena, *The Physics of Fluids*, 1958, **1**, 361–369, DOI: 10.1063/1.1724352.

(4) B. J. McBride, S. Gordon and M. A. Reno, *Coefficients for calculating thermodynamic and transport properties of individual species*, tech. rep., 1993, p. 96.

(5) J. Slaets, B. Loenders and A. Bogaerts, *Fuel*, 2024, **360**, 130650, DOI: 10.1016/j.fuel.2023.130650.

(6) R. Snoeckx and A. Bogaerts, *Chemical Society Reviews*, 2017, **46**, 5805–5863, DOI: 10.1039/C6CS00066E.

(7) M. J. Rabinowitz, J. W. Sutherland, P. M. Patterson and R. B. Klemm, *The Journal of Physical Chemistry*, 1991, **95**, 674–681, DOI: 10.1021/j100155a033.

(8) D. L. Baulch, C. T. Bowman, C. J. Cobos, R. A. Cox, T. Just, J. A. Kerr, M. J. Pilling, D. Stocker, J. Troe, W. Tsang, R. W. Walker and J. Warnatz, 2005, **34**, 757–1397, DOI: 10.1063/1.1748524.

(9) K.-W. Lu, H. Matsui, C.-L. Huang, P. Raghunath, N.-S. Wang and M. C. Lin, 2010, **114**, 5493–5502, DOI: 10.1021/jp100535r.

(10) W. Braun, A. M. Bass, D. D. Davis, J. D. Simmons, G. Porter and M. J. Lighthill, *Proceedings of the Royal Society of London. A. Mathematical and Physical Sciences*, 1969, **312**, 417–434, DOI: 10.1098/rspa.1969.0168.

(11) A. J. Dean and R. K. Hanson, 1992, **24**, 517–532, DOI: 10.1002/kin.550240602.

(12) K. Tabayashi and S. Bauer, 1979, **34**, 63–83, DOI: 10.1016/0010-2180(79)90079-8.

(13) T. Bohland, S. Dobe, F. Temps and H. G. Wagner, 1985, **89**, 1110–1116, DOI: 10.1002/bbpc.19850891018.

(14) P. Han, K. Su, Y. Liu, Y. Wang, X. Wang, Q. Zeng, L. Cheng and L. Zhang, 2011, **32**, 2745–2755, DOI: 10.1002/jcc.21854.

(15) S. Bauerle, M. Klatt and H. G. G. Wagner, 1995, **99**, 870–879, DOI: 10.1002/bbpc.19950990612.

(16) W. Braun, J. R. McNesby and A. M. Bass, 1967, **46**, 2071–2080, DOI: 10.1063/1.1841003.

(17) W. Tsang and R. F. Hampson, 1986, **15**, 1087–1279, DOI: 10.1063/1.555759.

(18) V. D. Knyazev, A. Bencsura, S. I. Stoliarov and I. R. Slagle, *The Journal of Physical Chemistry*, 1996, **100**, 11346–11354, DOI: 10.1021/jp9606568.

(19) M. Mandal, S. Ghosh and B. Maiti, *The Journal of Physical Chemistry A*, 2018, **122**, 3556–3562, DOI: 10.1021/acs.jpca.8b01386.

(20) A. Bergeat and J.-C. Loison, *Physical Chemistry Chemical Physics*, 2001, **3**, 2038–2042, DOI: 10.1039/b100656h.

(21) N. Galland, F. Caralp, Y. Hannachi, A. Bergeat and J.-C. Loison, *The Journal of Physical Chemistry A*, 2003, **107**, 5419–5426, DOI: 10.1021/jp027465r.

(22) Y. Ge, M. S. Gordon, F. Battaglia and R. O. Fox, *The Journal of Physical Chemistry A*, 2010, **114**, 2384–2392, DOI: 10.1021/jp911673h.

(23) S. I. Stoliarov, V. D. Knyazev and I. R. Slagle, *The Journal of Physical Chemistry A*, 2000, **104**, 9687–9697, DOI: 10.1021/jp992476e.

(24) T. Ibuki and Y. Takezaki, *Bulletin of the Chemical Society of Japan*, 1975, **48**, 769–773, DOI: 10.1246/bcsj.48.769.

(25) A. Fahr and D. C. Tardy, *The Journal of Physical Chemistry A*, 2002, **106**, 11135–11140, DOI: 10.1021/jp021497x.

(26) M. R. Dash and B. Rajakumar, *Physical Chemistry Chemical Physics*, 2015, **17**, 3142–3156, DOI: 10.1039/c4cp04677c.

(27) P. Stewart, C. Larson and D. Golden, *Combustion and Flame*, 1989, **75**, 25–31, DOI: 10.1016/0010-2180(89)90084-9.

(28) A. M. Dean, *The Journal of Physical Chemistry*, 1985, **89**, 4600–4608, DOI: 10.1021/j100267a038.

(29) M. Capitelli, C. M. Ferreira, B. F. Gordiets and A. I. Osipov, Springer Berlin Heidelberg, 2000, DOI: 10.1007/978-3-662-04158-1.

(30) S. P. Karkach and V. I. Osherov, *The Journal of Chemical Physics*, 1999, **110**, 11918–11927, DOI: 10.1063/1.479131.

(31) R. Atkinson, D. L. Baulch, R. A. Cox, J. N. Crowley, R. F. Hampson, R. G. Hynes, M. E. Jenkin, M. J. Rossi and J. Troe, *Atmospheric Chemistry and Physics*, 2004, **4**, 1461–1738, DOI: 10.5194/acp-4-1461-2004.

(32) D. L. Baulch, C. J. Cobos, R. A. Cox, P. Frank, G. Hayman, T. Just, J. A. Kerr, T. Murrells, M. J. Pilling, J. Troe, R. W. Walker and J. Warnatz, *Journal of Physical and Chemical Reference Data*, 1994, **23**, 847–848, DOI: 10.1063/1.555953.

(33) R. Atkinson, D. L. Baulch, R. A. Cox, R. F. Hampson, J. A. K. (Chairman) and J. Troe, *Journal of Physical and Chemical Reference Data*, 1989, **18**, 881–1097, DOI: 10.1063/1.555832.

(34) C. J. Howard and B. J. Finlayson-Pitts, *The Journal of Chemical Physics*, 1980, **72**, 3842–3843, DOI: 10.1063/1.439601.

(35) P. Glarborg, J. A. Miller, B. Ruscic and S. J. Klippenstein, 2018, **67**, 31–68, DOI: 10.1016/j.pecs.2018.01.002.

(36) V. Lissianski, H. Yang, Z. Qin, M. Mueller, K. Shin and W. Gardiner, *Chemical Physics Letters*, 1995, **240**, 57–62, DOI: 10.1016/0009-2614(95)00496-q.

(37) P. Glarborg, M. U. Alzueta, K. Dam-Johansen and J. A. Miller, 1998, **115**, 1–27, DOI: 10.1016/s0010-2180(97)00359-3.

(38) P. Glarborg and P. Marshall, 2009, **475**, 40–43, DOI: 10.1016/j.cplett.2009.05.028.

(39) G. Bogdanchikov, A. Baklanov and D. Parker, 2004, **385**, 486–490, DOI: 10.1016/j.cplett.2004.01.015.

(40) C. Dombrowsky, S. M. Hwang, M. Rohrig and H. G. Wagner, 1992, **96**, 194–198, DOI: 10.1002/bbpc.19920960215.

(41) N. K. Srinivasan, M.-C. Su, J. W. Sutherland and J. V. Michael, 2005, **109**, 1857–1863, DOI: 10.1021/jp040679j.

(42) N. Cohen and K. R. Westberg, 1991, **20**, 1211–1311, DOI: 10.1063/1.555901.

(43) N. Cohen and K. R. Westberg, 1983, **12**, 531–590, DOI: 10.1063/1.555692.

(44) J. T. Petty, J. A. Harrison and C. B. Moore, 1993, **97**, 11194–11198, DOI: 10.1021/j100145a013.

(45) B. Wang, H. Hou and Y. Gu, 1999, **103**, 8021–8029, DOI: 10.1021/jp991203g.

(46) S. P. Sander and R. T. Watson, 1980, **84**, 1664–1674, DOI: 10.1021/j100450a002.

(47) T. Tsuboi and K. Hashimoto, 1981, **42**, 61–76, DOI: 10.1016/0010-2180(81)90142-5.

(48) H. J. Curran, 2006, **38**, 250–275, DOI: 10.1002/kin.20153.

(49) K. Brudnik, A. A. Gola and J. T. Jodkowski, 2009, **15**, 1061–1066, DOI: 10.1007/s00894-009-0461-x.

(50) J. T. Jodkowski, M.-T. Rayez, J.-C. Rayez, T. Berces and S. Dobe, 1999, **103**, 3750–3765, DOI: 10.1021/jp984367q.

(51) W. Tsang, 1987, **16**, 471–508, DOI: 10.1063/1.555802.

(52) Y. Hidaka, T. Oki, H. Kawano and T. Higashihara, 1989, **93**, 7134–7139, DOI: 10.1021/j100357a022.

(53) R. A. Yetter, H. Rabitz, F. L. Dryer, R. G. Maki and R. B. Klemm, 1989, **91**, 4088–4097, DOI: 10.1063/1.456838.

(54) A. Galano, J. R. Alvarez-Idaboy, M. E. Ruiz-Santoyo and A. Vivier-Bunge, 2002, **106**, 9520–9528, DOI: 10.1021/jp020297i.

(55) E. Assaf, C. Schoemaecker, L. Vereecken and C. Fittschen, 2018, **20**, 10660–10670, DOI: 10.1039/c7cp05770a.

(56) S. H. Mousavipour and Z. Homayoon, 2011, **115**, 3291–3300, DOI: 10.1021/jp112081r.

(57) M. Altarawneh, A. H. Al-Muhtaseb, B. Z. Dlugogorski, E. M. Kennedy and J. C. Mackie, 2011, **32**, 1725–1733, DOI: 10.1002/jcc.21756.

(58) H.-G. Yu and J. S. Francisco, 2009, **113**, 3844–3849, DOI: 10.1021/jp809730j.

(59) H. M. T. Nguyen, H. T. Nguyen, T.-N. Nguyen, H. V. Hoang and L. Vereecken, 2014, **118**, 8861–8871, DOI: 10.1021/jp506175k.

(60) Z. Zhao, J. Song, B. Su, X. Wang and Z. Li, 2018, **122**, 5078–5088, DOI: 10.1021/acs.jpca.7b09988.

(61) F. Xiao, X. Sun, Z. Li and X. Li, *ACS Omega*, 2020, **5**, 12777–12788, DOI: 10.1021/acsomega.0c00400.

(62) V. D. Knyazev, 2017, **685**, 165–170, DOI: 10.1016/j.cplett.2017.07.040.

(63) A. M. Mebel, E. W. G. Diau, M. C. Lin and K. Morokuma, 1996, **118**, 9759–9771, DOI: 10.1021/ja961476e.

(64) S. A. Carl, H. M. T. Nguyen, R. M. I. Elsamra, M. T. Nguyen and J. Peeters, 2005, **122**, 114307, DOI: 10.1063/1.1861887.

(65) Y. Hidaka, T. Nishimori, K. Sato, Y. Henmi, R. Okuda, K. Inami and T. Higashihara, 1999, **117**, 755–776, DOI: 10.1016/s0010-2180(98)00128-x.

(66) J. Gimenez-Lopez, C. T. Rasmussen, H. Hashemi, M. U. Alzueta, Y. Gao, P. Marshall, C. F. Goldsmith and P. Glarborg, 2016, **48**, 724–738, DOI: 10.1002/kin.21028.

(67) P.-C. Nam, P. Raghunath, L. K. Huynh, S. Xu and M. C. Lin, 2016, **188**, 1095–1114, DOI: 10.1080/00102202.2016.1151878.

(68) Y. Hidaka, K. Kimura and H. Kawano, 1994, **99**, 18–28, DOI: 10.1016/0010-2180(94)90079-5.

(69) L. K. Huynh and A. Violi, 2007, **73**, 94–101, DOI: 10.1021/jo701824n.

(70) K. Ohmori, A. Miyoshi, H. Matsui and N. Washida, 1990, **94**, 3253–3255, DOI: 10.1021/j100371a006.

(71) J. Warnatz, in Springer New York, 1984, pp. 197–360, DOI: 10.1007/978-1-4684-0186-8_5.

(72) R. Sivaramakrishnan, J. V. Michael and S. J. Klippenstein, 2009, **114**, 755–764, DOI: 10.1021/jp906918z.

(73) Z. F. Xu, K. Xu and M. C. Lin, 2011, **115**, 3509–3522, DOI: 10.1021/jp110580r.

(74) W.-K. Aders and H. G. Wagner, 1973, **77**, 332–335, DOI: https://doi.org/10.1002/bbpc.19730770509.

(75) R. Sivaramakrishnan, M.-C. Su, J. V. Michael, S. J. Klippenstein, L. B. Harding and B. Ruscic, 2010, **114**, 9425–9439, DOI: 10.1021/jp104759d.

(76) S.-Z. Xiong, Q. Yao, Z.-R. Li and X.-Y. Li, 2014, **161**, 885–897, DOI: 10.1016/j.combustflame.2013.10.013.

(77) M. Cameron, V. Sivakumaran, T. J. Dillon and J. N. Crowley, 2002, **4**, 3628–3638, DOI: 10.1039/b202586h.

(78) N. M. Marinov, 1999, **31**, 183–220, DOI: 10.1002/(sici)1097-4601(1999)31:3<183::aid-kin3>3.0.co;2-x.

(79) C. Olm, T. Varga, E. Valko, S. Hartl, C. Hasse and T. Turanyi, 2016, **48**, 423–441, DOI: 10.1002/kin.20998.

(80) D. Skouteris, N. Balucani, C. Ceccarelli, F. Vazart, C. Puzzarini, V. Barone, C. Codella and B. Lefloch, 2018, **854**, 135, DOI: 10.3847/1538-4357/aaa41e.

(81) G. da Silva, J. W. Bozzelli, L. Liang and J. T. Farrell, 2009, **113**, 8923–8933, DOI: 10.1021/jp903210a.

(82) A. S. Semenikhin, E. G. Shubina, A. S. Savchenkova, I. V. Chechet, S. G. Matveev, A. A. Konnov and A. M. Mebel, 2018, **50**, 273–284, DOI: 10.1002/kin.21156.

(83) P. Frank, K. A. Bhaskaran and T. Just, 1986, **90**, 2226–2231, DOI: 10.1021/j100401a046.

(84) E. Hassinen, K. Kalliorinne and J. Koskikallio, 1990, **22**, 741–745, DOI: 10.1002/kin.550220709.

(85) G. Hoehlein and G. R. Freeman, 1970, **92**, 6118–6125, DOI: 10.1021/ja00724a004.

(86) X. Zhang, L. Ye, Y. Li, Y. Zhang, C. Cao, J. Yang, Z. Zhou, Z. Huang and F. Qi, 2018, **191**, 431–441, DOI: 10.1016/j.combustflame.2018.01.027.

(87) D. M. Golden, G. P. Smith, A. B. McEwen, C.-L. Yu, B. Eiteneer, M. Frenklach, G. L. Vaghjiani, A. R. Ravishankara and F. P. Tully, 1998, **102**, 8598–8606, DOI: 10.1021/jp982110m.

(88) T. J. Held and F. L. Dryer, 1998, **30**, 805–830, DOI: 10.1002/(sici)1097-4601(1998)30:11<805::aid-kin4>3.0.co;2-z.

(89) C. Dombrowsky, A. Hoffmann, M. Klatt and H. G. Wagner, *Berichte der Bunsengesellschaft fur physikalische Chemie*, 1991, **95**, 1685–1687, DOI: 10.1002/bbpc.19910951217.

(90) P. Frank, K. Bhaskaran and T. Just, 1988, **21**, 885–893, DOI: 10.1016/s0082-0784(88)80320-5.

(91) K. Yasunaga, S. Kubo, H. Hoshikawa, T. Kamesawa and Y. Hidaka, 2007, **40**, 73–102, DOI: 10.1002/kin.20294.

(92) C. Cavallotti, M. Pelucchi and A. Frassoldati, *Proceedings of the Combustion Institute*, 2019, **37**, 539–546, DOI: 10.1016/j.proci.2018.06.137.

(93) E. E. Dames, 2014, **46**, 176–188, DOI: 10.1002/kin.20844.

(94) T. G. Beuthe and J.-S. Chang, *Japanese Journal of Applied Physics*, 1997, **36**, 4997–5002, DOI: 10.1143/jjap.36.4997.

(95) A. J. Dean, D. F. Davidson and R. K. Hanson, *The Journal of Physical Chemistry*, 1991, **95**, 183–191, DOI: 10.1021/j100154a037.

(96) L. M. Arin and P. Warneck, *The Journal of Physical Chemistry*, 1972, **76**, 1514–1516, DOI: 10.1021/j100655a002.

(97) D. Husain and A. N. Young, *Journal of the Chemical Society, Faraday Transactions 2*, 1975, **71**, 525, DOI: 10.1039/f29757100525.

(98) M. Burmeister and P. Roth, *AIAA Journal*, 1990, **28**, 402–405, DOI: 10.2514/3.10406.
